# Domain-specific effects of NAD^+^ precursors on fatigue and functional performance: a systematic review and network meta-analysis

**DOI:** 10.3389/fnut.2026.1890335

**Published:** 2026-09-08

**Authors:** Yixuan Gao, Shengnian Li, Jingjun Guan, Yikun Yin, Xianghao Kong, Liang Yu

**Affiliations:** 1 School of Sport Science, Beijing Sport University, Beijing, China; 2 Key Laboratory of Sports and Physical Health of Ministry of Education, Beijing Sport University, Beijing, China; 3 School of Strength and Conditioning Training, Beijing Sport University, Beijing, China; 4 Academic Affairs Office, Beijing Sport University, Beijing, China

**Keywords:** cognitive function, fatigue, NAD^+^, network meta-analysis, physical performance

## Abstract

**Background:**

Fatigue is a multidimensional syndrome involving the cognitive and physical domains, and NAD^+^-dependent biological energy dysfunction is considered a key mechanism. This study aimed to compare the efficacy of niacinamide adenine dinucleotide (NAD^+^) precursors in cognitive, motor and perceived fatigue domains using a domain-based network meta-analysis (NMA).

**Methods:**

A systematic review and NMA were conducted on randomized controlled trials (RCTs) according to the PRISMA-NMA guidelines (PROSPERO: CRD420251237193). Including adults who receive NAD^+^ precursors [niacinamide nucleotide (NR), niacinamide mononucleotide (NMN), niacinamide adenine dinucleotide (NADH) and niacinamide (NAM)]. The outcomes were categorized within the domain-based fatigue framework, divided into perceived fatigue, cognitive performance and exercise performance (endurance and strength), with functional outcomes comprising quality of life (QoL) and sleep. The standardized mean difference (SMD) is summarized, and the surface under the cumulative ranking curve (SUCRA) was calculated.

**Results:**

A total of 27 studies (*n* = 1,599) were synthesized, including 25 RCTs and 2 quasi-experimental tests. The influence of the NAD^+^ precursor is specific to the domain. No precursor can significantly improve perceived fatigue or sleep quality. In contrast, NMN showed dose-dependent enhancement in endurance (medium dose: SMD = 0.66, 95% CI 0.09–1.23; high dose: SMD = 1.02, 95% CI 0.43–1.60), while neuromuscular strength remained unchanged. For cognitive performance, NADH produced the strongest improvement (SMD = 1.50, 95% CI 0.82–2.19), especially in cognitive impairment cohorts, although this finding was sensitive to influential studies. Moderate improvement in the quality of life was observed, mainly NMN (high dose: SMD = 0.56, 95% CI 0.14–0.98; low dose: SMD = 0.45, 95% CI 0.11–0.78).

**Conclusion:**

NAD^+^ precursor supplementation exerts a selective impact on fatigue-related outcomes. This study emphasizes the domain-based NMA framework to unravel heterogeneous fatigue outcomes, in which NAD^+^ precursor prioritizes improving endurance-related performance. But with limited translation to subjective fatigue and neuromuscular strength, it supports a precision-based approach of NAD^+^ supplements that considers outcome type, dosage, and population characteristics.

**Systematic review registration:**

PROSPERO. CRD420251237193. https://www.crd.york.ac.uk/PROSPERO/view/CRD420251237193

## Graphical abstract

**Figure:**
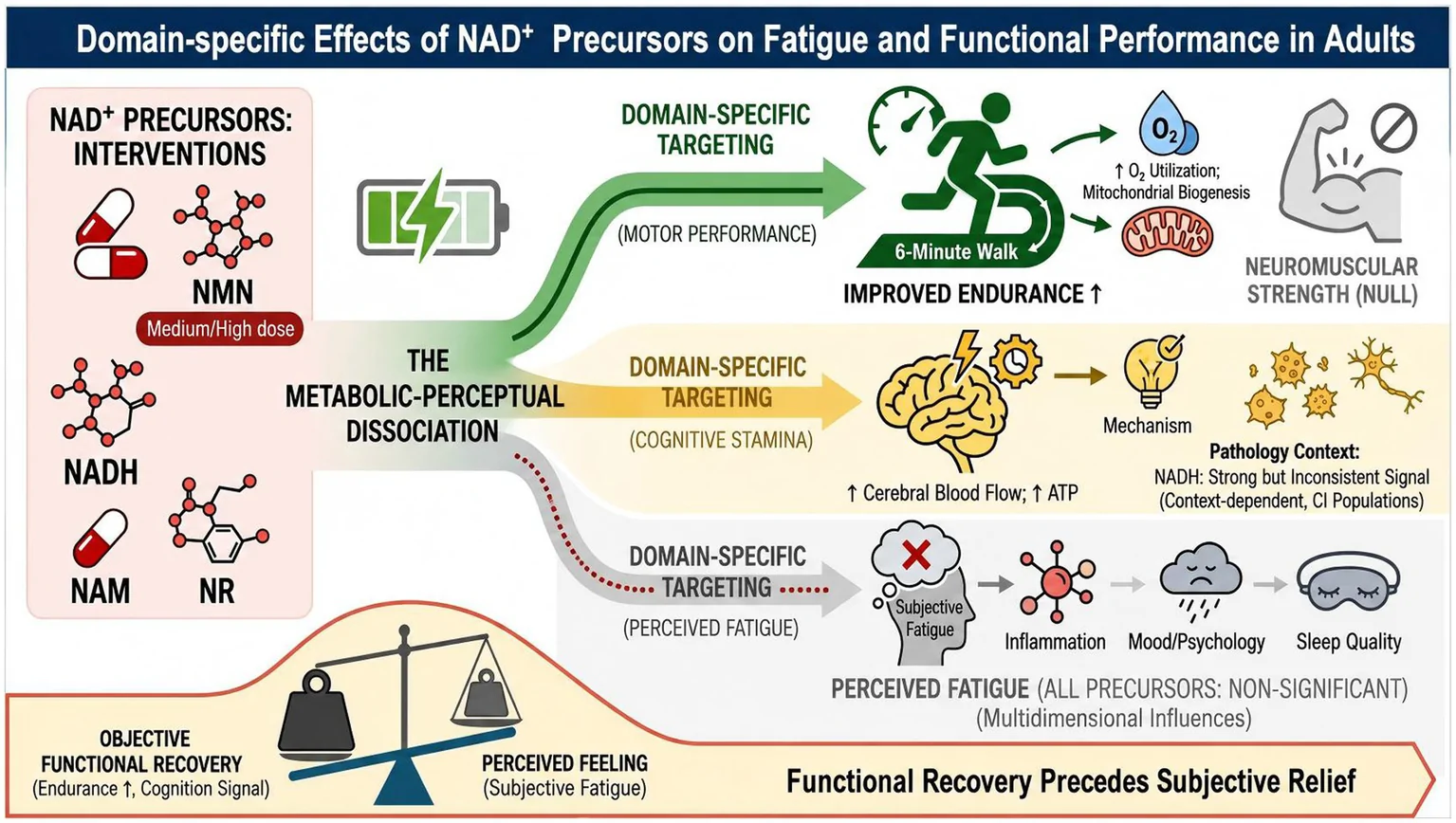

## 1 Introduction

Fatigue is a persistent and debilitating sensation of tiredness that is usually accompanied by a synchronous decline in both cognitive and physical function, which has become a formidable challenge to global public health. In the fields of sports medicine and neurobiology, fatigue is increasingly conceptualized as a task-induced multidimensional structure, often known as state fatigue, including the subjective perception of exhaustion (perceptual fatigue) and the decline in objective performance (performance fatigue) in the field of cognition and exercise (1).

Fatigue is the core symptom of long-term COVID-19, myalgic encephalomyelitis / chronic fatigue syndrome (ME/CFS) and neurodegenerative diseases, which are especially common among elderly adults (2–4). The convergence of “brain fog” state, reduced metabolic energy, and impaired motor function will severely diminish the quality of life of patients, weaken the independence of daily functions and bring a heavy economic burden on healthcare systems worldwide (5). Therefore, for clinicians and researchers, finding safe and effective interventions to alleviate fatigue symptoms in cognitive and motor fields remains a crucial priority.

Convergent evidence indicated that mitochondrial dysfunction and cellular energy impairment constitute the common pathophysiological basis of fatigue under different conditions (6–9). As an indispensable coenzyme in cell metabolism, NAD^+^ is indispensable for oxidative phosphorylation and the synthesis of adenosine triphosphate (ATP). Aging, chronic stress and various disease states are associated with a serious decline in NAD^+^ bioavailability, leading to a cellular energy crisis, manifested as impaired skeletal muscle contractility and impaired integrity of the central nervous system (10, 11). Therefore, the NAD^+^ enhancement strategy is becoming a promising but contextual treatment, emphasizing the need to go beyond indiscriminate supplements and turn to more precise adjustment (12). Given the core role of NAD^+^ in mitochondrial bioenergetics, it is assumed that this strategy can alleviate the motor and cognitive components of performance fatigue, while possibly reducing perceived fatigue.

The current intervention mainly uses four different precursors, including NR, NMN, NADH and NAM. Although the ultimate goal of these precursors is to increase systemic NAD^+^ levels, they spread through different metabolic pathways and show obvious tissue-specific distributions. For example, NADH has been proven to be particularly effective in treating neurological symptoms. As an effective reducing agent, it can directly promote neurotransmitter synthesis and neutralize oxidative stress, thus alleviating “perceptual fatigue” and cognitive “brain fog” (13, 14). On the contrary, NMN and NR are characterized by their strong ability to enhance the metabolic adaptability of peripheral tissues. These precursors use specific transporters (e.g., Slc12a8 of NMN) to accumulate in skeletal muscles, where they stimulate mitochondrial function and oxygen utilization, thereby objectively improving physical performance (15, 16). At the same time, as the basic component of the rescue route, NAM may produce a milder effect due to its feedback to inhibit the potential of NAD^+^ consumption enzyme (17).

Despite promising mechanistic underpinnings and a rapidly expanding body of clinical research, the evidence regarding the therapeutic efficacy of NAD^+^ precursors remains fragmented and inconclusive. Existing systematic reviews have often been restricted in scope, focusing on isolated clinical conditions or a limited set of outcomes, while failing to evaluate these outcomes within an integrated state fatigue framework that distinguishes perceived fatigue from cognitive and motor performance fatigue as a cohesive, functionally intertwined domain (18). It is worth noting that although there is a clear distinction between perceived fatigue, cognitive performance fatigue and exercise performance fatigue, fatigue is often treated as a single construct. This conceptual lack of clarity limits the explanatory power and comparability of the findings, especially given the known dissonance and interaction between subjective fatigue and objective performance decline (1, 19).

Furthermore, a significant knowledge gap persists due to the virtual absence of “head-to-head” RCTs directly comparing the relative potency of different NAD^+^ precursors. Consequently, the relative efficacy and ranking of these interventions remain unclear. Network meta-analysis provides an appropriate framework for comparing multiple dietary supplements when direct evidence is limited and for establishing comparative treatment hierarchies across interventions (20). Fatigue-related outcomes are multidimensional, whereas objective functional outcomes (e.g., the 6-min walk test (6MWT) and exercise capacity) are often evaluated separately from subjective fatigue scales, limiting a comprehensive understanding of intervention efficacy (21). Together, these challenges also support the growing shift toward precision nutrition, in which intervention efficacy may vary according to functional outcome domains, metabolic characteristics, and individual biological profiles (22). Accordingly, although previous reviews suggest that NAD^+^-related nutritional interventions may provide potential benefits in both healthy and clinical populations, substantial heterogeneity in fatigue definitions, outcome measures, and study designs continues to limit the certainty and clinical applicability of the available evidence (18).

In order to solve the current evidence gap, this study conducted a systematic review and NMA to compare the efficacy of different NAD^+^ precursors in adults and evaluate the results of perceived fatigue, cognitive performance fatigue, exercise performance fatigue and fatigue-related functional measures, with the aim of establishing a comparative hierarchy of interventions within the fatigue framework. By integrating a wide range of clinical evidence, ranging from healthy aging to pathological states such as ME/CFS and Long-COVID, utilizing SMD to coordinate various psychometric and functional scales. The primary objective of this study is to evaluate the comparative effect of different NAD^+^ precursors in the state fatigue framework, with particular focus on perceived fatigue and motor performance fatigue. Secondary objectives included examining their impact on cognitive performance fatigue, as well as fatigue-related functional outcomes such as health-related quality of life and sleep quality. Combined with fatigue outcome indicators across different domains, and through the rigorous integration of quantitative evidence on perceived and performance fatigue, this study provides targeted guidance for clinical decision-making and the optimization of relevant treatment strategies. At the same time, it focuses on NAD^+^ prospective interventions to provide an effective theoretical basis for future fatigue-related research.

## 2 Materials and methods

### 2.1 Protocol and registration

This systematic review and network meta-analysis has been proactively registered on PROSPERO (CRD420251237193), which is based on the 2015 NMA Extended System Review and Meta-Analysis Preferred Reporting Project guidelines (23).

### 2.2 Search strategy

A comprehensive search was conducted across nine databases: PubMed, Web of Science, Embase, PROQUEST, PsycINFO, Scopus, MEDLINE and Cochrane Library, covering the number according to all the records inception to January 1st, 2026. The search strategy combines controlled vocabulary (e.g., MeSH terms) and free text keywords to capture “NAD^+^ precursor” (e.g., NMN, NR, NADH, NAM), “fatigue” (e.g., including fatigue, lethargy), “cognitive function” and “physical performance.” In order to strengthen coverage, the reference list included in research and major clinical trial registries (Clinical Trials.gov and WHO International Registry of Clinical Trials) was also reviewed. There are no restrictions on the language or publication date. Supplementary Table S1 provides a complete database-specific search strategy.

### 2.3 Eligibility criteria

The study is selected according to the following participants, interventions, comparisons, results and research design (PICOS) standards and reporting characteristics:

1. Population (P) and reason: Adults (≥18 years old) with a broad clinical spectrum, including healthy elderly people and people with pathological fatigue or decreased function (e.g., long-term COVID-19, ME/CFS, age-related cognitive or physical disorders). This inclusive method helps to comprehensively evaluate the efficacy of NAD^+^ precursors in various baseline metabolic states and clinical conditions.
2. Intervention (I) and node clustering: Eligible interventions include direct NAD^+^ precursors - NMN, NR, NADH and NAM supplementation. For node clustering, in the therapeutic network, each precursor is defined as a separate node (NMN, NR, NADH, NAM), reflecting different mechanisms and pharmacokinetic characteristics. This clear separation allows an accurate comparison of the effects of each precursor. Non-direct precursors (such as niacin, tryptophan) and multi-component formulas that cannot be separated from precursors are excluded.
3. Comparator (C): Includes active head-to-head comparison between placebo, waiting list comparison or eligible NAD^+^ precursors. All inactive controls are integrated into a control node as a general reference for network meta-analysis.
4. Outcomes (O) and follow-up: Eligible studies reported at least one validated indicator in the following areas. The main outcomes were categorized within a domain-based fatigue framework of state fatigue, including perceived, motor, and cognitive fatigue. The primary outcomes comprised two dimensions: perceived fatigue (measured by subjective scales such as FSS, FIS, FACIT-fatigue) and performance fatigue, which was further divided into (1) motor performance: endurance-related performance (e.g., VO₂max, VO₂peak, total exercise duration, and 6MWT) and neuromuscular strength /function (e.g., grip strength, SPPB, STS). This classification was predefined because these outcomes represent different physiological constructs and functional domains of motor performance rather than interchangeable measures of the same construct (1, 24); (2) cognitive performance (e.g., MoCA, RBANS, TMT-B, HVLT). Secondary outcomes included health-related quality of life (e.g., SF-36, PSQI). There is no minimum follow-up time for capturing acute metabolic reactions and chronic functional adaptation.
5. Research design (S) and reporting characteristics: including peer-reviewed RCT and parallel group or cross-designed controlled clinical trials. Excluding non-randomized studies, observational studies, comments, conference abstracts, editorials, preprints, single-arm trials, case reports, *in vitro* or animal studies and research agreements. Considering the research from the database to January 1st, 2026, there is no language limit to minimize choice bias.

### 2.4 Data extraction and quality assessment

Search results were imported into the reference management software, in which the duplicate content is automatically eliminated and manually verified. Two independent reviewers (YG and JG) screened the title and abstract, retrieved the full text that might be eligible, and resolved the differences through discussion or third-party ruling. The data extraction work was completed independently by YY. using standardized spreadsheets. The extracted data includes research characteristics, participant profile, intervention details (NAD^+^ precursor type, dose, frequency, duration) and result indicators. When outcomes were reported at multiple time points, all relevant data were extracted for comprehensive timing analysis.

The bias risk was evaluated using the Cochrane risk bias 2 (RoB 2) tool, which included five areas: randomization, deviation from predetermined interventions, missing result data, result measurement and selection of reported results. The study is divided into three categories: low risk, certain concern or high bias. Through sensitivity analysis, the research with high bias risk is excluded. For each result, the certainty of the evidence is evaluated by the GRADE method to ensure the reliability of the results of the network meta-analysis.

### 2.5 Statistical analysis

NMA was carried out within the framework of frequencyism to synthesize the direct and indirect evidence of all contained NAD^+^ precedents at the same time. All analyses were carried out using Stata (version 18.0; Stata Corp, Texas College Station, United States) with the network and mvmeta software packages. The continuous outcomes were summarized as SMD with a 95% confidence interval (CI). Outcomes were classified according to the state fatigue framework, including perceived fatigue, cognitive performance and motor performance. Importantly, the outcomes of motor performance are further divided into endurance-related e.g., VO_2max_, VO_2peak_, total exercise duration, 6MWT and neuromuscular strength-related domains (e.g., grip strength, SPPB). The random effect model is used throughout to explain the expected clinical and methodological heterogeneity of each study. Statistical heterogeneity is evaluated using *I*^2^ statistics, and a value >50% indicates substantial heterogeneity.

1. Network construction and synthesis: We generated a network diagram to illustrate the geometry of treatment comparison. The nodes represent different NAD^+^ precursors (NMN, NR, NADH, and NAM) or the control group, and the edge thickness is directly proportional to the number of direct comparisons. For multi-arm trials, using the multivariate meta-analysis method to consider the correlation in the study to avoid analytical unit errors.
2. Consistency and heterogeneity evaluation: The consistency hypothesis between direct and indirect evidence was evaluated using the therapeutic interaction model (global inconsistency) and node segmentation analysis (local inconsistency). *p*-value > 0.05 is considered to indicate that there is no statistically significant inconsistency. The Q statistic and I^2^ value are used to further quantify the heterogeneity between studies, which is interpreted as low (25%), moderate (50%) and high (75%).
3. Subgroup and sensitivity analysis: In order to explore the potential source of heterogeneity and evaluate the robustness of the findings, subgroup analysis is carried out according to the following factors: (1) types of NAD^+^ precursors (NMN, NR, NADH, NAM); (2) population characteristics (e.g., healthy population and clinical populations); and (3) intervention duration. Sensitivity analyses were conducted by excluding studies with a high risk of bias, influential studies identified during the analysis, and quasi-experimental studies to assess the robustness of the network estimates based solely on randomized evidence. By excluding the risk of high bias and using alternative models to regulate the rerun analysis as appropriate, sensitivity analysis is carried out to evaluate the robustness of the results. In addition, if the data allowed, meta-regression was also performed to evaluate the impact of research-level covariates (treatment type, dose, duration, and participant status and disease type) on the treatment effect.
4. Ranking and publication deviation: The relative ranking of interventions is estimated using the SUCRA, and its values range from 0 to 1. The higher the value, the greater the possibility of becoming the most effective treatment. The potential small-scale research effect and publication deviation were evaluated using a relatively adjusted funnel diagram, and the asymmetry was visually checked. Egger’s regression test is applicable to the results of at least 10 studies. All statistical tests are two-way, and the significance level is set at *p* < 0.05.

## 3 Results

### 3.1 Study selection

**Figure 1:**
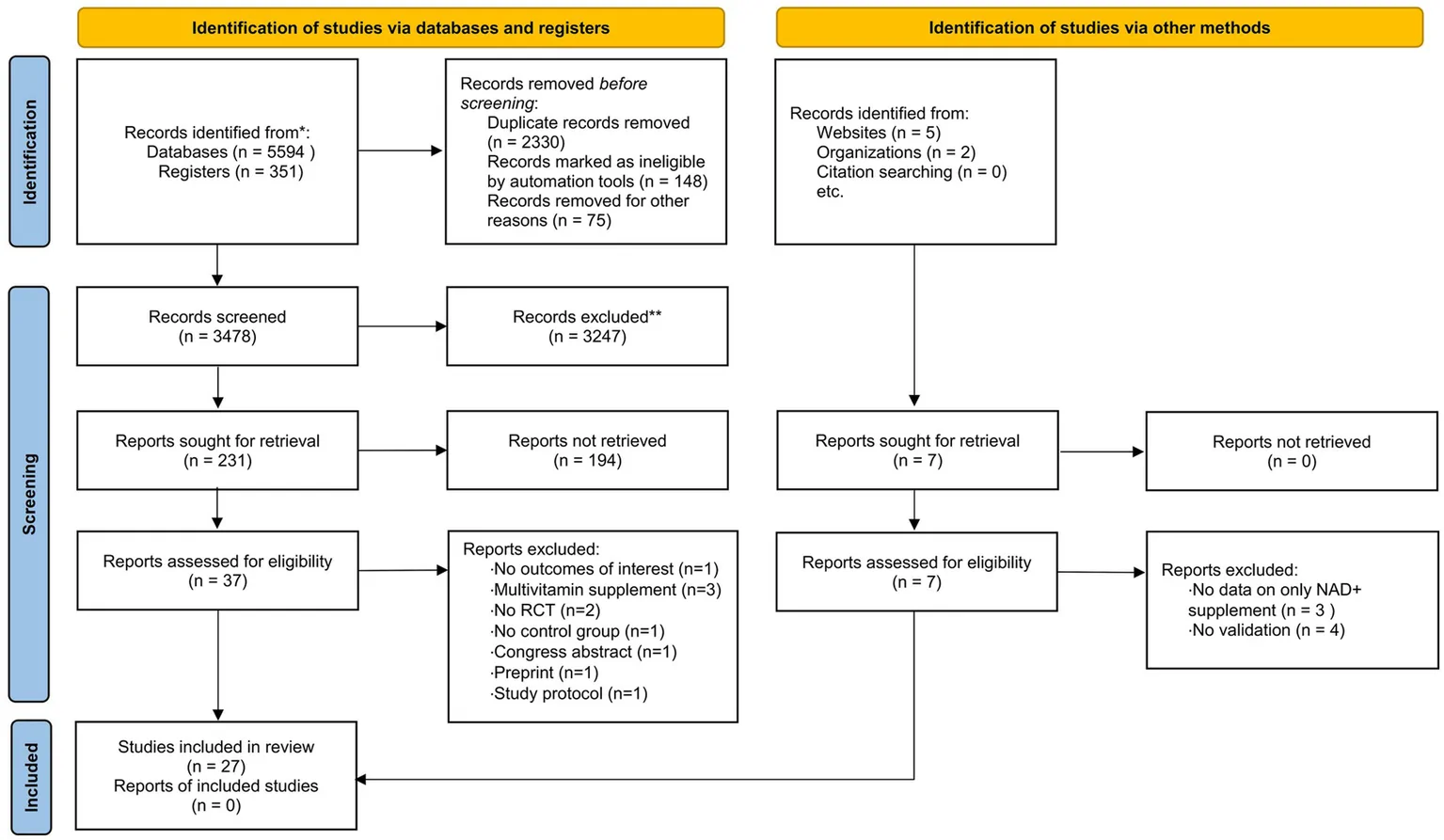
Flow chart of literature screening.

Synthesizing search results across various databases yielded 5,594 records. After eliminating duplicates and records excluded by automation tools or other reasons, 3,478 articles were included. Following a preliminary screening of titles and abstracts, 3,247 irrelevant records were excluded. Of the 231 reports sought, 194 could not be retrieved, leaving 37 to be assessed for eligibility. After a full-text review, 11 reports were further excluded for specific reasons. Additionally, 7 studies were identified through other methods (websites and organizations). Ultimately, 27 studies were included in the systematic review and network meta-analysis (3, 13, 14, 25–48). The study selection process is shown in Figure 1.

### 3.2 Study characteristics

A total of 27 studies (25 RCTs and 2 quasi-experimental tests) involving NAD^+^ precursor supplementation were included in this system evaluation and network meta-analysis. These trials recruited a total of 1,599 subjects, including 842 in the treatment group and 757 in the control group. The average age of the subjects is between 24 and 83 years old, and most studies focus on middle-aged and elderly people. The research subjects cover healthy people and clinical patients, including chronic fatigue syndrome, Parkinson’s disease, chronic kidney disease, peripheral artery disease, diabetes, mild cognitive impairment, Alzheimer‘s disease and long-term COVID-19 patients. The study sample includes both male and female subjects. Some studies set up gender-specific cohorts, such as studies that only include male subjects.

The NAD^+^ precursors investigated included NR, NMN, NADH, and NAM. In the included trials, 6 studies (224 participants) examined the effects of NADH, 12 studies (459 participants) examined the effects of NR, 7 studies (370 participants) examined the effects of NMN, and 2 studies (546 participants) examined the effects of NAM. Twenty five included studies adopted a two-arm design (precursor vs. placebo), and 2 studies directly compared three different doses of NAD^+^ precursors head-to-head. NR and NMN were the most frequently studied interventions, typically administered at doses of 250–3,000 mg/day. NADH doses ranged from 10 to 30 mg/day, and NAM doses reached up to 3,000 mg/day. Supplementation durations varied from 2 to 48 weeks, though most trials lasted 4 to 12 weeks.

The trials measured fatigue-related and functional performance outcomes. Perceived fatigue was measured with tools such as the Fatigue Severity Scale (FSS), Fatigue Impact Scale (FIS), FACIT-Fatigue, and Profile of Mood States. Motor performance outcomes included aerobic capacity and endurance, such as VO_2max_, VO_2peak_, 6MWT and muscle strength, such as hand-grip and knee extension strength. Cognitive ability was assessed using global and specific neuropsychological tests, including the MoCA, RBANS, and Trail Making Test-B. Some studies also examined quality of life and sleep, mostly using the SF-36 and the Pittsburgh Sleep Quality Index (PSQI). The detailed study characteristics of the included studies are presented in Supplementary Table S2.

### 3.3 Risk of bias

The methodological quality of the 27 included studies was evaluated using the Cochrane Risk of Bias 2 tool for the intention-to-treat effect. The proportions of studies with low, moderate (some concerns), and high risk of bias for each domain were as follows: randomization process (88.9, 11.1, and 0%, respectively); deviations from intended interventions (85.2, 11.1, and 3.7%, respectively); missing outcome data (85.2, 3.7, and 11.1%, respectively); measurement of the outcome (96.3, 3.7, and 0%, respectively); and selection of the reported result (85.2, 7.4, and 7.4%, respectively). Overall, 66.7% of the studies were classified as low risk, 22.2% were classified as moderate risk, and 11.1% were classified as high risk. The main sources of potential deviations are incomplete result data (high risk of 11.1%) and selective reports (7.4% high risk), mainly due to the lack of test registration or incomplete reports of all pre-specified results. For detailed bias risk assessment, see Appendix 3.

### 3.4 Network geometry

**Figure 2:**
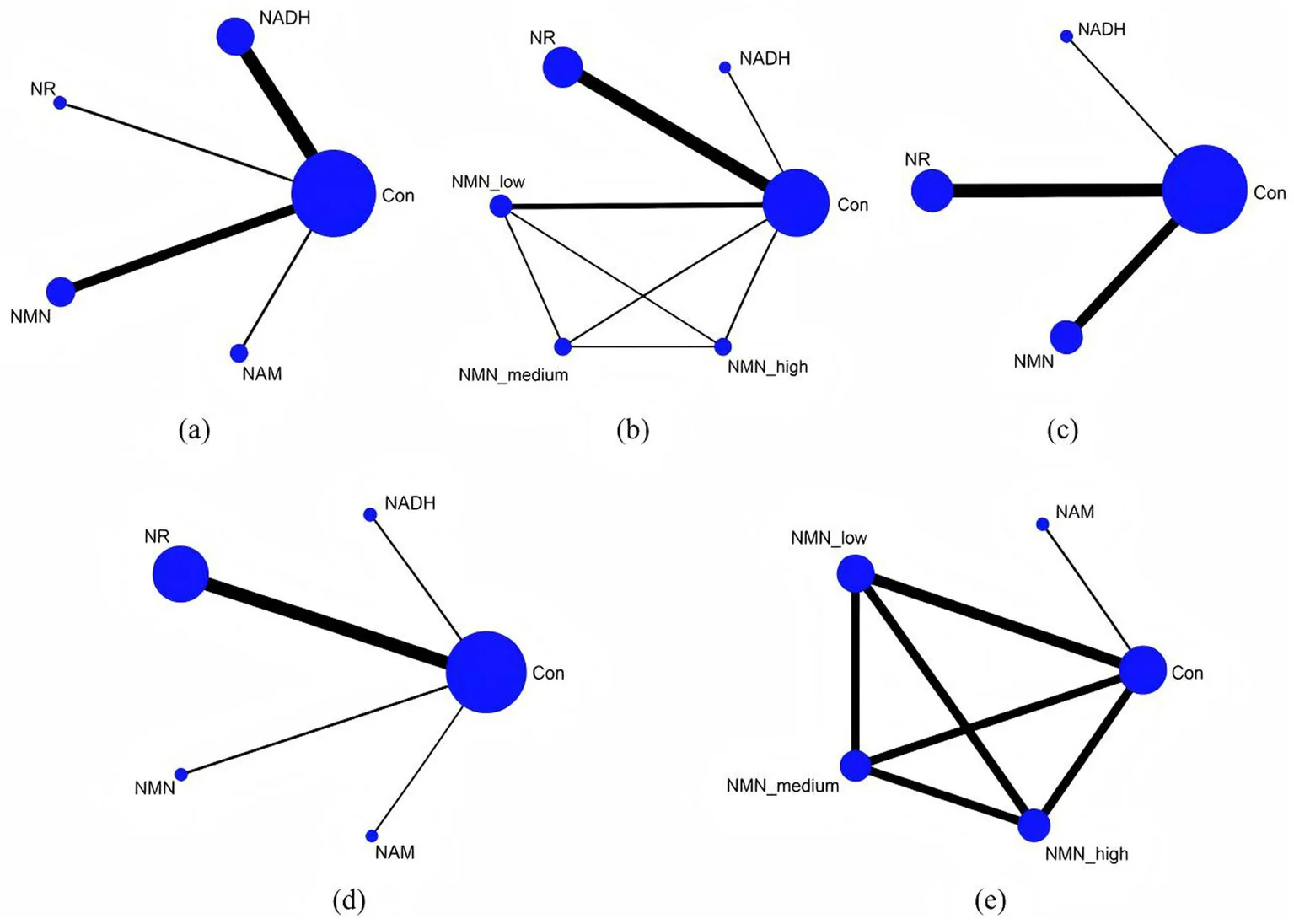
Network geometry for the network meta-analysis. Network diagrams for **(a)** Perceived fatigue, **(b)** Aerobic capacity and endurance, **(c)** Neuromuscular strength, **(d)** Cognitive performance, and **(e)** Quality of life. Each node represents an intervention, while the connecting lines between two nodes indicate one or more RCTs directly comparing those interventions. Node size reflects the number of participants assigned to each intervention, and line thickness corresponds to the number of RCTs comparing the interventions connected by the line.

Figure 2 presents the network geometry for each outcome in the network meta-analysis. In each network diagram, the node corresponds to a specific intervention, and the size of the node is directly proportional to the total number of registered subjects. The line represents a direct head-to-head comparison, and the thickness of the line reflects the number of relevant studies. Separate networks analysis were constructed for perceptual fatigue (Figure 2a), aerobic ability and endurance (Figure 2a), neuromuscular strength (Figure 2c), cognitive performance (Figure 2d) and quality of life (Figure 2e). Due to insufficient connectivity, sleep quality was excluded from network meta-analysis, but uses paired meta-analysis for analysis. Most outcome networks were placebo-centered, indicating that most of the evidence comes from indirect comparisons through common control groups. The perceived fatigue, neuromuscular strength and cognitive performance networks exhibited strict star-shaped structures with no direct comparison between active NAD^+^ precursors. By contrast, the network of aerobic capacity and quality of life networks contained closed loops formed by direct comparisons among active interventions, allowing both direct and indirect evidence to contribute to the network estimates.

### 3.5 Effects on perceived fatigue

**Figure 3:**
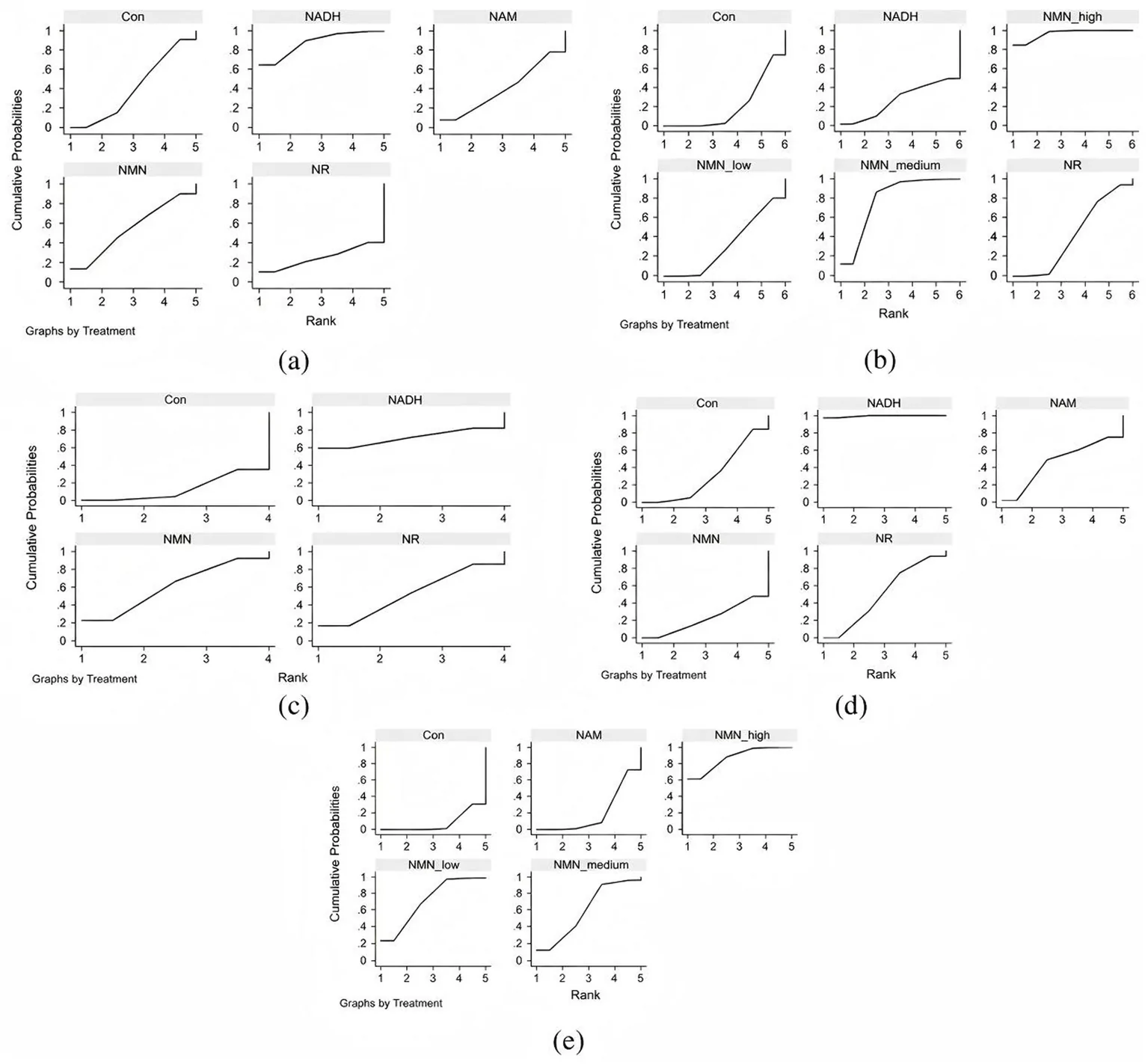
Cumulative ranking probability curves for the primary outcomes. Subplots represent **(a)** Perceived fatigue, **(b)** Aerobic capacity and endurance, **(c)** Neuromuscular strength, **(d)** Cognitive performance and **(e)** Quality of life. The horizontal axis displays the possible ranks (best to worst), while the vertical axis shows the aggregate probability of a treatment occupying a given rank or any higher position.

**Figure 4:**
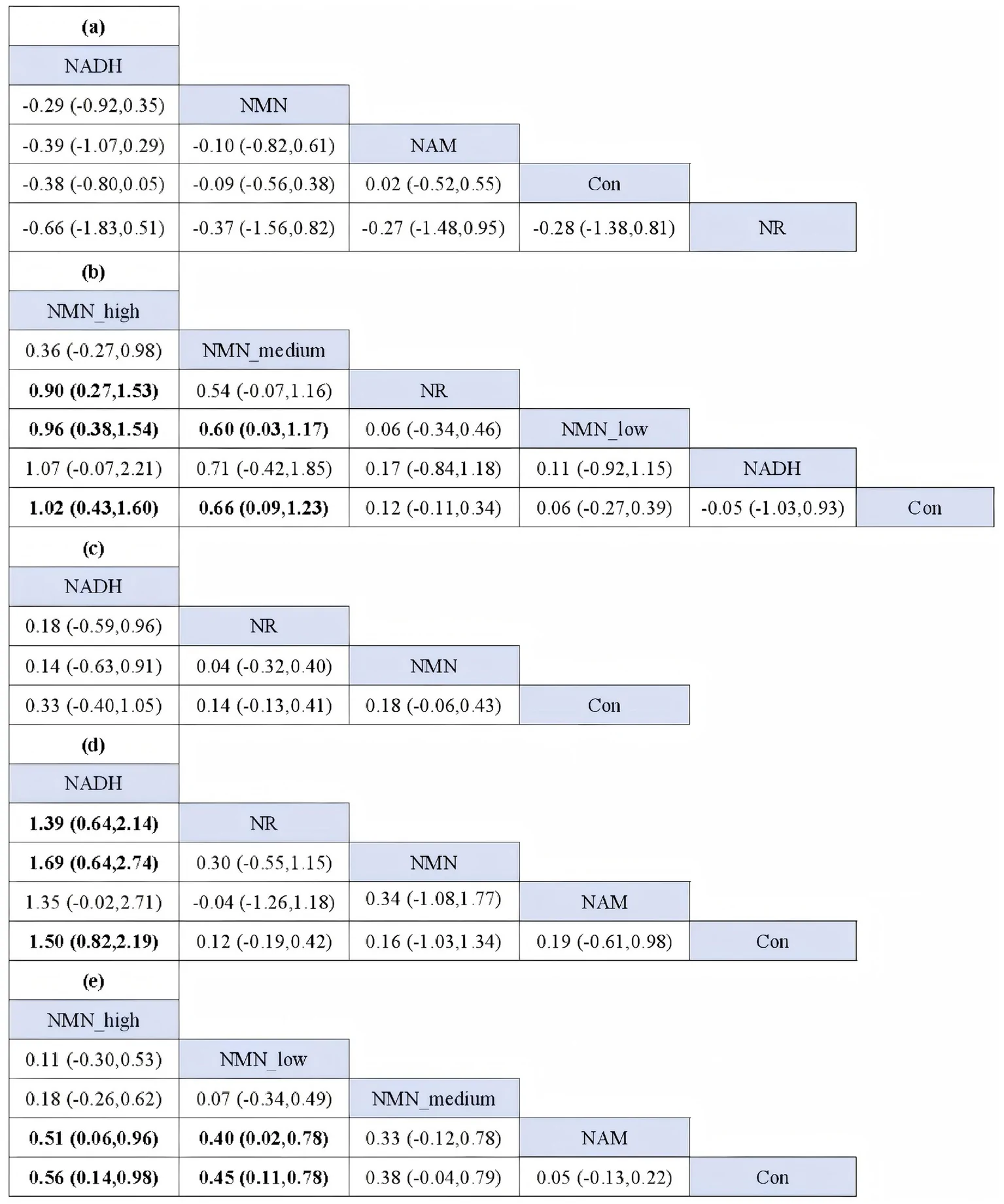
League tables for pairwise comparative effectiveness. Results are presented for **(a)** perceived fatigue, **(b)** aerobic capacity and endurance, **(c)** neuromuscular strength, **(d)** cognitive performance and **(e)** quality of life. Each cell represents the standardized mean difference (SMD) with its corresponding 95% confidence interval (CI). In any comparison, a negative SMD favors the intervention in the upper-left, whereas a positive SMD favors the intervention in the lower-right. Statistically significant findings are highlighted in bold. CI, confidence interval; Con, control; NADH, nicotinamide adenine dinucleotide hydride; NAM, nicotinamide; NMN, nicotinamide mononucleotide; NR, nicotinamide riboside; SMD, standardized mean difference.

A comprehensive analysis of 16 studies involving 1,126 participants found no statistically significant improvement in perceived fatigue for any NAD^+^ precursors compared with placebo under the random-effects network model. The inconsistency test of network analysis demonstrated no evidence of global inconsistency (*p* = 0.2142; Appendix 4.1.2). Among all interventions, NADH ranked highest and showed the largest estimated treatment effect (SMD = −0.38, 95% CI − 0.80 to 0.05; Figures 3a, 4a), suggesting a possible trend toward benefit; however, the confidence interval crossed the null and between-study heterogeneity was substantial. Indirect comparisons between active interventions were not statistically significant. All network results included the possibility of no effect. Pairwise meta-analysis likewise showed a small, non-significant reduction in perceived fatigue is very small and not-significant (SMD = −0.15, 95% CI − 0.33 to 0.03; *I*^2^ = 68.7%; Appendix 4.1.1). Subgroup analysis also shows that there is no significant benefit to pathology or age-related fatigue (Appendix 4.1.3, 4.1.4). Sensitivity analysis indicated that the estimated effect of NADH was influenced by several individual studies, and exclusion of these studies attenuated the treatment effect and altered the treatment ranking, indicating that the apparent superiority of NADH should be interpreted cautiously (Appendix 4.1.5).

### 3.6 Effects on performance fatigue

#### 3.6.1 Motor performance fatigue

##### 3.6.1.1 Aerobic capacity and endurance

Fourteen studies involving 540 participants were included in a network with multiple closed-loop connections, allowing direct and indirect comparisons between interventions (Figure 2b). Statistical heterogeneity is negligible (*I*^2^ = 0%). Inconsistency tests revealed no evidence of global (*p* = 0.1457) or local inconsistency; the detailed results are shown in Appendix 4.2.2. Compared with placebo, high-dose NMN improved aerobic capacity (SMD = 1.02, 95% CI 0.43 to 1.60; Figure 4b), and medium-dose NMN also showed benefit (SMD = 0.66, 95% CI 0.09 to 1.23; Figure 4b). In indirect comparison, high-dose NMN is better than NR and low-dose NMN. The cumulative ranking probability curve (Figure 3b) shows the obvious separation between NMN dose groups. High-dose NMN showed the probability of ranking first, followed by medium-dose NMN, indicating the dose-reaction relationship.

The pairing analysis is consistent with the network estimate in direction and shows that the summary improvement of aerobic capacity and endurance is mainly driven by medium and high doses of NMN. In the dose-stratified pairing analysis, low-dose NMN showed no significant effect, while medium-dose NMN showed significant benefits (SMD = 0.63, 95% CI 0.01 to 1.24). High-dose NMN produced the maximum effect estimate (SMD = 0.80, 95% CI − 0.01 to 1.61), although this did not reach the traditional statistical significance (Appendix 4.2.1).

The sensitivity analysis did not substantially change the overall conclusion. Exclusion of key high-dose studies weakened the effect, but did not change its direction. Similarly, the exclusion of all medium-dose or all high-dose NMN studies makes the remaining high-dose categories statistically significant, and there is no residual heterogeneity (*I*^2^ = 0%; *τ*^2^ = 0). Overall, these findings support the strong and dose-dependent effects of NMN on endurance-related outcomes. Appendix 4.2 provides more details.

##### 3.6.1.2 Neuromuscular strength

Twenty studies involving 619 participants were included in a placebo-centered star-shaped (Figure 2c). No evidence of global inconsistency was detected (*p* = 0.1985), and heterogeneity was low to moderate (*I*^2^ = 26.84%; *τ*^2^ = 0.0202). For detailed results, please refer to the Appendix 4.3.2. Compared with placebo, all interventions did not show a statistically significant improvement in neuromuscular strength. The effect estimates are relatively mild and do not exceed the zero value boundary in all comparisons, including NADH (SMD = 0.33,95% CI − 0.40 to 1.05), NMN (SMD = 0.18,95% CI − 0.06 to 0.43) and NR (SMD = 0.14,95% CI − 0.13 to 0.41; Figure 4c). Although the ranking probability (Figure 3c) indicates that NADH is most likely to be the most effective intervention, it should be interpreted with caution, because no intervention is statistically significantly superior to placebo or other precursor substances.

The matching meta-analysis is consistent with the network estimate, and does not show the overall benefit (SMD = 0.14, 95% CI − 0.05 to 0.34; Appendix 4.3.1). Subgroup analysis also shows that NADH, NR or NMN has no significant aggregation effect. The sensitivity analysis did not substantially change these findings, indicating that the empty results were robust. Overall, current evidence does not support the consistent or clinically meaningful benefits of NAD^+^ precursor supplements for neuromuscular strength outcomes. Appendix 4.3 provides more details.

#### 3.6.2 Cognitive performance fatigue

A total of 24 studies (involving 788 participants) constitute the star network of placebo centers (Figure 2d). No global inconsistency was found (*p* = 0.805), and the degree of heterogeneity was moderate (*I*^2^ = 63.1%; *τ*^2^ = 0.26); see Appendix 4.4.2 for detailed results. Compared with placebo, NADH significantly improved cognitive performance (SMD = 1.50, 95% CI 0.82–2.19; Figure 4d), while NR, NMN and NAM did not show the effect. Indirect comparison further shows that NADH is superior to NR and NMN. The sorting results are strongly inclined to NADH, followed by NR, NAM and NMN (Figure 3d). The subgroup analysis according to the baseline cognitive status shows that in the participants with impaired cognitive function, the effect of NADH is significantly better than that of placebo (SMD = 2.31, 95% CI 1.04–3.59; *I*^2^ = 46.7%; see Appendix 4.4.3, 4.4.4 for details), whereas no intervention was effective in cognitively normal (CN) participants.

Pairwise meta-analysis showed a slight but statistically significant overall improvement in cognitive performance (SMD = 0.30, 95% CI 0.01–0.59; *I*^2^ = 74.5%; Appendix 4.4.1), although this effect was largely attributable to studies evaluating NADH. Influence analyses demonstrated that the magnitude of the NADH effect varied after exclusion of individual influential studies (13, 29), suggesting that the treatment-specific effect estimate remained sensitive to individual trials despite an unchanged treatment ranking. An additional sensitivity analysis restricted to randomized controlled trials slightly attenuated the pooled pairwise estimate (SMD = 0.22, 95% CI − 0.07 to 0.52), whereas the network estimate for NADH remained statistically significant (SMD = 1.44, 95% CI 0.89–1.99). Network heterogeneity also decreased substantially (*I*^2^: 63.1 to 44.1%; *τ*^2^: 0.260 to 0.080), supporting the robustness of the network findings despite attenuation of the pairwise effect (Appendix 4.4.5).

NADH consistently ranked first for cognitive performance outcomes. The largest treatment effect was observed among participants with baseline cognitive impairment, whereas the estimated effect size remained sensitive to influential studies despite stable treatment rankings across sensitivity analyses (Appendix 4.4 for additional details). Meta-regression further indicated that precursor type accounted for part of the between-study variability (*p* = 0.007; *I*^2^ = 34.9%; Appendix 4.4.7).

### 3.7 Fatigue-related functional measures

#### 3.7.1 Quality of life

A total of six studies (involving 722 participants) have built an associated network containing multiple closed loops of quality of life assessment. The network is mainly based on SF-36 scale-related indicators, which can achieve direct and indirect relationships between different interventions (Figure 2e). No inconsistency in the overall data was found (*p* = 0.7313). For detailed results, please refer to the Appendix 4.5.2. Compared with placebo, high-dose NMN (SMD = 0.56,95% CI 0.14–0.98) and low-dose NMN (SMD = 0.45, 95% CI 0.11–0.78) can improve the quality of life, while medium-dose NMN (SMD = 0.05,95% CI -0.13–0.22) and NAM (SMD = 0.38, 95% CI -0.04–0.79) have no significant effect. The difference between active interventions is generally small (Figure 4e). The sorting results support the main analytical conclusions: high-dose NMN ranks first, followed by low-dose NMN and medium-dose NMN (Figure 3e). Pairwise analysis shows that the overall improvement in quality of life is mainly due to NMN-based interventions, while sensitivity analysis does not significantly change the direction of the effect. Despite the limited improvement, the overall trend is stable. Overall, these findings suggest that NAD^+^ precursor supplements (especially NMN and NAM) may have a moderate and quantifiable effect on health-related quality of life (Appendix 4.5 for additional details).

#### 3.7.2 Sleep quality

The evaluation of sleep quality only uses pair meta-analysis, because the network is too sparse to carry out meaningful network meta-analysis. A total of four studies involving 231 subjects were included in the study. No overall benefit was observed, the estimated amount of effect was relatively small, and the confidence interval crossed the zero value. The heterogeneity between studies is low (*I*^2^ = 42.4%), and the visual inspection of forest maps and scatter maps did not show significant anomaly effects (Appendix 4.6). In general, the existing evidence does not support that supplementing NAD^+^ precursor can bring about clinically significant sleep quality improvements.

## 4 Discussion

### 4.1 Principal findings

In summary, this network meta-analysis suggests that the effects of NAD^+^ precursors are domain-specific rather than uniform across fatigue-related outcomes. No NAD^+^ precursor showed a statistically robust benefit for perceived fatigue, although NADH demonstrated the most favorable ranking and a potentially clinically meaningful signal. However, this estimate remained imprecise and should be interpreted cautiously because of substantial heterogeneity and sensitivity to influential studies. In contrast, NMN—particularly at medium and high doses showed the clearest and most robust benefit for endurance-related motor performance, whereas no consistent benefit was observed for neuromuscular strength. NADH showed the strongest signal for cognitive performance, particularly in cognitively impaired participants, but this finding was sensitive to influential studies. Quality-of-life benefits were modest, mainly observed with NMN and NAM, whereas no meaningful improvement in sleep quality was observed. Overall, these findings indicate a selective response pattern, where perceived, motor, and cognitive fatigue may respond differently to specific NAD^+^ precursor types, doses and population characteristics.

This study extends previous meta-analyses by applying a domain-based approach that separates different dimensions of fatigue, thus revealing the stratified and context-related response patterns of NAD^+^ precursor supplementation, which is covered up by treating fatigue as a single unified result. From a practical point of view, these findings suggest that supplementing a NAD^+^ precursor may be more relevant to improving endurance-related functional ability than relieving subjective fatigue. This distinction may be especially important in people with declining physical performance or metabolic dysfunction, where the biological energy restrictions are more obvious. In contrast, the expected benefits may be limited in people where fatigue is more driven by psychological, inflammatory or sleep-related factors.

### 4.2 Effects on motor performance fatigue

Our network meta-analysis demonstrated the clearest evidence of benefit for endurance-related motor performance, whereas neuromuscular strength remained largely unchanged across the included interventions. Medium- and high-dose NMN consistently ranked highest for endurance-related outcomes, whereas no NAD^+^ precursor demonstrated a consistent improvements in neuromuscular strength-related measures. These findings suggest that the effects of NAD^+^ precursor supplementation may be domain-specific rather than universal across all aspects of motor performance.

A plausible physiological explanation is that endurance performance depends primarily on mitochondrial oxidative metabolism, ATP regeneration, and resistance to metabolic fatigue, all of which are closely linked to intracellular NAD^+^ availability. Declining NAD^+^ levels associated with aging, chronic disease, and physiological stress may impair mitochondrial respiration and energy production, thereby contributing to fatigue and reduced physical function (18, 49, 50). As an essential cofactor in glycolysis, the tricarboxylic acid cycle, and oxidative phosphorylation, NAD^+^ plays a central role in maintaining ATP production during prolonged exercise (38). Consequently, restoration of NAD^+^ availability through precursor supplementation may preferentially improve endurance-related outcomes by supporting oxidative metabolism and sustained energy supply.

This interpretation is broadly supported by emerging evidence from human intervention studies. Several randomized trials have reported improvements in walking performance and sub-maximal aerobic capacity following NMN supplementation, with the largest benefits observed at medium and high doses (41, 46). Dose response analysis further shows that the increase in NAD^+^-related metabolites is associated with the increase in clinical significance of walking distance, strengthening the link between NAD^+^ recovery and functional endurance improvement (41). Although NR has also been associated with improvements in walking distance or habitual physical activity in some populations (32, 47), these effects were not consistently confirmed in the present network meta-analysis. Conversely, most human studies evaluating neuromuscular strength have reported neutral findings despite occasional improvements in fatigue-related outcomes (36, 39, 40).

Another possible explanation is that NAD^+^ precursor supplementation may improve peripheral metabolic efficiency rather than central cardiopulmonary function. For example, Liao et al. reported that NMN supplementation improved ventilatory threshold-related indices during submaximal exercise without altering VO_2max_, O_2_ pulse or ΔVO_2_/ΔWR, indicating enhanced skeletal muscle oxygen utilization rather than increased central oxygen delivery (51). If confirmed, such a mechanism is consistent with the outcome pattern observed in our analysis because endurance-related performance depends not only on maximal oxygen transport but also on the efficiency with which working skeletal muscle extracts and utilizes oxygen during prolonged exercise. However, this interpretation should be considered biologically plausible rather than directly demonstrated by the present analysis.

These findings are also consistent with the broader literature on NAD^+^ precursors. Previous reviews of physical performance and weakness results reported basically neutral findings, especially for strength-related measures and also emphasized the short duration, small sample and obvious methodal heterogeneity. Baker et al. further concluded that there is currently insufficient evidence to determine whether NAD^+^ precursor supplementation improves physical performance or physical frailty in humans, and suggested that future trials should be longer, larger, and focused on older adults with skeletal muscle dysfunction (52). Against this background, our findings help to improve the current evidence base, indicating that the functional effect of NAD^+^ precursor supplementation is selective, not universal: endurance-related sports fatigue seems to be more responsive, and the maximum strength may depend on other adaptive drivers, including resistance load, structural weight Plastic and long-term neuromuscular adaptation.

Experimental and preclinical studies provide several hypotheses that may explain these observations but should not be interpreted as direct evidence supporting the present clinical findings. Proposed mechanisms include enhanced mitochondrial biogenesis through SIRT1/PGC-1α signaling, improved skeletal muscle oxidative capacity, reduced oxidative stress, enhanced peripheral oxygen utilization, and improved vascular adaptation following NAD^+^ precursor supplementation (16, 30, 53–55). Although these mechanisms are biologically plausible, further well-designed human mechanistic trials are required to determine whether they mediate the functional improvements observed in endurance performance.

Overall, the current evidence suggests that NAD^+^ precursor supplementation may preferentially improve endurance-related aspects of motor performance, whereas evidence for neuromuscular strength remains limited. The biological mechanisms discussed above should therefore be regarded as hypothesis-generating rather than explanatory evidence.

### 4.3 Effects on perceived fatigue, cognitive performance, and related quality-of-life and sleep measures

Contrary to the findings that were relatively consistent with the observed athletic performance, the results associated with perceived fatigue, quality of life and sleep showed greater variability and weaker overall impact after supplementing NAD^+^ precursors. These areas seem to be more heterogeneous and context-dependent, which shows that the benefits of NAD^+^ mediation may not be as strong at the level of subjective perception as at the level of physical function.

Regarding perceptual fatigue, our analysis did not demonstrate statistically robust improvements following NAD^+^ precursor supplementation. Although NADH ranked highest according to SUCRA, the corresponding confidence interval narrowly crossed the null and the estimated effect remained sensitive to influential studies. Therefore, this finding should be interpreted as suggestive rather than conclusive. This pattern is broadly consistent with previous clinical evidence. Early studies reported subjective improvement subjective improvement after low-dose of NADH supplementation in chronic fatigue syndrome (14). However, subsequent large-scale trials failed to reproduce these effects, and there was no significant difference in the severity of fatigue or dysfunction compared with placebo (26). Likewise, evidence for NR and NAM remains limited or inconsistent across populations. For example, NR did not significantly improve fatigue severity in Long-COVID populations (48), although modest improvements in non-motor symptoms have been reported in specific clinical contexts such as Parkinson’s disease (27). Collectively, these mixed findings indicate that the current evidence for perceived fatigue remains inconclusive.

Importantly, this inconsistency may reflect the inherent multidimensionality of perceived fatigue. Unlike endurance performance, which is closely related to mitochondrial oxidative metabolism and energy supply, subjective fatigue arises from the interaction of metabolic, inflammatory, neuroimmune, psychological, and sleep-related processes (56). Experimental studies from the exercise-induced fatigue model indicates that HIF-1/FoxO signaling is involved in stress adaptation and metabolic regulation, whereas TNF/NOD-like receptor signaling and dysfunction of the brain-gut axis contribute to inflammatory and central-fatigue processes (57, 58). Emerging evidence further highlights the roles of gut-brain communication and neuroimmune regulation in the development and persistence of fatigue-related symptoms (59, 60). Although these studies did not directly investigate NAD^+^ precursors and were therefore not included in the quantitative synthesis, they support the concept that fatigue-related symptoms may be influenced by broader metabolic, inflammatory, neuroimmune, and gut-brain mechanisms that are not fully addressed through restoration of NAD^+^ availability alone. Therefore, the improvement of cellular bioenergetics following NAD^+^ restoration may not necessarily translated into measurable changes in subjective fatigue ratings.

In contrast, the results of quality of life—especially those evaluated using multidimensional tools such as SF-36 seem to be more responsive to NAD^+^ precursor supplementation. Several studies reported that after supplementing NMN, vitality (38, 46, 61), general health and physical role function were improved, especially when taken in moderation. Similarly, NAM is related to the improvement of physical function and the recovery of daily activities in some clinical populations (45). A reasonable explanation is that the quality of life measures capture a broader range of functional adaptations, including improved daily activity capacity and physical independence, which may not be fully reflected in the fatigue-specific scale.

Several biologically plausible mechanisms may contribute to the apparent discrepancy between improvements in objective functional outcomes and the limited effects on perceived fatigue and sleep, although these mechanisms were not directly evaluated in the present review. First, restoration of NAD^+^ availability may preferentially improve mitochondrial oxidative phosphorylation, ATP production, and skeletal muscle metabolic efficiency, thereby enhancing physical function and daily activity before measurable changes occur in subjective fatigue perception. Second, perceived fatigue is influenced by multiple biological and psychological processes, including systemic inflammation, neuroimmune regulation, and emotional status. Although NAD^+^ precursors have been reported to modulate inflammatory pathways, the magnitude of these changes observed in clinical studies may be insufficient to produce clinically meaningful improvements in fatigue perception. Third, NAD^+^ is involved in circadian regulation through SIRT1-dependent signaling, and preliminary evidence suggests that NMN supplementation may influence daytime function or alertness; however, these effects appear to be highly dependent on intervention timing and duration and may not translate into measurable improvements in overall sleep quality. Finally, a physiological threshold effect may also exist, in which increases in NAD^+^ availability must exceed a threshold before functional benefits become clinically detectable, whereas subjective fatigue and sleep outcomes may require greater or more sustained biological changes than objective performance measures (42). Collectively, these mechanisms provide biological plausibility for the heterogeneous response patterns observed across outcome domains but should be regarded as hypothesis-generating rather than direct explanations of the findings of the present network meta-analysis.

In summary, these findings show that the response mode in fatigue-related fields is selective rather than unified. NAD^+^ precursor supplements may first alleviate bioenergy damage that limits physical activity and daily functions, thus generating earlier gains in terms of endurance-related performance and function-related quality of life. In contrast, before there is a measurable improvement, more complex neuropsychological outcomes, such as perceived fatigue and sleep quality, may require longer intervention time, more targeted populations or other mechanisms beyond metabolic recovery. Therefore, the differences between the results should not be regarded as contradictory but reflect the differences in the physiological and psychological areas that are most likely to respond to NAD^+^-related interventions. The effect of NAD^+^ precursor on cognitive performance appears more promising than that on perceptual fatigue, although the current evidence is still limited and not completely reliable. In network meta-analysis, NADH shows the largest estimated effect on cognitive performance, particularly in participants with fatigue-related or pathological conditions. However, this finding should be interpreted cautiously because it was based on a limited number of heterogeneous studies. Although exclusion of the two quasi-experimental studies did not materially alter the overall network estimates or treatment rankings, the apparent superiority of NADH was attenuated after removal of influential NADH trials, indicating that the instability primarily reflected study-specific effects rather than study design.

This pattern is biologically plausible because NADH directly participates in mitochondrial oxidative phosphorylation and neuronal redox homeostasis, processes essential for sustaining cognitive function under metabolic stress. Clinical evidence suggests that these effects may be most pronounced in cognitively impaired populations (29, 62). Such mechanisms may be especially relevant in populations with impaired energy metabolism, including those with neurodegenerative conditions. Early exploratory studies have shown that NADH supplements may be beneficial in some areas of cognitive abilities of people with cognitive disabilities (29). However, recent randomized controlled trials of NAD^+^ precursors (e.g., niacinamide riboside or niacinamide) generally showed target participation and acceptable safety characteristics, but failed to show a consistent improvement in standardized cognitive outcomes, although the impact on disease-related biomarkers was small (47). This difference shows that although the regulation of NAD^+^ metabolism is mechanism-related, its conversion into measurable cognitive benefits is still limited and depends on the context.

For NR, the current evidence shows that the safety is good, but the effectiveness of traditional cognitive endpoints such as MoCA or RBANS scores is limited. However, new findings suggest that NR may produce more subtle neurobiological effects, including regulating cerebral blood flow and reducing pathological biomarkers, such as pTau (63). Similarly, in the long-term COVID-19 population, although the main cognitive results have not improved significantly, exploratory analysis shows that there are moderate benefits in performing functional tasks such as TMT-B performance, which may reflect partial relief of cognitive fatigue (48). In summary, these findings show that cognitive results are similar to perceptual fatigue and highly depend on the context. In this analysis, the obvious benefits of NADH may reflect a population-specific effect, which is more easily detected in individuals with original cognitive dysfunction than in general cognitive enhancement in healthy people. This is consistent with a broader pattern observed in the subjective field, in which the response seems to depend on baseline damage and disease status.

### 4.4 Dose- and population-specific responses to NAD + precursors

One of the most important findings of this study is that the effectiveness of NAD^+^ precursor supplements seems to depend largely on dosage and population background, rather than reflecting the uniform effect of different conditions. This is especially obvious for NMN in the field of aerobic endurance and ability.

Medium- and high-dose NMN consistently demonstrated more robust and reproducible improvements compared with lower doses across analyses. These findings are consistent with prior dose-ranging trials showing that higher NMN doses (e.g., 600–900 mg/day) are associated with greater improvements in walking performance and function-related quality-of-life outcomes, as well as ventilatory threshold-related parameters (51). Such a pattern supports the existence of a physiological “threshold effect,” in which NAD^+^ availability must reach a functionally meaningful level before measurable improvements in performance or functional capacity occur (64). This dose dependency is further supported by the complex pharmacokinetics of NAD^+^ precursors. Oral compounds undergo extensive metabolic transformation, microbial processing and tissue-specific distribution, which means that equivalent nominal doses may not produce a comparable increase in intracellular NAD^+^ of different individuals (3, 65).

Population specificity is another key determinant of responsiveness. Cumulative evidence shows that NAD^+^ precursor supplements are more effective in conditions characterized by NAD^+^ deficiency, metabolic dysfunction, aging-related decline, or disease burden, and the impact on young people or metabolically healthy individuals is usually small. For example, NR has shown limited impact on exercise performance in healthy populations but may improve aerobic capacity in older adults (31). A similar principle may explain the domain-specific findings observed in this study. NADH has a relatively stronger effect on cognitive results, especially in people with cognitive disabilities, while NMN shows the most consistent benefits in terms of endurance-related performance.

These observations imply that the obvious superiority of a given NAD^+^ precursor cannot be generalized across domains, but must be explained in the context of the target results and the population studied. NAD^+^ precursor supplementation is unlikely to provide uniform benefits across populations, and may be most effective in individuals with underlying NAD^+^ deficiency, aging-related decline, or impaired metabolic function. Overall, these findings refute the concept of a universal precursor and underscore the importance of a precision supplementation model, where efficacy is determined by the specific type, dose, and characteristics of the target population.

### 4.5 Strengths, limitations, and future research directions

A key advantage of this study lies in its domain-based analytical framework, which recognizes fatigue as a multidimensional construct rather than a single homogeneous construct and systematically divides perceived fatigue, objective motor performance, cognitive outcomes, quality of life and sleep. This distinction is especially important in the context of NAD^+^ biology, because the basic mechanism is heterogeneous and the influence is unlikely to be uniform across the physiological system. Within the motor performance domain, endurance-related performance and neuromuscular strength were further analyzed separately because they represent distinct physiological constructs with different underlying mechanisms, an approach consistent with established frameworks of fatigability and exercise physiology.

However, several restrictions should be admitted. First of all, substantial heterogeneity across fields was observed, especially in perceptual fatigue and cognitive performance, which was driven by the use of diverse measurement tools and participant characteristics. Second, relatively few studies were available for some intervention nodes, particularly NAM, resulting in less precise network estimates and unstable treatment rankings. Third, the intervention time in most included trials is relatively short, and the characteristics of the populations vary widely, which may mask the impact of long-term or specific populations. Fourth, the evidence on sleep outcomes was insufficient to permit network meta-analysis. Finally, an additional methodological limitation relates to the geometry of the treatment networks. Most outcome networks were placebo-centered star networks with few direct head-to-head comparisons between active NAD^+^ precursors. Consequently, treatment rankings relied largely on indirect evidence. Although no global inconsistency was detected, the absence of closed loops warrants cautious interpretation of the comparative estimates until more head-to-head randomized controlled trials become available.

Future research should prioritize longer (>6 months), power-sufficient and domain-specific RCTs to better capture chronic adaptation of fatigue and functional performance. Given the consistent NMN signal observed across endurance-related outcomes, the design of the phenotype drive may further improve interpretability. At the same time, the integration of objective biomarkers including NAD^+^ bioavailability, mitochondrial function, redox balance, inflammatory state, skeletal muscle oxygen utilization and central fatigue-related markers, is crucial to clarify the biological mechanism underlying treatment responses.

Importantly, the huge inter-individual variability needs to turn to a precise approach, combining the baseline NAD^+^ state and quantifying ΔNAD^+^ to better define the optimal treatment window and dose response relationship. Combining objective functional results with mechanical biomarkers, including NAD^+^ bioavailability, mitochondrial function, redox balance and inflammatory characteristics, is crucial to clarify the potential pathway, especially the effect of skeletal muscle O_2_ utilization. In addition, direct head-to-head comparisons between different NAD^+^ precursors are needed to strengthen comparative effectiveness estimates and reduce reliance on indirect evidence. Future trials should also optimize dosage, intervention duration and timing strategies. Finally, evaluating NAD^+^ supplements alongside lifestyle interventions, particularly structured exercise and dietary modification, may provide a more clinically relevant framework for preventing fatigue and improving functional outcomes.

## 5 Conclusion

In conclusion, NAD^+^ precursor supplements have a selective, domain-specific and context-dependent effect on fatigue-related outcomes. The most consistent benefits were observed in endurance-related motor performance, especially at medium and high doses of NMN, while the effects on perceived fatigue, cognitive performance and sleep quality were generally limited or inconsistent. These findings support stratification, threshold, and responder-dependent models, suggesting that NAD^+^ supplementation primarily addresses the biological energy constraints underlying sustained physical performance, with only partial effects extending into more complex neuropsychological fields. Collectively, this underscores the need for precision-based, domain-targeted NAD^+^ supplementation strategies and for future trials that incorporate domain-specific endpoints and mechanistic biomarkers.

## Data Availability

The raw data supporting the conclusions of this article will be made available by the authors, without undue reservation.

## Glossary

ATP: Adenosine triphosphate
CI: Confidence interval
CN: Cognitively normal
FIS: Fatigue Impact Scale
FSS: Fatigue Severity Scale
FACIT-Fatigue: Functional Assessment of Chronic Illness Therapy–Fatigue
GRADE: Grading of Recommendations Assessment, Development and Evaluation
HVLT: Hopkins Verbal Learning Test
I^2^: I-squared statistic
ME/CFS: Myalgic Encephalomyelitis/Chronic Fatigue Syndrome
MoCA: Montreal Cognitive Assessment
NAM: Nicotinamide
NAD^+^: Nicotinamide adenine dinucleotide
NADH: Reduced nicotinamide adenine dinucleotide
NMA: Network meta-analysis
NMN: Nicotinamide mononucleotide
NR: Nicotinamide riboside
PICOS: Participants, Interventions, Comparisons, Outcomes, Study design
PRISMA: Preferred Reporting Items for Systematic Reviews and Meta-Analyses
PROSPERO: International Prospective Register of Systematic Reviews
PSQI: Pittsburgh Sleep Quality Index
QoL: Quality of life
RBANS: Repeatable Battery for the Assessment of Neuropsychological Status
RCT: Randomized controlled trial
RoB 2: Risk of Bias 2 tool
SF-36: Short Form Health Survey (36-item)
SIRT1: Sirtuin 1
Slc12a8: Solute carrier family 12 member 8
SMD: Standardized mean difference
SPPB: Short Physical Performance Battery
STS: Sit-to-stand test
SUCRA: Surface under the cumulative ranking curve
TMT-B: Trail Making Test Part B
VO₂max: Maximal oxygen uptake
VO₂peak: Peak oxygen uptake
WHO: World Health Organization
