# Supplementary material for: Domain-specific effects of NAD+ precursors on fatigue and functional performance: a systematic review and network meta-analysis
Source: Front Nutr. 2026 Sep 8;13:1890335. doi: 10.3389/fnut.2026.1890335 (PMC13593507; doi:10.3389/fnut.2026.1890335)
Supplement: Supplementary file 1 [file Data_Sheet_1.pdf]

Supplementary Material

--Domain-specific Effects of NAD$^+$ Precursors on Fatigue and Functional Performance: A Systematic Review and Network Meta-Analysis

Content

Appendix 1 Search strategy 2

Appendix 2 Study characteristics 7

Appendix 3 Risk of bias 14

Appendix 4 The analysis for outcomes 15

Appendix 5 GRADE Evidence Quality Rating for NMA 48

Appendix 6 PRISMA NMA Checklist 50

1 Appendix 1 Search strategy

Table S1. Search strategies

#	PubMed Searches	Results
1	"NAD"[MeSH Terms] OR "Nicotinamide Adenine Dinucleotide"[Title/Abstract] OR "NAD"[Title/Abstract] OR "NAD+"[Title/Abstract] OR NADH[Title/Abstract] OR "NAD supplement*"[Title/Abstract] OR "NAD precursor*"[Title/Abstract] OR "NAD booster*"[Title/Abstract] OR "Nicotinamide Riboside"[Title/Abstract] OR "nicotinamide riboside chloride"[Title/Abstract] OR NR[Title/Abstract] OR Niagen[Title/Abstract] OR "Nicotinamide Mononucleotide"[Title/Abstract] OR NMN[Title/Abstract] OR "beta-NMN"[Title/Abstract] OR "β-NMN"[Title/Abstract]	108,042
2	"Fatigue"[MeSH Terms] OR fatigue[tiab] OR state fatigue[tiab] OR tiredness[tiab] OR exhaustion[tiab] OR lethargy[tiab] OR vitality[tiab] OR "lack of energy"[tiab] OR energy[tiab] OR "FACIT-Fatigue"[tiab] OR "Chalder Fatigue"[tiab] OR FSS[tiab] OR MFI[tiab] OR "Piper Fatigue"[tiab] OR "Chronic Fatigue Syndrome"[tiab] OR "CFS/ME"[tiab] OR "Myalgic Encephalomyelitis"[tiab] OR "Long COVID"[tiab] OR "Post-COVID"[tiab] OR "Cognition"[MeSH Terms] OR cognition[tiab] OR "cognitive function*"[tiab] OR "cognitive decline"[tiab] OR "cognitive impairment"[tiab] OR memory[tiab] OR attention[tiab] OR "executive function*"[tiab] OR "processing speed"[tiab] OR MoCA[tiab] OR "Montreal Cognitive Assessment"[tiab] OR MMSE[tiab] OR "Mini-Mental"[tiab] OR AVLT[tiab] OR RAVLT[tiab] OR "Trail Making"[tiab] OR TMT[tiab] OR Stroop[tiab] OR "Digit Span"[tiab] OR "Physical Functional Performance"[MeSH Terms] OR "physical function*"[tiab] OR "physical performance"[tiab] OR "functional capacity"[tiab] OR "exercise capacity"[tiab] OR "mobility"[tiab] OR "endurance"[tiab] OR "muscle strength"[tiab] OR "grip strength"[tiab] OR handgrip[tiab] OR "6-minute walk"[tiab] OR "6-Minute Walk"[tiab] OR 6MWT[tiab] OR SPPB[tiab] OR "Short Physical Performance Battery"[tiab] OR "Timed Up and Go"[tiab] OR TUG[tiab] OR "chair stand"[tiab] OR "walking speed"[tiab] OR frailty[tiab] OR sarcopenia[tiab] OR "Activities of Daily Living"[MeSH Terms] OR ADL[tiab] OR IADL[tiab] OR sleep[tiab] OR "quality of life"[tiab]	3,537,177
3	randomized controlled trial[pt] OR controlled clinical trial[pt] OR "clinical trial"[pt] OR "clinical trials as topic"[MeSH:noexp] OR randomized[tiab] OR randomised[tiab] OR randomly[tiab] OR placebo[tiab] OR "placebo-controlled"[tiab] OR "double blind"[tiab] OR "single blind"[tiab] OR crossover[tiab] OR trial[ti]	2,073,852
4	1 AND 2 AND 3	534
5	4 NOT (animals[mh] NOT humans[mh])	429
**#**	**Web of Science Searches**	**Results**
1	TS = ("Nicotinamide Adenine Dinucleotide" OR NAD OR "NAD+" OR NADH OR "Reduced Nicotinamide Adenine Dinucleotide" OR "Nicotinamide Riboside" OR "Nicotinamide Ribosides" OR NR OR Niagen OR "Tru Niagen" OR NiaCel OR "NAD precursor*" OR "NAD booster*" OR "NAD supplement*" OR "Nicotinamide Mononucleotide" OR "Nicotinamide Mononucleotides" OR NMN)	72,391

2	TS = (Fatigue OR State fatigue OR Tiredness OR Exhaustion OR Lethargy OR "Lack of energy" OR Vitality OR "Chronic Fatigue Syndrome" OR "Myalgic Encephalomyelitis" OR CFS OR "Long COVID" OR "Post-COVID" OR "FACIT-Fatigue" OR "Chalder Fatigue" OR "Fatigue Severity Scale" OR FSS OR MFI OR "Piper Fatigue" OR Cognition OR "Cognitive function*" OR "Cognitive decline" OR "Cognitive impairment" OR Memory OR Attention OR "Executive function*" OR "Processing speed" OR MoCA OR "Montreal Cognitive Assessment" OR MMSE OR "Mini-Mental" OR AVLT OR RAVLT OR "Trail Making" OR TMT OR Stroop OR "Digit Span" OR "Physical function*" OR "Functional capacity" OR "Physical performance" OR "Exercise capacity" OR Endurance OR Mobility OR Frailty OR Sarcopenia OR "Muscle strength" OR "Grip strength" OR Handgrip OR "6-Minute Walk" OR "6 minute walk" OR 6MWT OR SPPB OR "Short Physical Performance Battery" OR "Timed Up and Go" OR TUG OR "Chair stand" OR "Walking speed" OR Sleep OR "Sleep quality" OR "Quality of life" OR ADL OR IADL)	3,109,765
3	TS = (Randomized OR Randomised OR Random* OR "Randomized Controlled Trial" OR "Controlled Clinical Trial" OR "Clinical Trial" OR Placebo OR "Placebo-controlled" OR Trial* OR RCT)	2,541,934
4	#1 AND #2 AND #3	517
5	#4 NOT TS = ("Nicotinic Acid*" OR Niacin OR Nicotinamide OR NAM OR "Vitamin B3" OR Tryptophan OR animal* OR mice OR rats OR mouse OR rodent* OR child* OR pediatric*)	333

#	**Ovid MEDLINE Search Strategy**	**Results**
1	exp Nicotinamide Adenine Dinucleotide/ OR NAD.tw. OR"NAD+".tw. OR NADH.tw.<br>OR "Nicotinamide Riboside".tw. OR NR.tw.<br>OR Niagen.tw. OR "Tru Niagen".tw.<br>OR NiaCel.tw. OR "Nicotinamide Mononucleotide".tw.<br>OR NMN.tw. OR "NAD precursor*".tw. OR "NAD booster*".tw. OR "NAD supplement*".tw.	127,147
2	exp Fatigue/<br>OR fatigue.tw. OR state fatigue.tw. OR tiredness.tw.<br>OR exhaustion.tw. OR lethargy.tw. OR vitality.tw.<br>OR "lack of energy".tw. OR exp Cognition/ OR cognition.tw.<br>OR "cognitive function*".tw. OR "cognitive decline".tw.<br>OR "executive function*".tw. OR MoCA.tw.<br>OR MMSE.tw. OR AVLT.tw.<br>OR RAVLT.tw. OR "physical functional performance".tw.<br>OR "physical performance".tw. OR "exercise capacity".tw.<br>OR "muscle strength".tw. OR "grip strength".tw.<br>OR 6MWT.tw. OR SPPB.tw. OR TUG.tw.<br>OR ADL.tw. OR IADL.tw. OR "quality of life".tw.	2,672,184
3	randomized controlled trial.pt.<br>OR controlled clinical trial.pt.<br>OR randomized.ab.<br>OR randomised.ab.<br>OR randomly.ab.<br>OR placebo.ab.<br>OR trial.ti.	5,257,042

4	#1 AND #2 AND #3	936
5	limit #4 to humans	887
#	**Embase.com Search Strategy**	**Results**
1	('nicotinamide adenine dinucleotide'/exp OR 'nad':ti,ab OR 'nad+':ti,ab OR 'nadh':ti,ab OR 'nicotinamide riboside':ti,ab OR nr:ti,ab OR niagen:ti,ab OR 'tru niagen':ti,ab OR niacel:ti,ab OR 'nicotinamide mononucleotide':ti,ab OR nmn:ti,ab OR 'nad precursor*':ti,ab OR 'nad booster*':ti,ab OR 'nad supplement*':ti,ab)	124,297
2	('fatigue'/exp OR fatigue:ti,ab OR state fatigue:ti,ab OR tiredness:ti,ab OR exhaustion:ti,ab OR lethargy:ti,ab OR vitality:ti,ab OR 'lack of energy':ti,ab OR 'cognition'/exp OR cognition:ti,ab OR 'cognitive function*':ti,ab OR 'cognitive impairment':ti,ab OR 'executive function*':ti,ab OR moca:ti,ab OR mmse:ti,ab OR 'physical performance':ti,ab OR 'exercise capacity':ti,ab OR 'muscle strength':ti,ab OR 'grip strength':ti,ab OR 6mwt:ti,ab OR sppb:ti,ab OR tug:ti,ab OR adl:ti,ab OR iadl:ti,ab)	4,413,021
3	('randomized controlled trial'/exp OR randomized:ti,ab OR randomised:ti,ab OR random*:ti,ab OR placebo*:ti,ab OR 'clinical trial'/exp OR trial:ti)	4,207,127
4	#1 AND #2 AND #3	1412
5	NOT ('animal'/exp NOT 'human'/exp)	1347
6	#1 AND #2 AND #3 AND #4 ([controlled clinical trial]/lim OR [randomized controlled trial]/lim)	451
#	**Scopus Search Strategy**	**Results**
1	TITLE-ABS-KEY("Nicotinamide Adenine Dinucleotide" OR NAD OR "NAD+" OR NADH OR "Nicotinamide Riboside" OR NR OR Niagen OR "Tru Niagen" OR NiaCel OR "Nicotinamide Mononucleotide" OR NMN OR "NAD precursor*" OR "NAD booster*" OR "NAD supplement*")	294,814
2	TITLE-ABS-KEY(Fatigue OR State fatigue OR Tiredness OR Exhaustion OR Lethargy OR "Lack of energy" OR Cognition OR "Cognitive function*" OR "Cognitive impairment" OR MoCA OR MMSE OR AVLT OR "Trail Making" OR Stroop OR "Physical performance" OR "Exercise capacity" OR "Muscle strength" OR "Grip strength" OR 6MWT OR SPPB OR TUG OR ADL OR IADL)	1,635,012
3	TITLE-ABS-KEY(Random* OR Randomized OR Randomised OR Placebo OR "Clinical Trial" OR RCT)	5,091,732
4	#1 AND #2 AND #3	1,535
5	NOT TITLE-ABS-KEY("Nicotinic Acid*" OR Niacin OR Nicotinamide OR NAM OR "Vitamin B3" OR Tryptophan OR animal* OR mice OR rats OR pediatric*)	653
#	**Cochrane CENTRAL Search Strategy**	**Results**

1	(NAD OR "NAD+" OR NADH OR "nicotinamide adenine dinucleotide"<br>OR "nicotinamide riboside" OR NR OR Niagen OR "Tru Niagen"<br>OR NiaCel OR "nicotinamide mononucleotide" OR NMN<br>OR "NAD precursor" OR "NAD precursors"<br>OR "NAD booster" OR "NAD boosters")	9166
2	(fatigue OR state fatigue OR tiredness OR exhaustion OR lethargy OR vitality OR cognition OR memory OR attention OR "executive function" OR "physical performance" OR "physical function"<br>OR "exercise capacity" OR "muscle strength" OR "grip strength" OR "walking speed" OR 6MWT OR SPPB OR TUG OR ADL OR IADL)	213,692
3	#1 AND #2	937

#	**PsycINFO (EBSCO) Search Strategy**	**Results**
1	TX("Nicotinamide Adenine Dinucleotide" OR "NAD+" OR NAD OR NADH OR "Nicotinamide Riboside" OR NR OR Niagen OR "Tru Niagen" OR "Nicotinamide Mononucleotide" OR NMN<br>OR "NAD precursor*" OR "NAD booster*" OR "NAD supplement*")	4,010
2	TX(Fatigue OR State fatigue OR Tiredness OR Exhaustion OR Lethargy OR "Lack of energy" OR Vitality OR "Chronic Fatigue Syndrome" OR CFS OR "Myalgic Encephalomyelitis"<br>OR "Long COVID" OR Post-COVID OR Cognition OR "Cognitive function*" OR "Cognitive impairment" OR "Cognitive decline" OR Memory OR Attention OR "Executive function*" OR "Processing speed" OR MoCA OR MMSE OR AVLT OR RAVLT<br>OR "Trail Making" OR TMT OR Stroop OR "Digit Span"<br>OR "Physical function*" OR "Physical performance"<br>OR "Exercise capacity" OR "Muscle strength" OR "Grip strength"<br>OR 6MWT OR SPPB OR TUG OR ADL OR IADL<br>OR "Quality of life" OR Sleep OR "Sleep quality")	1,314,911
3	TX(Randomized OR Randomised OR Random* OR Placebo<br>OR "Randomized Controlled Trial" OR "Clinical Trial" OR Trial* OR RCT)	559,099
4	S1 AND S2 AND S3	599

#	**ClinicalTrials.gov Search Strategy**	**Results**
5	Advance search → Condition / Other terms： ("NAD" OR "NAD+" OR "Nicotinamide Riboside" OR "Niagen" OR "Nicotinamide Mononucleotide" OR NMN) AND (Fatigue OR Cognition OR "Physical performance" OR "Exercise capacity" OR "Muscle strength") Study Type → Interventional Studies (Clinical Trials) Recruitment → ALL（or Active/Completed）	264

#	**WHO ICTRP Search Strategy**	**Results**
5	Search terms: (NAD OR "NAD+" OR "Nicotinamide Riboside" OR Niagen OR "Nicotinamide Mononucleotide" OR NMN) AND (Fatigue OR Cognition OR Exercise OR Muscle OR Performance)<br>Condition: Fatigue OR Cognition OR Exercise OR Muscle OR Performance<br>Filters Applied: Recruitment status: ALL; Study type: Interventional	351

#	PROQUEST Search Strategy	Results
1	NOFT("Nicotinamide Adenine Dinucleotide" OR NAD OR "NAD+" OR NADH OR "Nicotinamide Riboside" OR "Nicotinamide Ribosides"OR NR OR Niagen OR "Tru Niagen" OR NiaCel OR "Nicotinamide Mononucleotide" OR NMN OR "NAD precursor" OR "NAD precursors" OR "NAD booster" OR "NAD boosters" OR "NAD supplement" OR "NAD supplements")	26,228
2	NOFT(Fatigue OR State Fatigue OR Tiredness OR Exhaustion OR Lethargy OR "Lack of energy" OR Vitality OR "Chronic Fatigue Syndrome" OR CFS OR "Myalgic Encephalomyelitis" OR "Long COVID" OR Post-COVID OR "FACIT Fatigue" OR Chalder OR "Fatigue Severity Scale" OR FSS OR MFI OR Cognition OR "Cognitive function" OR "Cognitive impairment" OR "Cognitive decline" OR Memory OR Attention OR "Executive function" OR "Processing speed" OR MoCA OR MMSE OR AVLT OR RAVLT OR "Trail Making" OR TMT OR Stroop OR "Digit Span" OR "Physical function" OR "Physical performance" OR "Exercise capacity" OR "Muscle strength" OR "Grip strength" OR 6MWT OR SPPB OR TUG OR ADL OR IADL OR "Quality of life" OR Sleep OR "Sleep quality")	9,174,993
3	NOFT(Randomized OR Randomised OR Random* OR Placebo OR "Randomized Controlled Trial" OR "Controlled Clinical Trial" OR Trial* OR RCT)	8,051,415
4	#1 AND #2 AND #3 NOT (Newspapers AND Wire Feeds AND Blogs, Podcasts, & Websites AND Books)	1305

2 Appendix 2 Study characteristics

Table S2. Characteristics of the Studies Included in the Meta-analysis

	Study, Year	Research object (treatment vs control) group				Exercise intervention				Main Outcomes
		Dieases Type	Age	Sample Size/n	Female/Male	Intervention Method	NAD Precursor Dosage（mg/day）	Intake Frequency（times/day）	Duration/weeks	Outcomes
**1**	**Forsyth, L. M., 1999**	**CFS** (Chronic fatigue syndrome)	39.6 (26–57)	26	17/9	NADH（ENADA™）	10	1	4	Fatigue Symptom Scoring System
				26		Placebo				
**2**	**Birkmayer, G. D., 2002**	**Health Adult**	35-55	18	19/17	NADH（ENADA™）	20	once	NA	Stanford Sleepiness Scale; Tracking Falures（%）; Visual Comparison of Sequence: Correct Responses（min）
				17		Placebo				
**3**	**Demarin, V., 2004**	**AD**(Alzheimer's Disease)	68.5(52-79)	12	NA	NADH（ENADA™）	10	1	24	Mattis Dementia Rating Scale Total Score（MDRS）
				12		Placebo				

**4**	**Santaella, M. L., 2004**	**CFS**	31±9	12	18/2	NADH （ENADA™）	10	1	12	Fatigue Symptoms Score
				8		nutritional & psychological therapy				
**5**	**Mero, A., 2008**	**Healthy Male**	24.9 ± 2.8	8	0/8	NADH (ENACHI)	30	1 (10 mg×3)	4	Feelings of fatigue (1-7);<br>VO_2max (ml·kg^{-1}·min^{-1});<br>CMJ resting (cm)
				8		Placebo				
**6**	**Alegre, J.,2010**	**CFS<br>(Chronic fatigue syndrome)**	47（18-65）	36	72/5	NADH (Vitanadh®)	20	1×/day （5 mg×4）	8	Fatigue impact (FIS total); PSQI
				41		Placebo				
**7**	**Martens, C. R.,2018**	**Healthy middle-aged and older men and<br>women**	T：66 ± 9	12	6/6	NR （NIAGEN®）	1000	2×/day （500 mg×2）	6	VO_2max (ml·kg^{-1}·min^{-1});<br>Hand-grip (kg);<br>6MWT (m);<br>5min-sit up (s)
			C：64 ± 6	12	5/7	Placebo				
**8**	**Elhassan, Y. S.,2019**	**Healthy Old Male**	75 (72-78)	12	0/12	NR （Niagen®）	1000	2×/day （250 mg×4）	3	Peak absolute Hand-grip strength (kg); Relative Hand-grip strength (kg kg^{-1})
				12		Placebo				

**9**	**Dolopikou, C. F.,2020**	**Healthy Young and Old Male**	T：Old:71.5±1.0 Young: 22.9±1.0	12+12	0/12	NR (Life Extension®)	500	once	NA	Fatigue index; Isometric peak torque (N·m); Concentric peak torque (N·m); VO_{2max} (ml/kg/min)
			C：Old:71.5±1.0 Young: 22.9±1.0	12+12	0/12	Placebo (lactose capsules)				
**10**	**Ahmadi, A.,2023**	**Chronic Kidney Disease （CKD）**	61.0 ± 11.6	25	15/10	NR (NIAGEN®)	1000	1×/day	6	VO_{2peak} (mL/kg/min); Total work (kJ); Test Duration (seconds)
						Placebo				
**11**	**Brakedal, B.,2022**	**Parkinson's disease (PD)**	T:63.9 ± 8.0	15	4/11	NR (NIAGEN®)	1000	2×/day （500 mg×2）	4	MDS-UPDRS Total Score I-III; MDS-UPDRS Part I Global Cognitive Function; MDS-UPDRS Part III
			C:63.7 ± 11.0	15	1/14	Placebo (ChromaDex, USA.)				
**12**	**Berven,H.,2023**	**Parkinson's disease (PD)**	T:61.4±9.3	10	2/8	NR (NIAGEN®)	3000	2×/day （1500 mg × 2）	4	MDS-UPDRS Total Score I-III; MDS-UPDRS Part I Global Cognitive Function; MDS-UPDRS Part III
			C:65.5±9.0	10	3/7	Placebo				
**13**	**McDermott, M.**	**Peripheral artery disease**	T:73.21±9.97	28	12/16	NR	1000	2×/day	12/24	6MWT (m)

	**M.,2024**	**(PAD)**						（250 mg×4）		
			C:69.72±8.29	29	16/13	Placebo				
**14**	**Orr, M. E.,2024**	**Older adults with mild cognitive impairment (MCI)**	T:77.1 (71, 83)	10	5/5	NR (NIAGEN®)	1000	2×/day （250 mg×4）	10	CLOX (executive clock drawing task); CLOX2; EXIT executive interview; SPPB total score
			C:75.2 (67, 86)	10	8/2	Placebo				
**15**	**Wu, C. Y.,2025-AD**	**Older adults with subjective cognitive decline(SCD)or mild cognitive impairment (MCI)**	NR-PBO: 68.8 ±6.6	21	11/10	NR (TruNiagen)	1000	2×/day （250 mg×4）	4	RBANS total (Repeatable Battery for the Assessment of Neuropsychological Status); Lumosity gameplay score
			PBO-NR: 65.2± 7.8	20	11/9	Placebo				
**16**	**Wu Chao-Yi,2025-EB**	**Long-COVID**	NR-NR: 48.5 ± 13.0	29	24/5	NR （ChromaDex, Inc）	2000	2×/day （250mg×4×2）	10	Fatigue Severity Scale: (FSS); RBANS; Everyday Cognition scale (ECog); Trail Making Test-B (TMT-B); Pittsburgh Sleep Quality Index (PSQI)
			PBO-NR: 41.7 ± 12.33	21	12/9	Placebo				

**17**	**NCT03482167**	**Older Adults With Mild Cognitive Impairment （MCI）**	T:72±8	24	13/11	NR (NIAGEN®)	1000	2×/day （250 mg×4）	12	CVLT3 Total Recall Index Score；NIH Toolbox Fluid Cognition Score
			C:71±7	25	18/7	Placebo				
**18**	**NCT04078178**	**Subjective Cognitive Decline (SCD) and Mild Cognitive Impairment (MCI)**	≥65(58.7%) 18-65(41.3%)	23	13/10	NR （Niagen®）	1000	2×/day （250 mg×4）	8	RBANS
				23	13/10	Placebo				
**19**	**Igarashi, M. 2022**	**Healthy Old Male**	T：71.1 ± 3.9	10	0/42	NMN	250	once	12	Grip strength (kg)
			C：71.8 ± 6.1	10		Placebo				
**20**	**Huang, H.,2022**	**Health Adult**	T:47.76 ± 6.6	31	18/13	NMN （Uthever）	300	1×/day （150 mg×2）	4/8	SF-36; 6MWT (m)
			C:47.21± 6.55	31	16/15	Placebo (starch powder)				
**21**	**Kim, M.,2022**	**Healthy Old Males**	T:72.8±4.3	27	18/9	NMN (Life Sciences Ltd.,)	250	1×/day	12	Total fatigue score (point); Drowsiness (point) Total PSQI global score

			C:73.0 ±4.7	27	21/6	Placebo				(point); Daytime dysfunction score (point)<br>Grip strength (kg); 5-times sit to stand (s)
**22**	**Akasaka, H.,2023**	**Diabates Type 2 diabetes**	T:83.0 ± 6.7	7	0/14	NMN (TEIJIN Ltd.)	250	1×/day<br>(125 mg×2)	24	Grip strength (kg);<br>Five chair-standing time (s)
			C:79.3 ± 6.0	7		Placebo				
**23**	**Yi, Lin,2023**	**Healthy Middle-aged Adults**	T:<br>300mg:51.2 ± 7.0<br>600mg:49.5 ± 6.7<br>900mg:49.9 ± 6.3	20/20/20	10/10<br>14/6<br>11/9	NMN (AbinoNutra™)	300mg<br>600mg<br>900mg	1×/day<br>(150 mg×2/<br>4/6)	4/8	SF-36; 6WMT (m)
			C:46.5 ± 6.7	20	12/8	Placebo				
**24**	**Morifuji, M.,2024**	**Healthy Old Adults**	T:69 ± 3	30	12/18	NMN	250 mg/day	1×/day<br>(125 mg×2)	12	Fatigue-Inertia(FI);<br>Daytime dysfunction score (point); Selective response time; Reaction time; Grip strength(Dominant hand); SPPB-Total Score;
			C:69 ± 3	30	12/18	Placebo				

										Chair stand test;PSQI Total Global Score
**25**	**Kuerec, A. H.,2024**	**Healthy Middle-aged Adults**	T:<br>300mg:50(45,58.3)<br>600mg:48.5(44.8,53.3)<br>900mg: 49(44.8,53.3)	20/20/20	10/10<br>14/6<br>11/9	NMN	300mg<br>600mg<br>900mg	1×/day<br>(150 mg×2/<br>4/6)	4/8	6MWT; SF-36
			C: 44 (41, 49.3)	20	12/8	Placebo				
**26**	**Grill, Joshua D.,2025**	**Mild Cognitive Impairment (MCI) or Early Alzheimer Disease(AD)**	T: 74.6±9.0	24	10/14	NAM (Innovite, Inc.,),	3000mg/Day	2×/day<br>（750 mg×4）	48	CDR-SB;ADAS-cog;ADCS-ADL-MCI
			C: 72.9±7.4	22	10/12	Placebo				
**27**	**Schreiber, S.,2025**	**COVID-19**	T: 38 (18–75)	248	147/101	NAM	1000	1×/day<br>（500 mg×2）	4	FACIT-F; SF-36
			C: 39 (18–70)	252	144/108	Placebo				

3 Appendix 3 Risk of bias

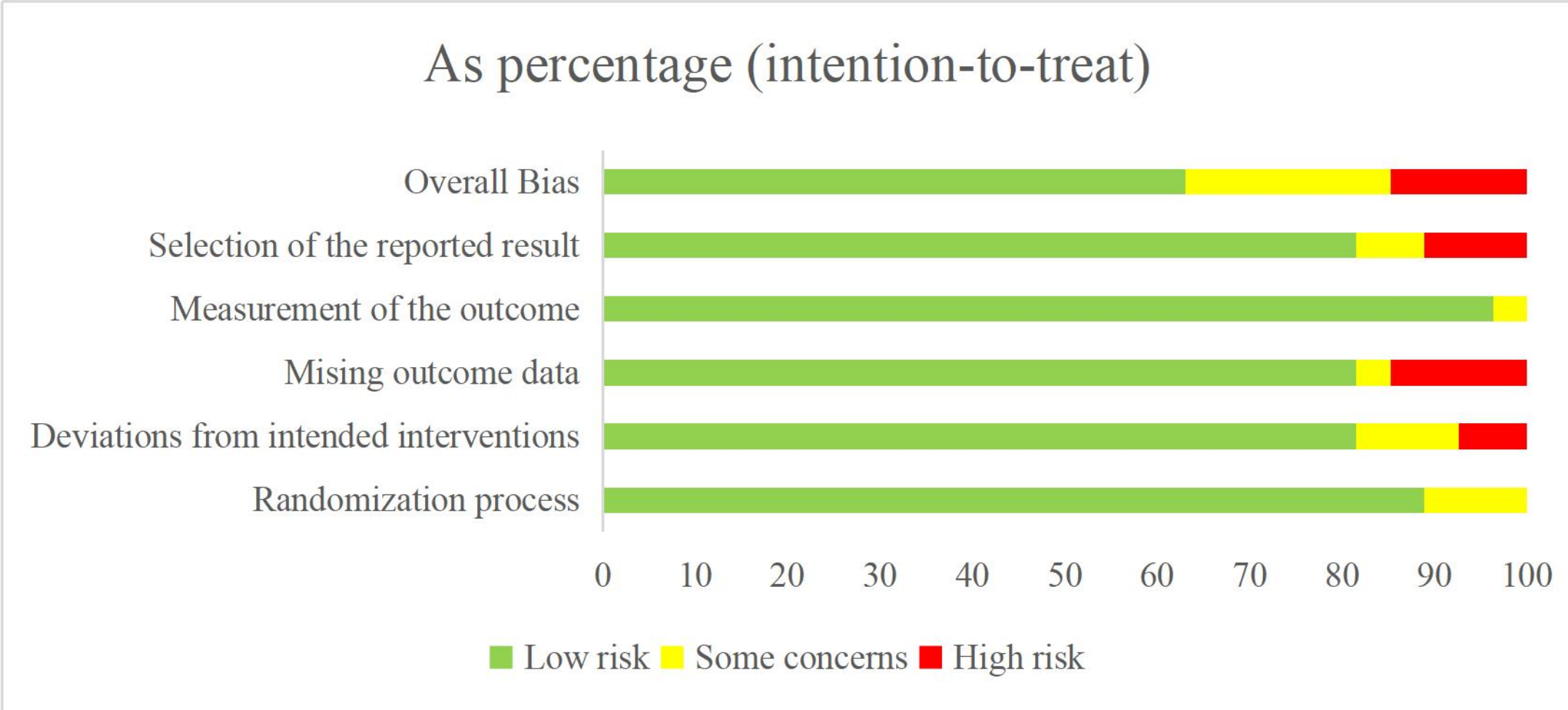

Figure S1 The overall risk of bias for all included studies

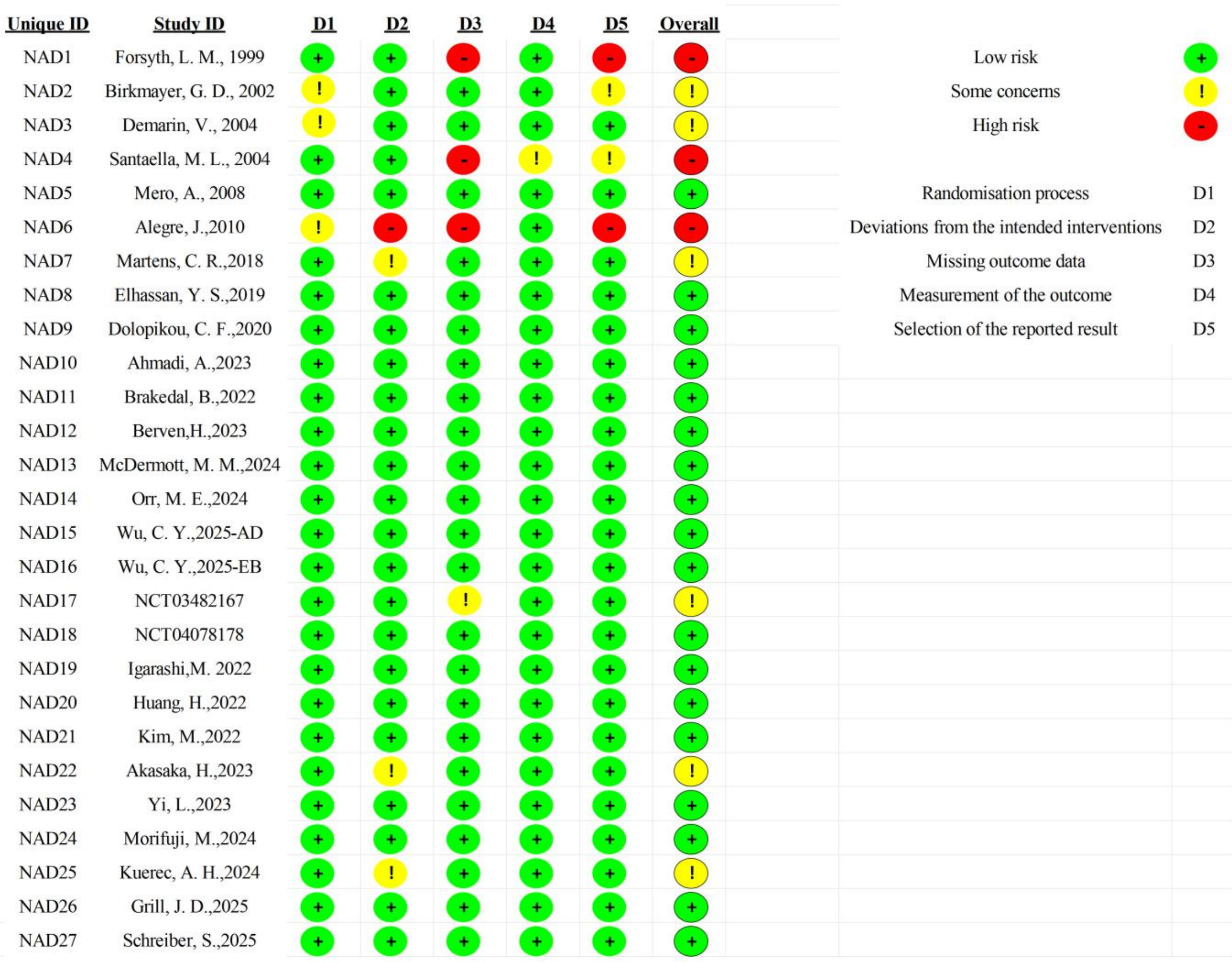

Unique ID	Study ID	D1	D2	D3	D4	D5	Overall
NAD1	Forsyth, L. M., 1999	+	+	-	+	-	-
NAD2	Birkmayer, G. D., 2002	!	+	+	+	!	!
NAD3	Demarin, V., 2004	!	+	+	+	+	!
NAD4	Santaella, M. L., 2004	+	+	-	!	!	-
NAD5	Mero, A., 2008	+	+	+	+	+	+
NAD6	Alegre, J.,2010	!	-	-	+	-	-
NAD7	Martens, C. R.,2018	+	!	+	+	+	!
NAD8	Elhassan, Y. S.,2019	+	+	+	+	+	+
NAD9	Dolopikou, C. F.,2020	+	+	+	+	+	+
NAD10	Ahmadi, A.,2023	+	+	+	+	+	+
NAD11	Brakedal, B.,2022	+	+	+	+	+	+
NAD12	Berven,H.,2023	+	+	+	+	+	+
NAD13	McDermott, M. M.,2024	+	+	+	+	+	+
NAD14	Orr, M. E.,2024	+	+	+	+	+	+
NAD15	Wu, C. Y.,2025-AD	+	+	+	+	+	+
NAD16	Wu, C. Y.,2025-EB	+	+	+	+	+	+
NAD17	NCT03482167	+	+	!	+	+	!
NAD18	NCT04078178	+	+	+	+	+	+
NAD19	Igarashi,M. 2022	+	+	+	+	+	+
NAD20	Huang, H.,2022	+	+	+	+	+	+
NAD21	Kim, M.,2022	+	+	+	+	+	+
NAD22	Akasaka, H.,2023	+	!	+	+	+	!
NAD23	Yi, L.,2023	+	+	+	+	+	+
NAD24	Morifuji, M.,2024	+	+	+	+	+	+
NAD25	Kuerec, A. H.,2024	+	!	+	+	+	!
NAD26	Grill, J. D.,2025	+	+	+	+	+	+
NAD27	Schreiber, S.,2025	+	+	+	+	+	+

Low risk	+
Some concerns	!
High risk	-
Randomisation process	D1
Deviations from the intended interventions	D2
Missing outcome data	D3
Measurement of the outcome	D4
Selection of the reported result	D5

Figure S2 The risk of bias for each study

4 Appendix 4 The analysis for outcomes

4.1 Effects of NAD+ on Perceived Fatigue

4.1.1 pairwise analysis for Perceived Fatigue

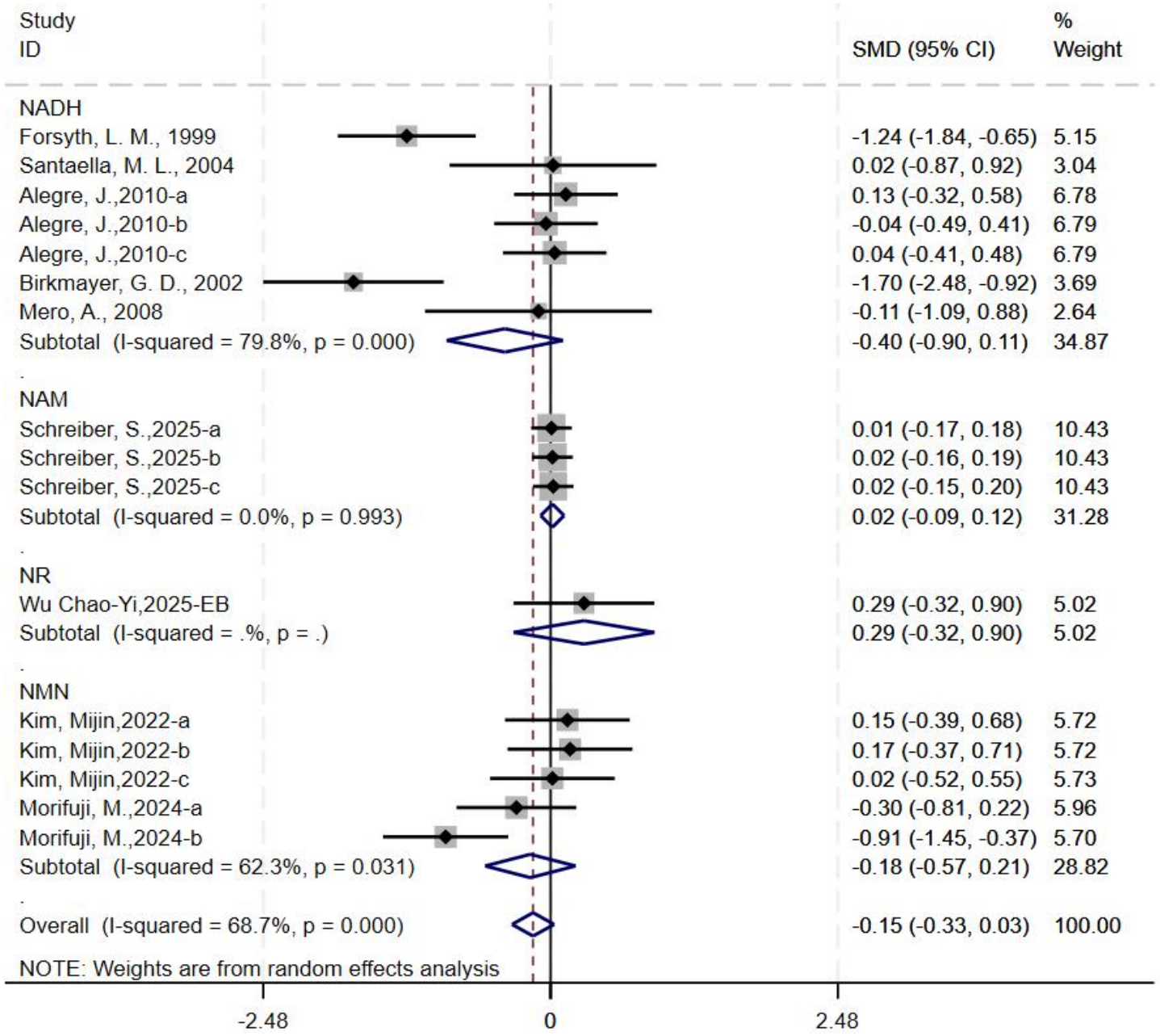

Figure S3 Forest plot of pairwise meta-analysis for perceived fatigue

Note. NADH means Nicotinamide Adenine Dinucleotide; NAM means Nicotinamide; NR means Nicotinamide Riboside; NMN means Nicotinamide Mononucleotide.

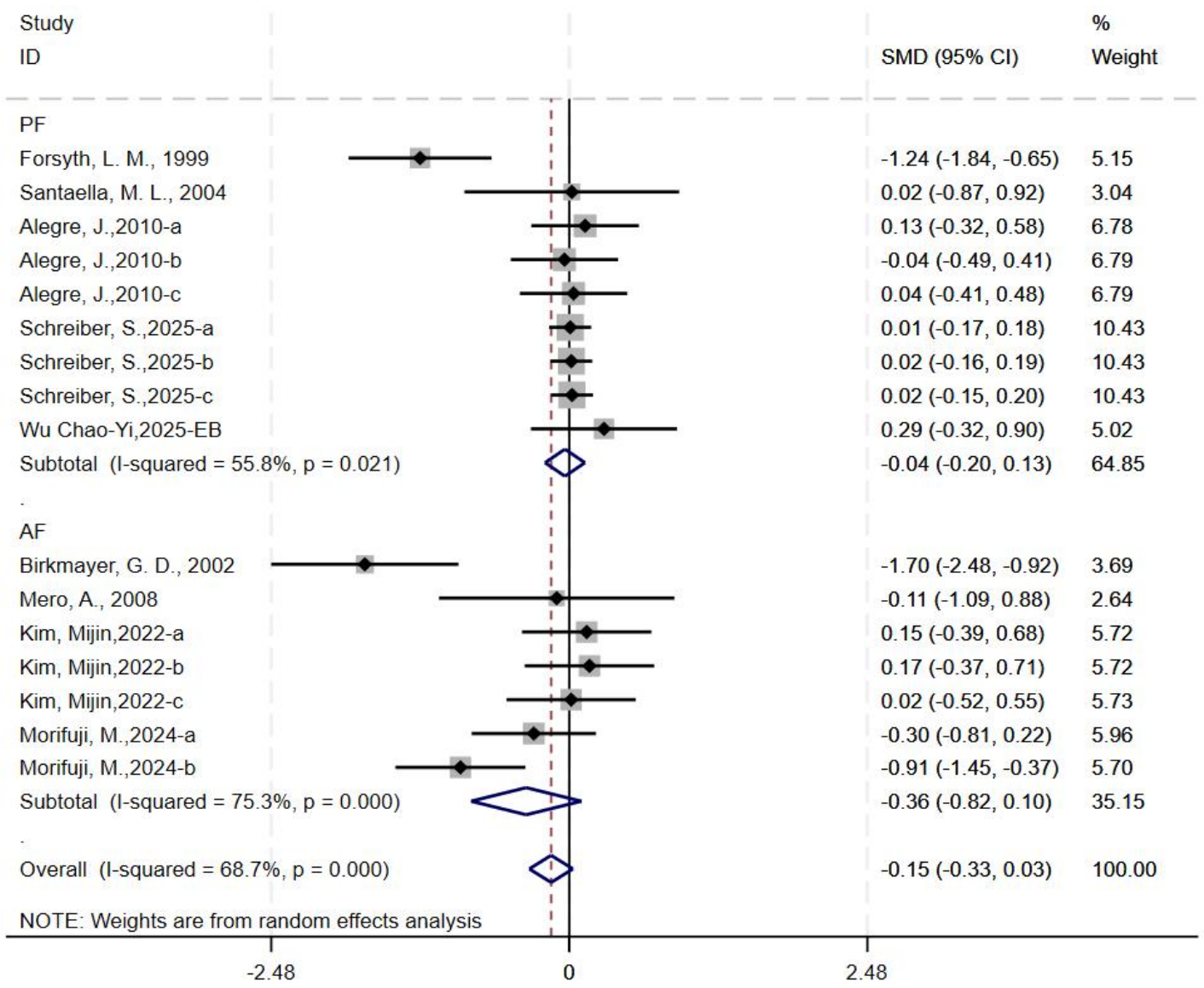

Figure S4 Forest plot of pairwise meta-analysis for perceived fatigue in PF & AF group.

4.1.2 Network analysis for Perceived Fatigue

Global inconsistency test

$$\chi^2 = 0.00$$

$$p = 0.9574$$

No evidence for the existence of significant global inconsistency.

The comparisons for different types of NAD^+ precursors and placebo control group

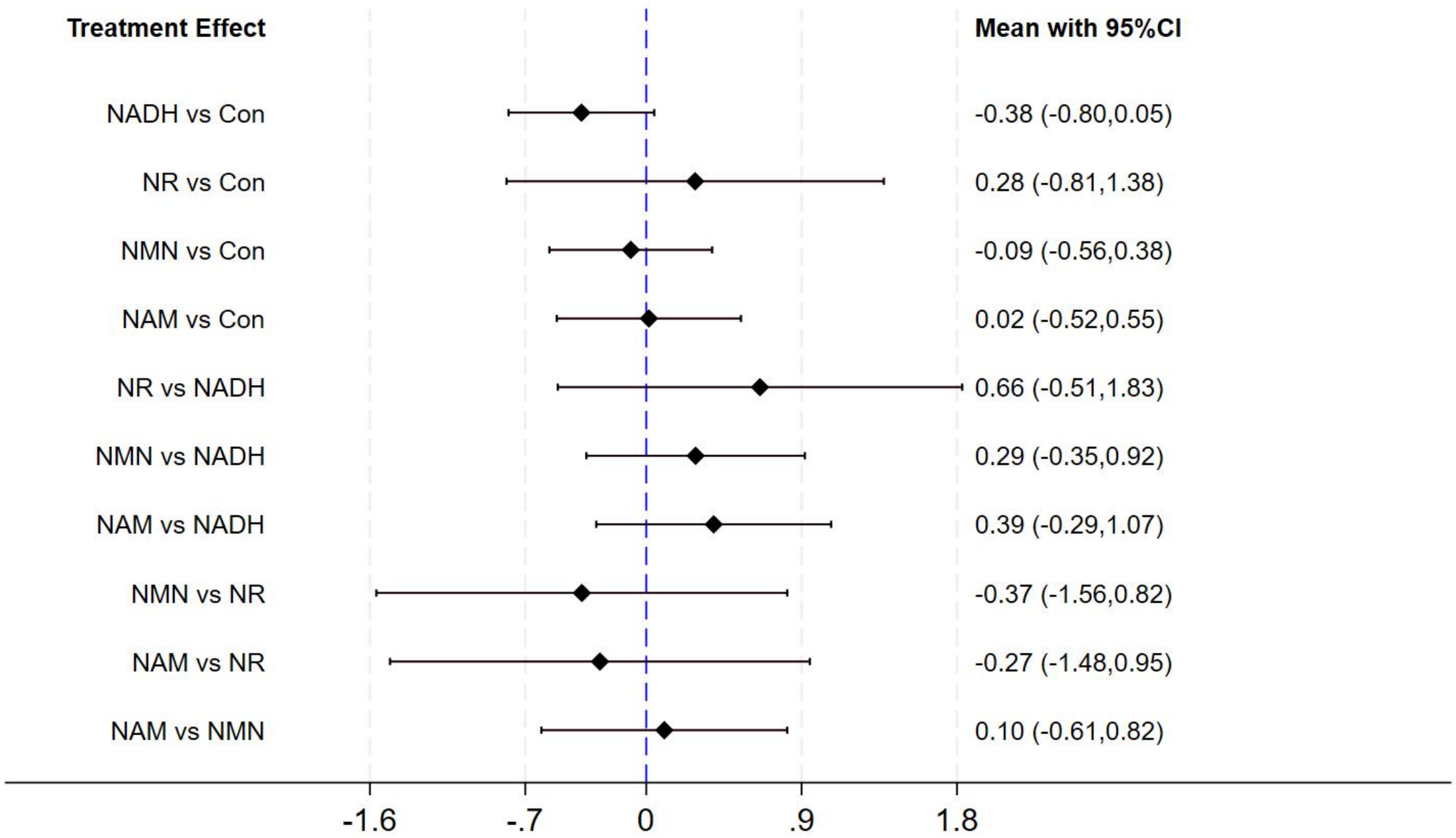

Figure S5 Interval plot of network meta-analysis for perceived fatigue

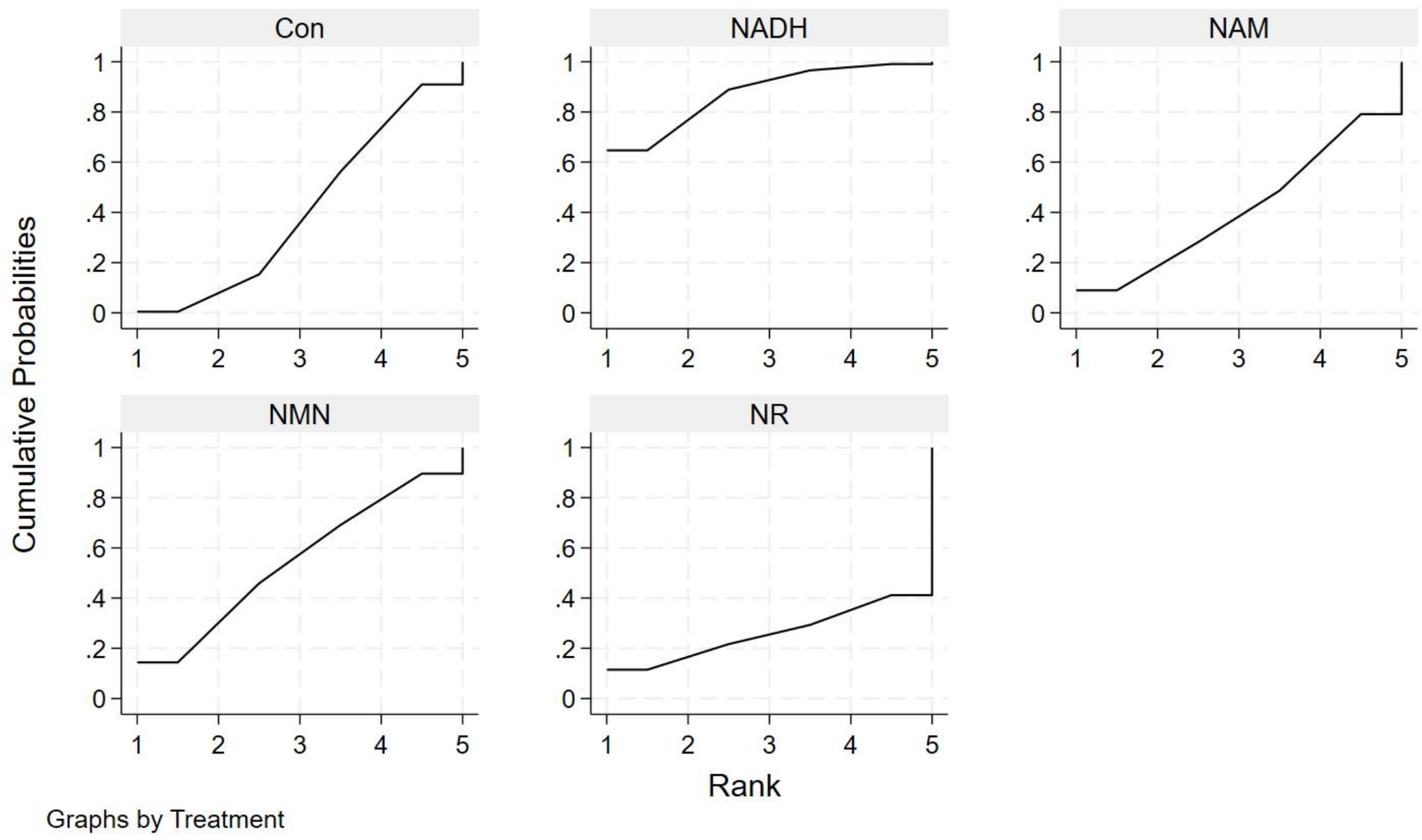

Figure S6 Cumulative ranking plot for perceived fatigue

Table S3. The ranking of effects of different types of NAD^+ precursors on perceived fatigue

**Treatment**	**Perceived Fatigue**		
	**SUCRA**	**Mean Rank**	**Probability of Best (%)**
Control	40.7	3.4	0.4
NADH	87.3	1.5	64.7
NR	25.9	4.0	11.5
NMN	54.8	2.8	14.4
NAM	41.2	3.4	9.0

4.1.3 Subgroup analysis for Perceived Fatigue in PF group

Global inconsistency test

$$\chi^2 = 0.01$$

$$p = 0.9374$$

No evidence for the existence of significant global inconsistency

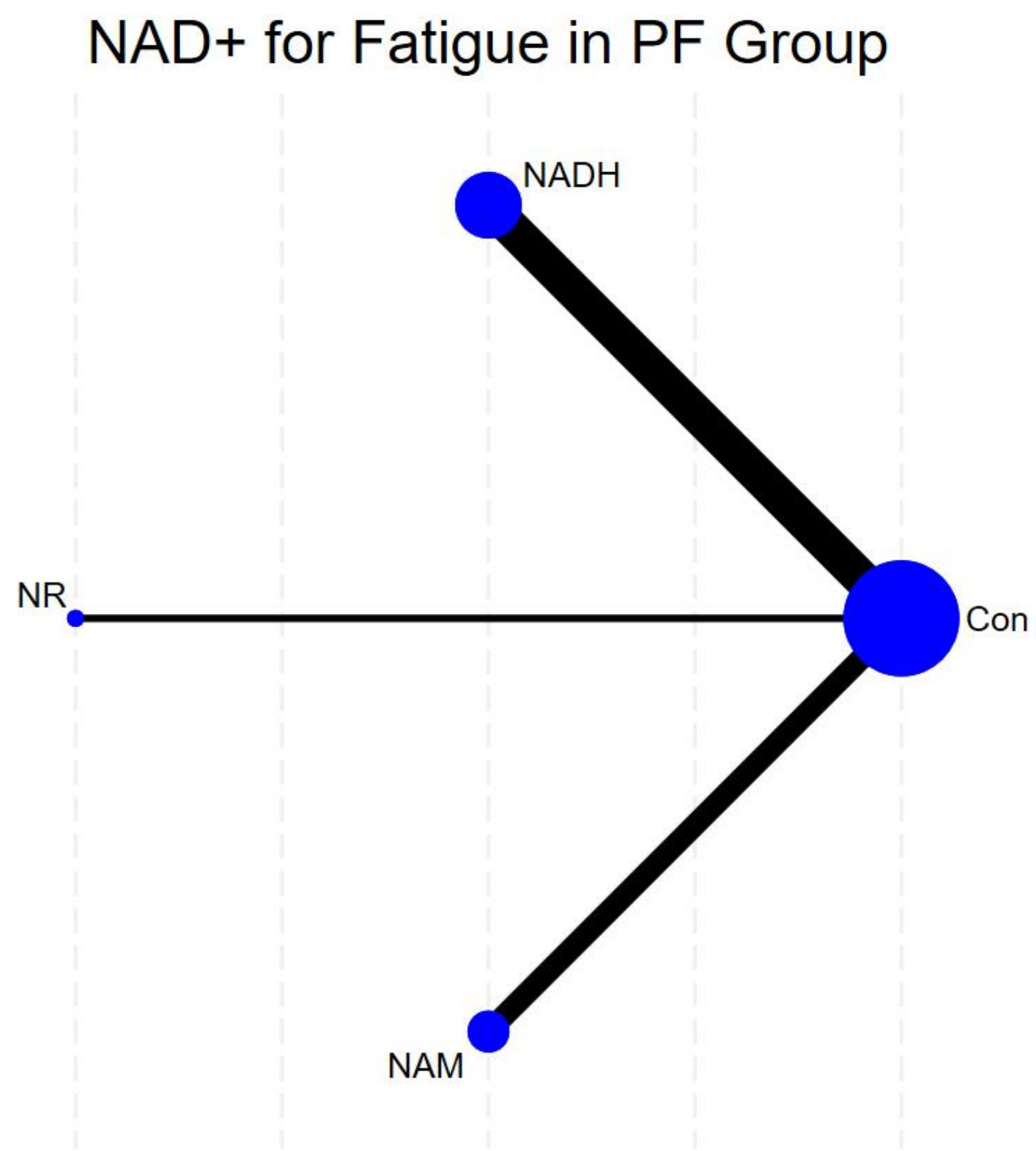

Figure S7 Network of eligible comparisons for perceived fatigue in PF group

The comparisons for different types of NAD^+ precursors and placebo control in PF group

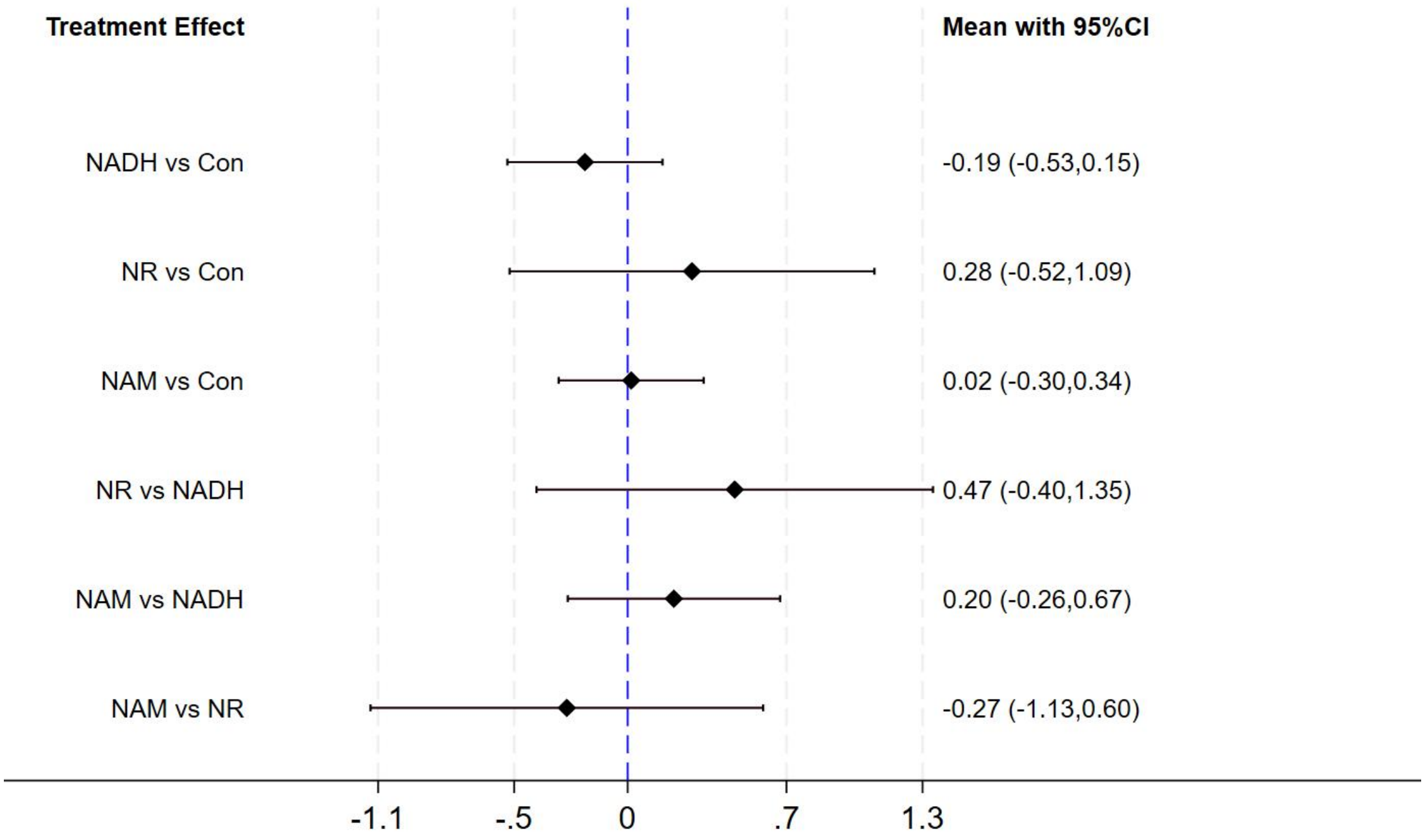

Figure S8 Interval plot of network meta-analysis for perceived fatigue in PF group

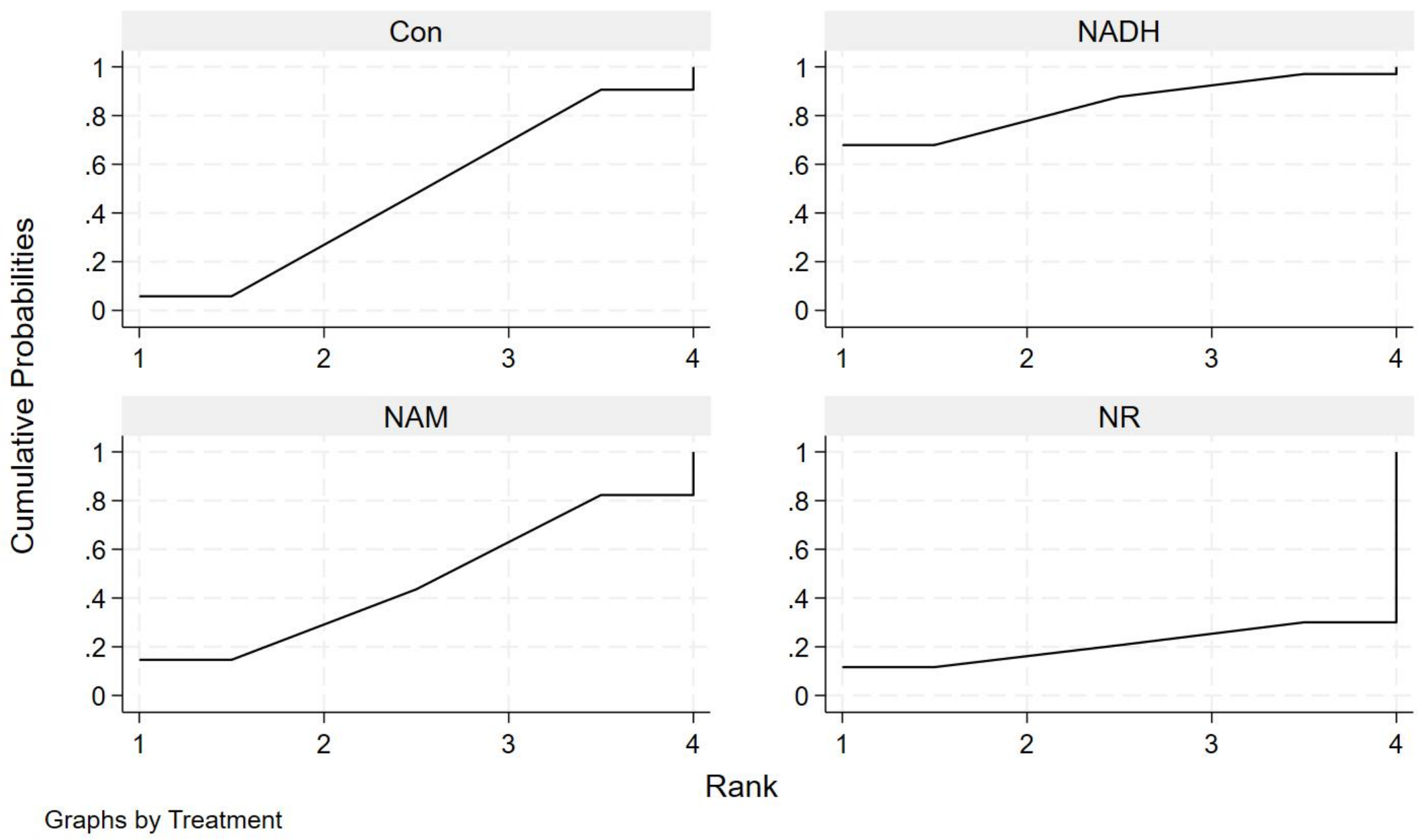

Figure S9 Cumulative ranking plot for perceived fatigue in PF group

Table S4. The ranking of effects of different types of NAD^+ precursors on perceived fatigue in PF group

Treatment	Perceived Fatigue		
	SUCRA	Mean Rank	Probability of Best (%)
Control	48.1	5.8	2.6
NADH	84.2	67.9	1.5
NR	20.8	11.7	3.4
NAM	46.9	14.6	2.6

4.1.4 Subgroup analysis for Perceived Fatigue in AF group

Global inconsistency test

$$\chi^2 = 0.10$$

$$p = 0.7653$$

No evidence for the existence of significant global inconsistency

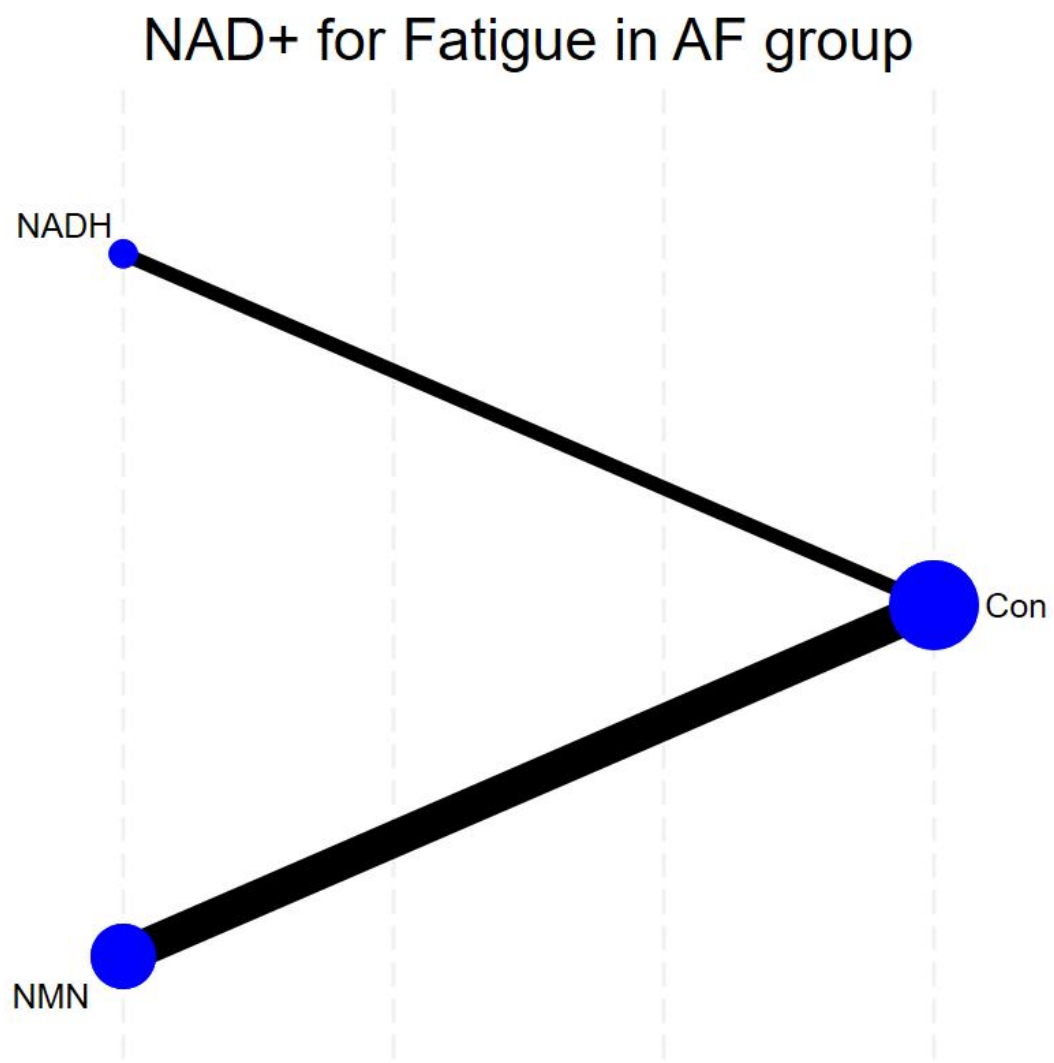

Figure S10 Network of eligible comparisons for perceived fatigue in AF group

The comparisons for different types of NAD^+ precursors and placebo control in AF group.

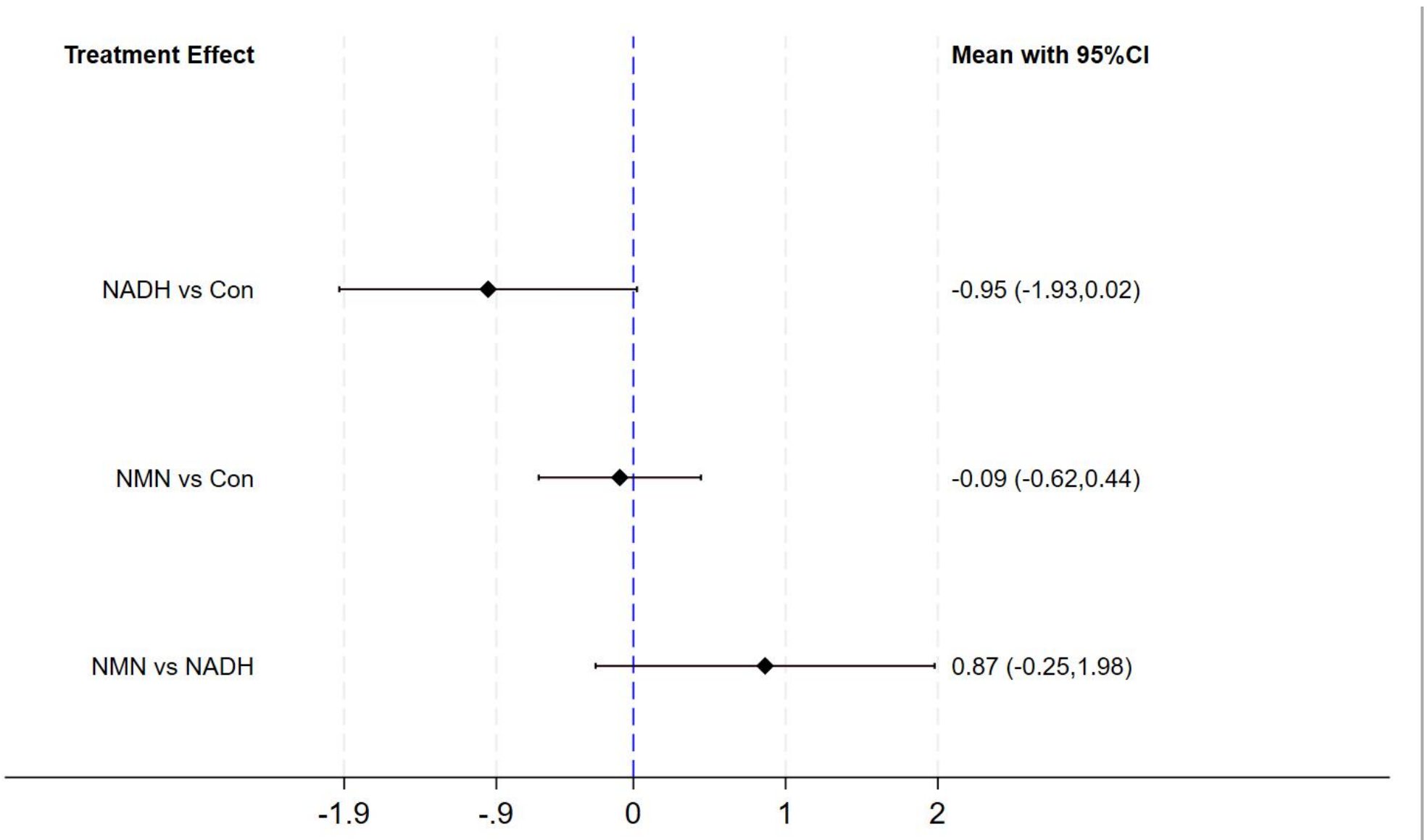

Figure S11 Interval plot of network meta-analysis for perceived fatigue in AF group

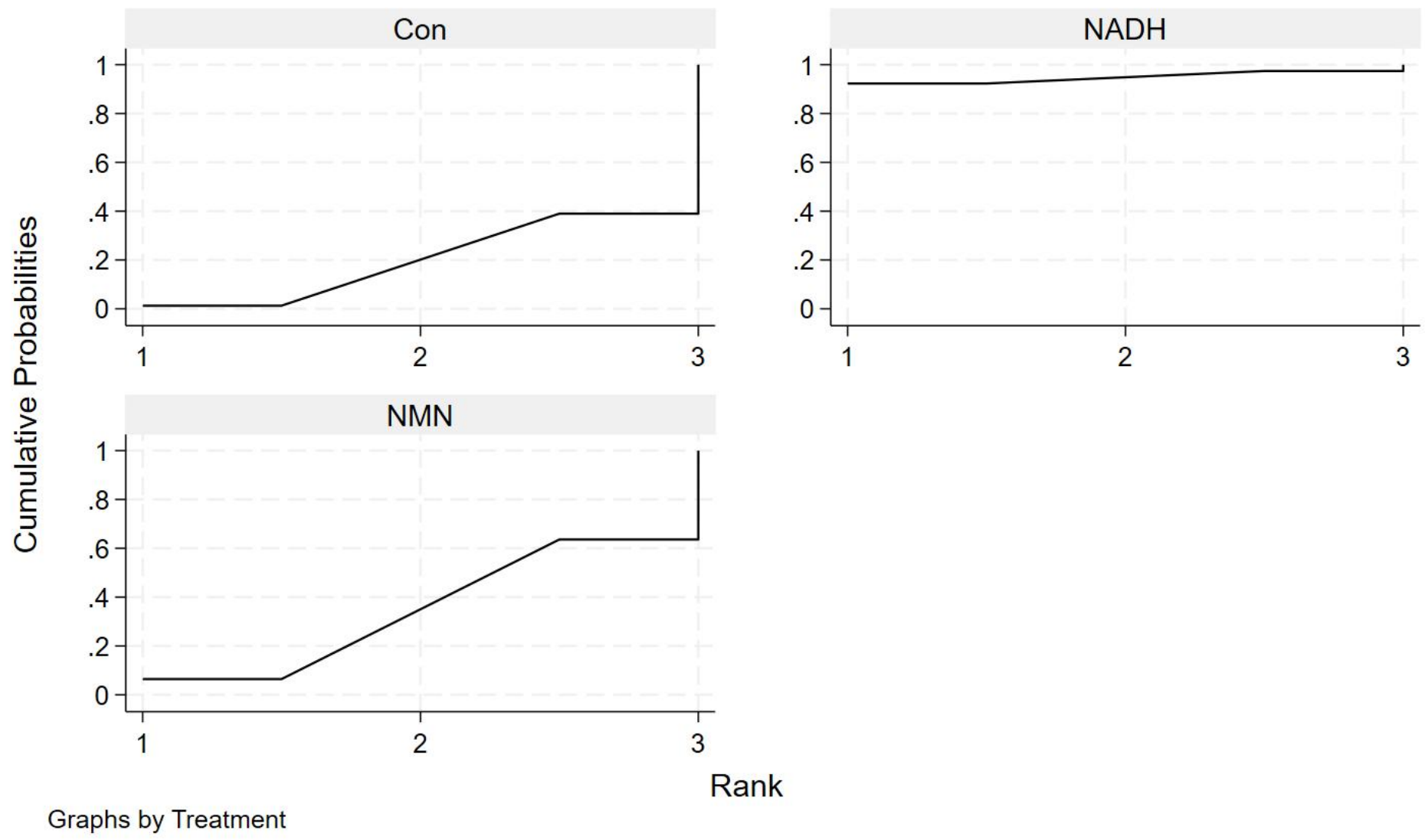

Figure S12 Cumulative ranking plot for perceived fatigue in AF group

Table S5. The ranking of effects of different types of NAD+ precursors on perceived fatigue in AF group

Treatment	Perceived Fatigue		
	SUCRA	Mean Rank	Probability of Best (%)
Control	20.1	2.6	1.3
NADH	94.8	1.1	92.3
NAM	35.0	2.3	6.4

4.1.5 Sensitivity analysis for Perceived Fatigue

Table S6. Sensitivity Analysis for Perceived Fatigue in the Network Meta-Analysis

Sensitivity Scenario	NADH vs. Control (SMD [95% CI])	SUCRA Ranking Order (Best to Worst)	Network I^2 (%)	τ^2	Overall Pairwise I^2 (%)
Excluding Forsyth (1999)	-0.21 [-0.62, 0.19]	NADH > NMN > Control > NAM > NR	64.72%	0.214	56.5%
Excluding Birkmayer (2002)	-0.19 [-0.56, 0.18]	NADH > NMN > NAM > Control > NR	62.45%	0.117	54.0%
Excluding Morifuji (2024-b)	-0.38 [-0.82, 0.06]	NADH > NMN > NAM > Control > NR	73.83%	0.241	63.2%
Excluding all NADH studies	N/A*	NMN > Control > NAM > NR	57.92%		

4.1.6 Publication Bias for Perceived Fatigue

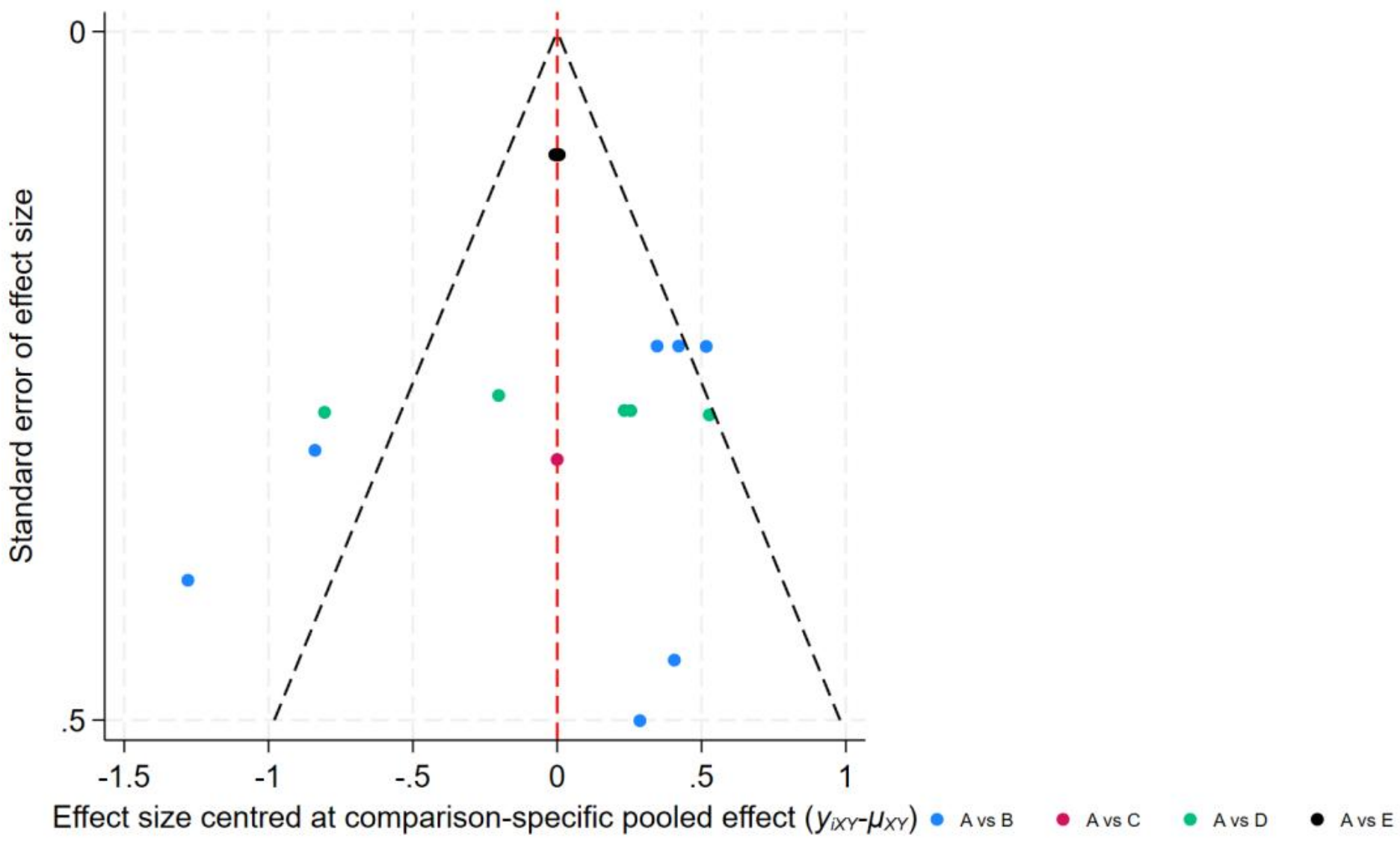

Figure S13 Funnel Plot for perceived fatigue

Note. A means placebo control; B means NADH; C means NR; D means NAM

4.2 Effects on Motor performance fatigue--Aerobic Capacity and Endurance

4.2.1 Pairwise analysis for Aerobic Capacity and Endurance

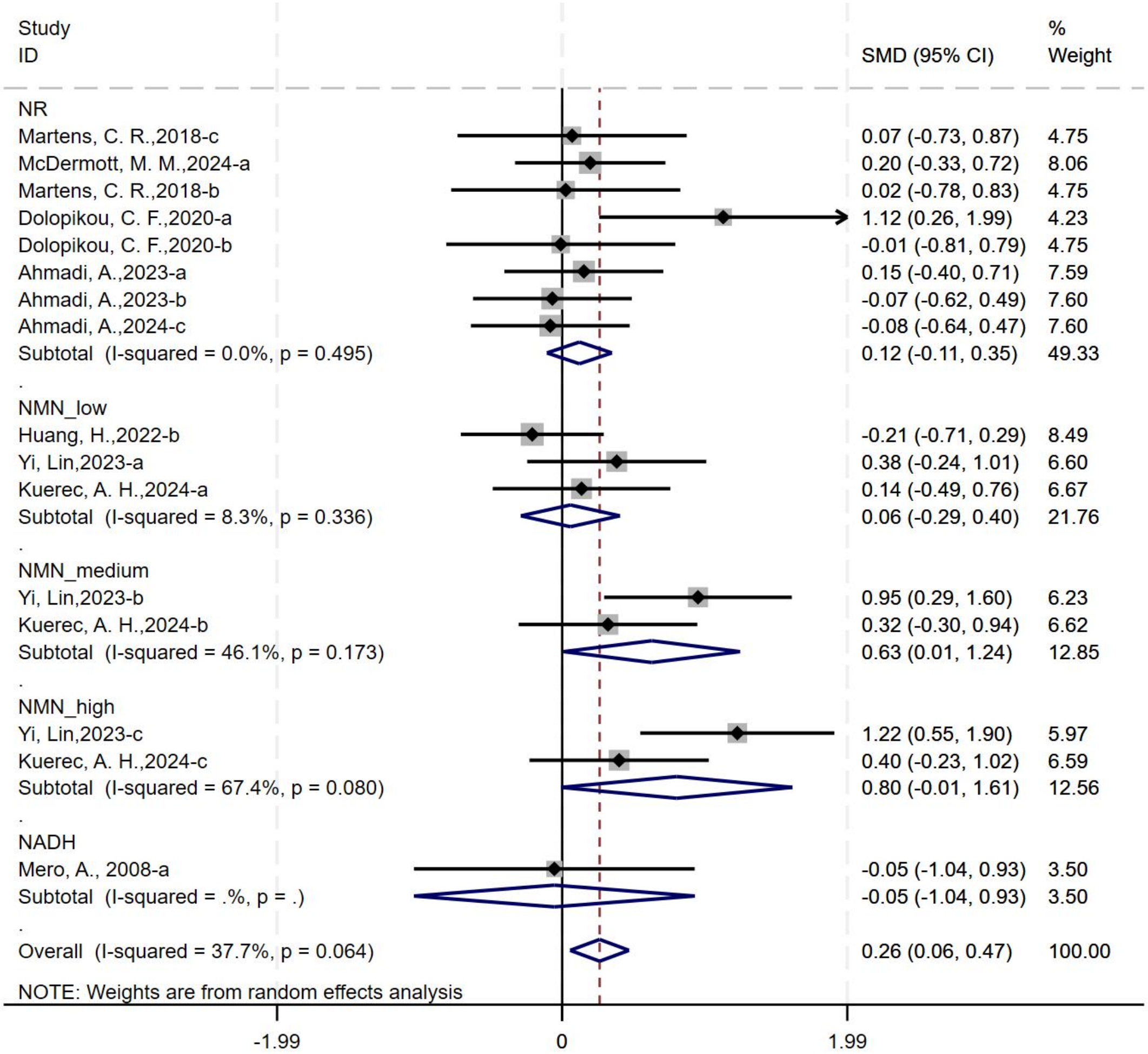

Figure S14 Forest plot of pairwise meta-analysis for aerobic capacity and endurance

4.2.2 Network analysis for Aerobic Capacity and Endurance

Global inconsistency test

$$\chi^2 = 2.45$$

$$p = 0.1457$$

No evidence for the existence of significant global inconsistency

Local inconsistency test

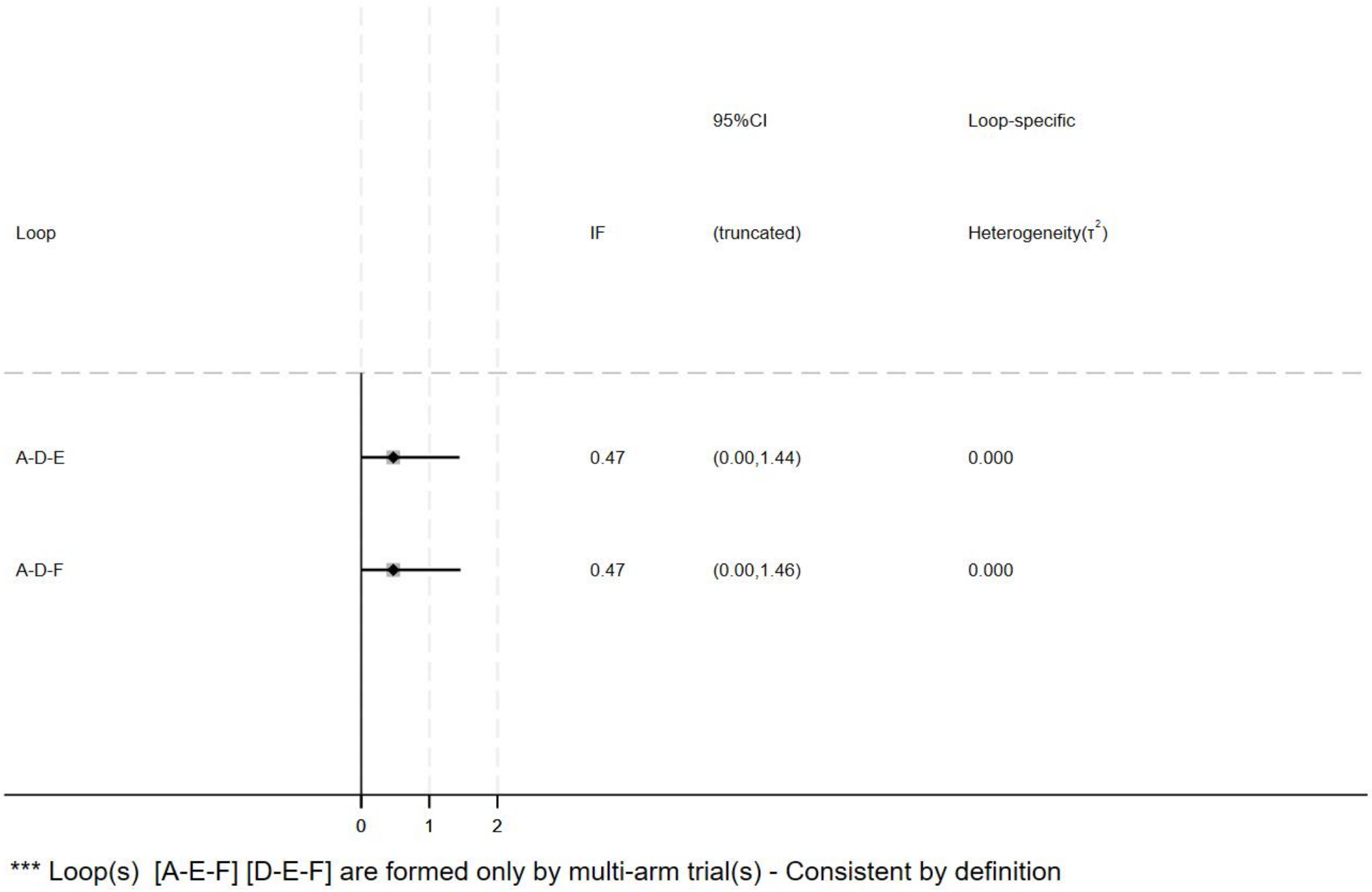

Figure S15 Loop-specific heterogeneity for aerobic capacity and endurance

Note. A means placebo control; B means NADH; C means NR; D means NMN_low; E means NMN_medium; F means NMN_high

The comparisons for different types of NAD^+ precursors and placebo control group

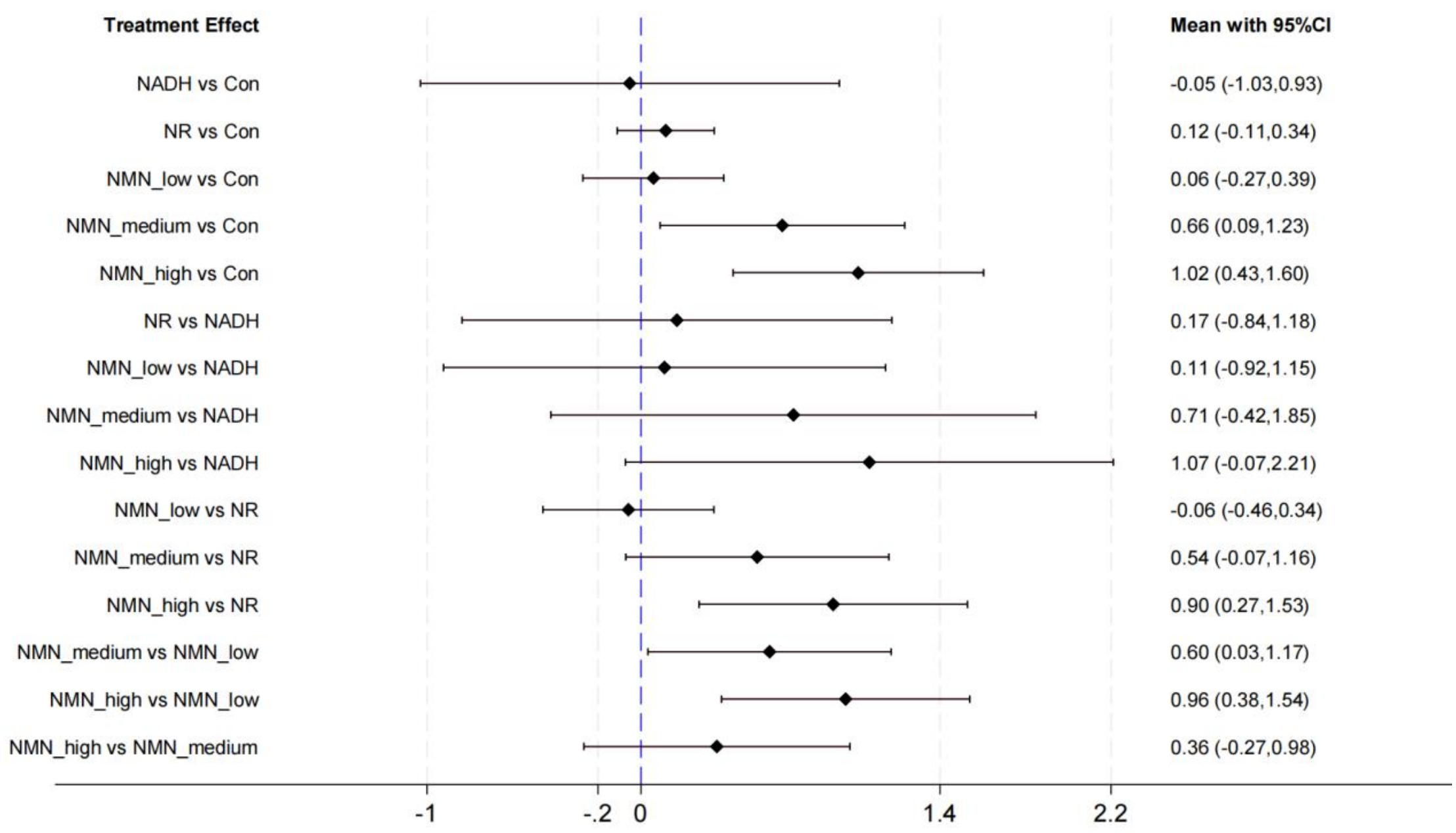

Figure S16 Interval plot of network meta-analysis for aerobic capacity and endurance

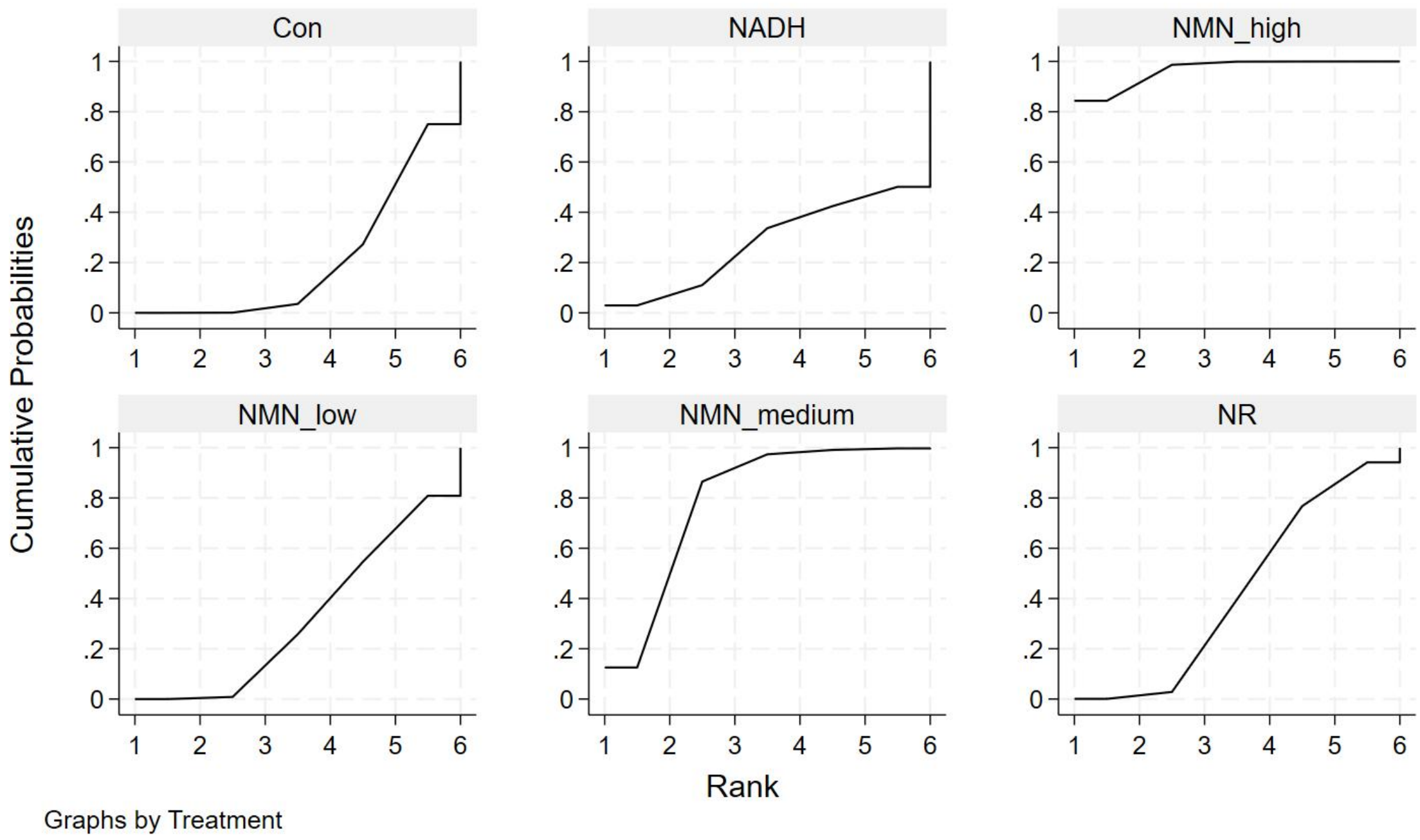

Figure S17 Cumulative ranking plot for aerobic capacity and endurance

Table S7 The ranking of effects of different types of NAD^+ precursors on aerobic capacity and endurance

Treatment	Physical performance<br>Aerobic capacity & endurance		
	**SUCRA**	**Mean Rank**	**Probability of Best (%)**
Control	21.2	0.0	4.9
NADH	28.0	3.0	4.6
NR	42.7	0.1	3.9
NMN_low	32.4	0.0	4.4
NMN_medium	79.1	12.6	2.0
NMN_high	96.6	84.4	1.2

4.2.3 Publication Bias for Aerobic Capacity and Endurance

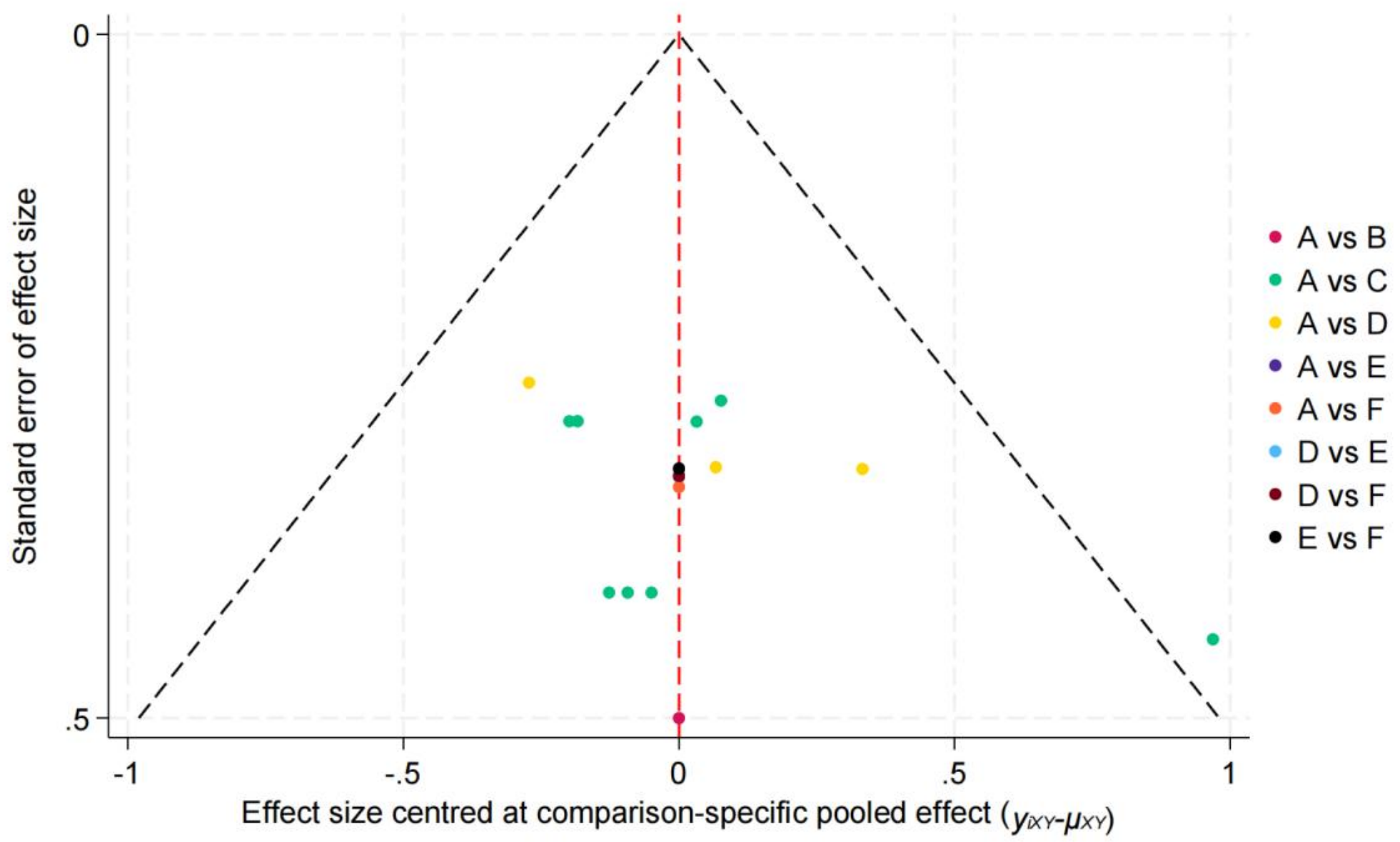

Figure S18 Funnel Plot for aerobic capacity and endurance

Note. A means placebo control; B means NADH; C means NR; D means NMN_low; E means NMN_medium; F means NMN_high

4.3 Effects on Motor performance fatigue--Neuromuscular strength

4.3.1 Pairwise analysis for Neuromuscular strength

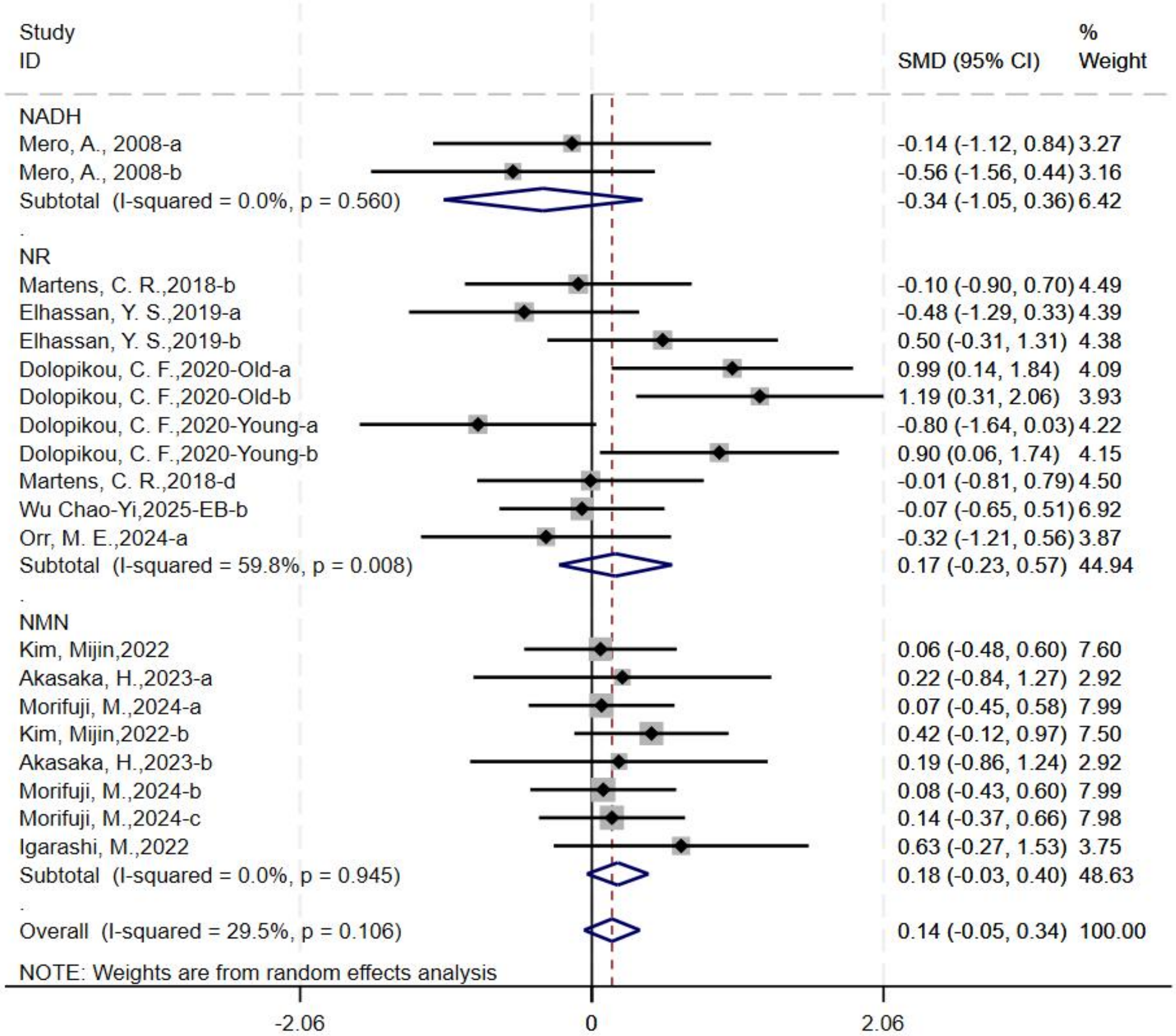

Figure S19 Forest plot of pairwise meta-analysis for neuromuscular strength

4.3.2 Network analysis for Neuromuscular strength

Global inconsistency test

$$\chi^2 = 1.79$$

$$p = 0.1985$$

No evidence for the existence of significant global inconsistency

The comparisons for different types of NAD^+ precursors and placebo control group

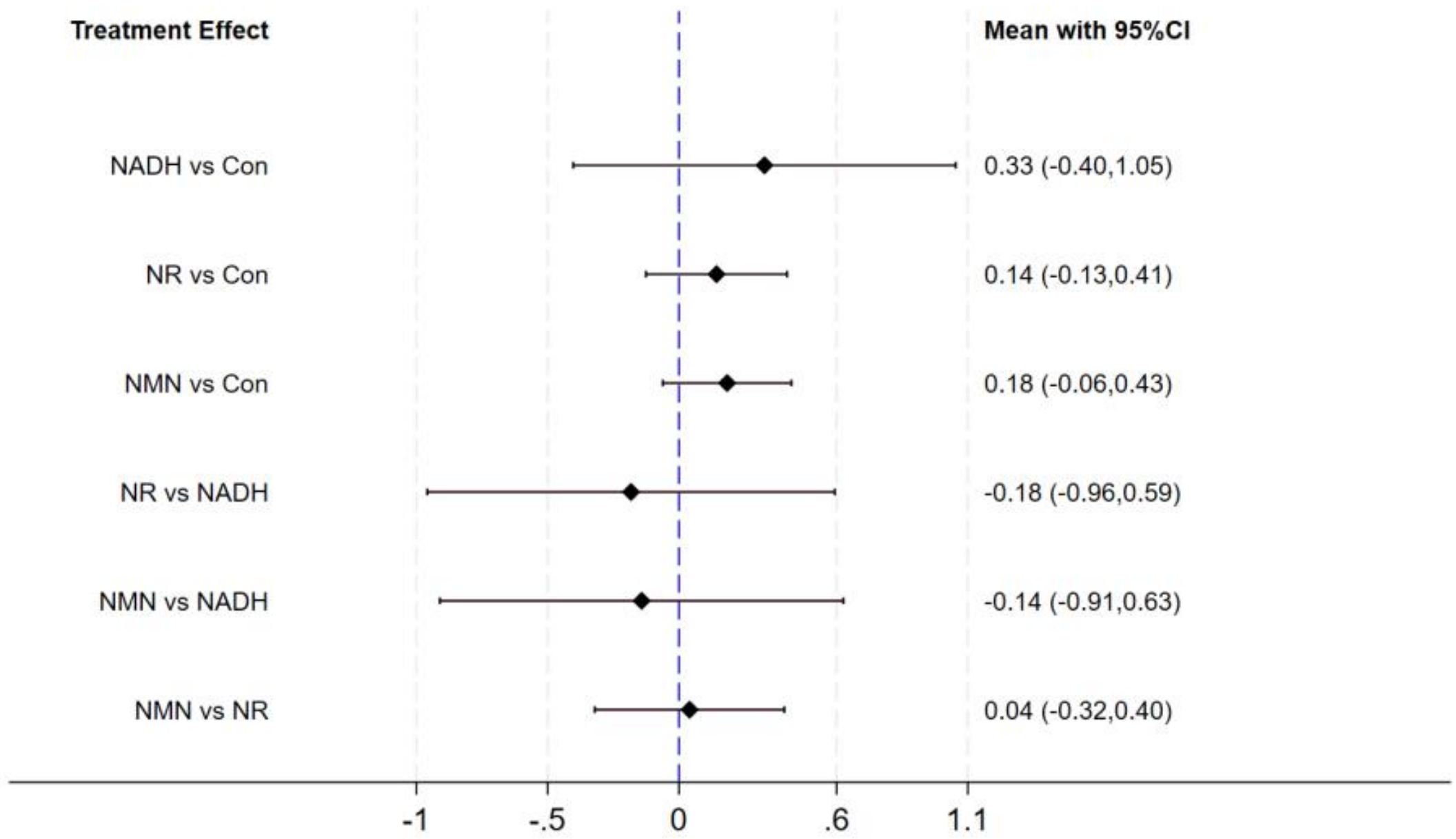

Figure S20 Interval plot of network meta-analysis for neuromuscular strength

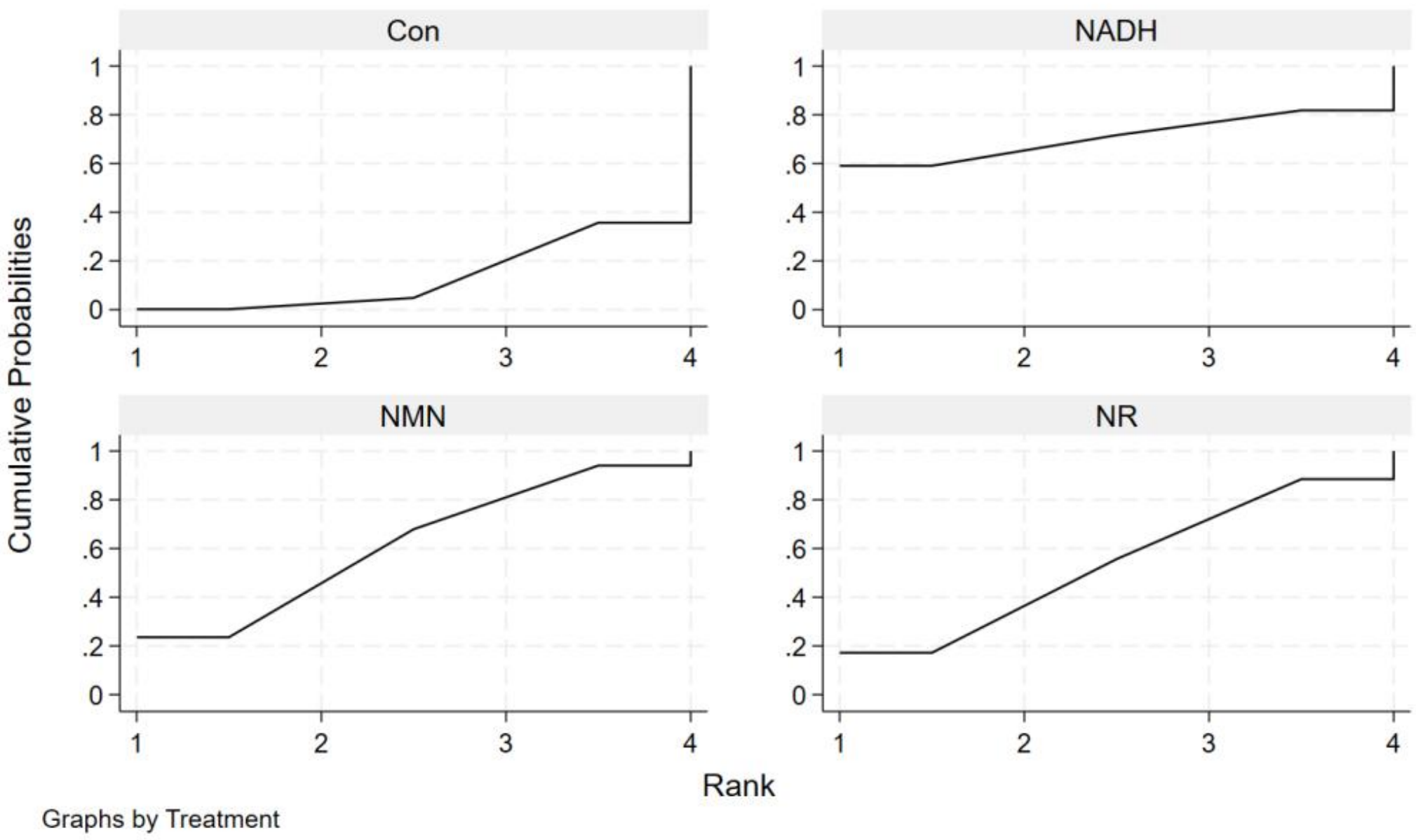

Figure S21 Cumulative ranking plot for neuromuscular strength

Table S8 The ranking of effects of different types of NAD+ precursors on neuromuscular strength

**Treatment**	**Physical performance**
	**Neuromuscular Strength**

	SUCRA	Mean Rank	Probability of Best (%)
Control	13.5	3.6	0.2
NADH	70.8	1.9	59.0
NR	53.8	2.4	17.2
NMN	61.9	2.1	23.6

4.3.3 Publication Bias for Neuromuscular strength

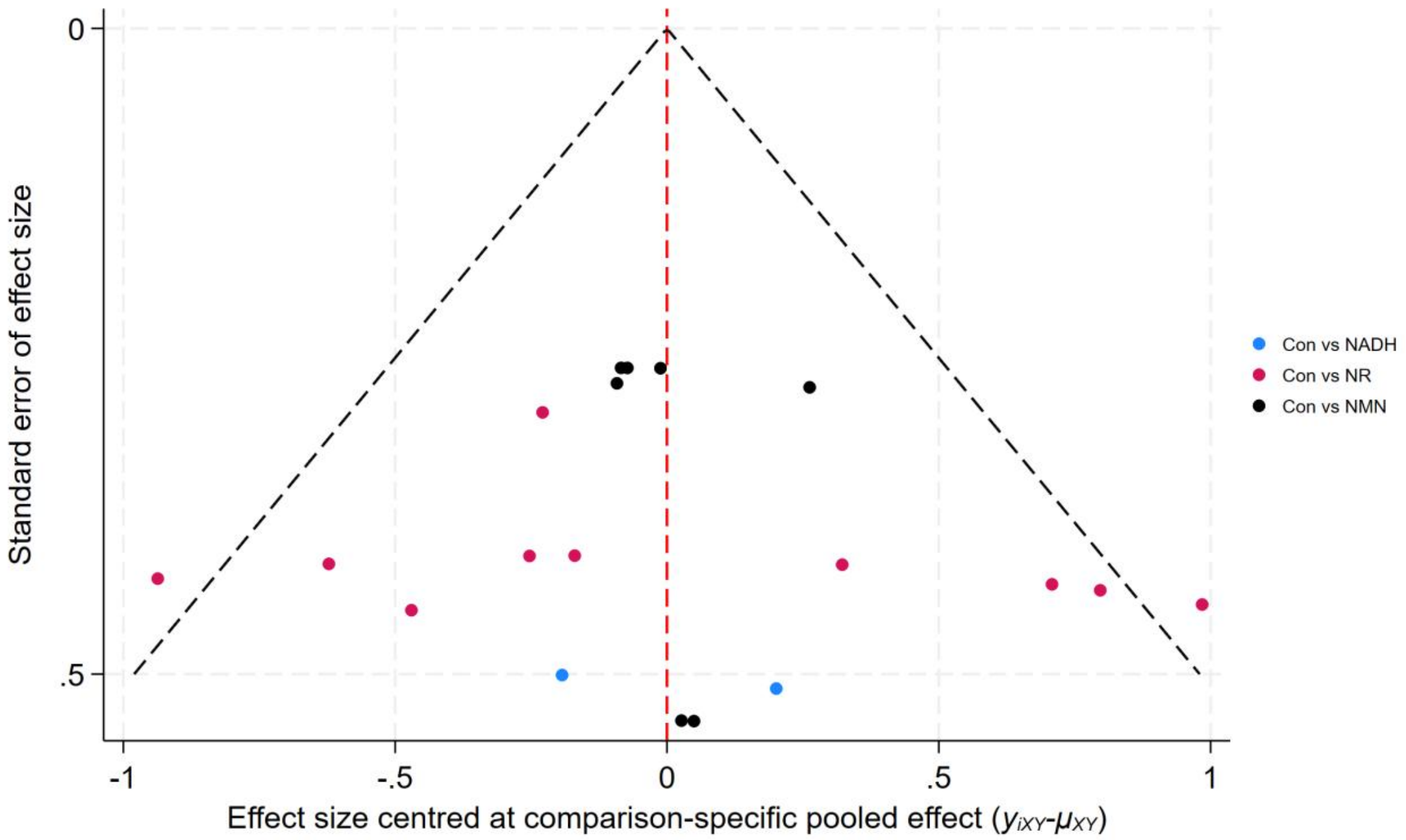

Figure S22 Funnel Plot for neuromuscular strength

4.4 Effects on Cognitive performance fatigue

4.4.1 Pairwise analysis for cognitive performance fatigue

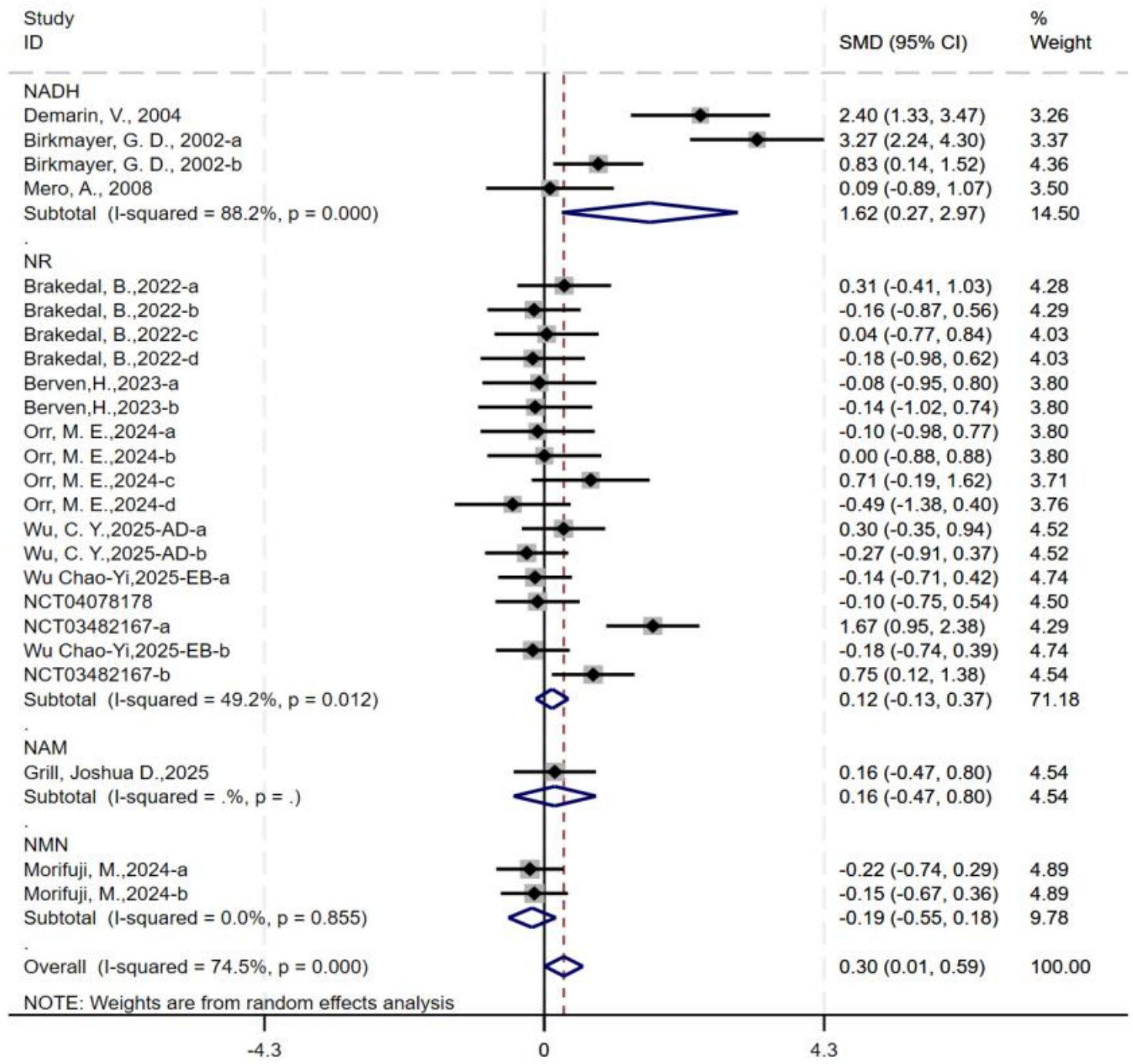

Figure S23 Forest plot of pairwise meta-analysis for cognitive performance fatigue

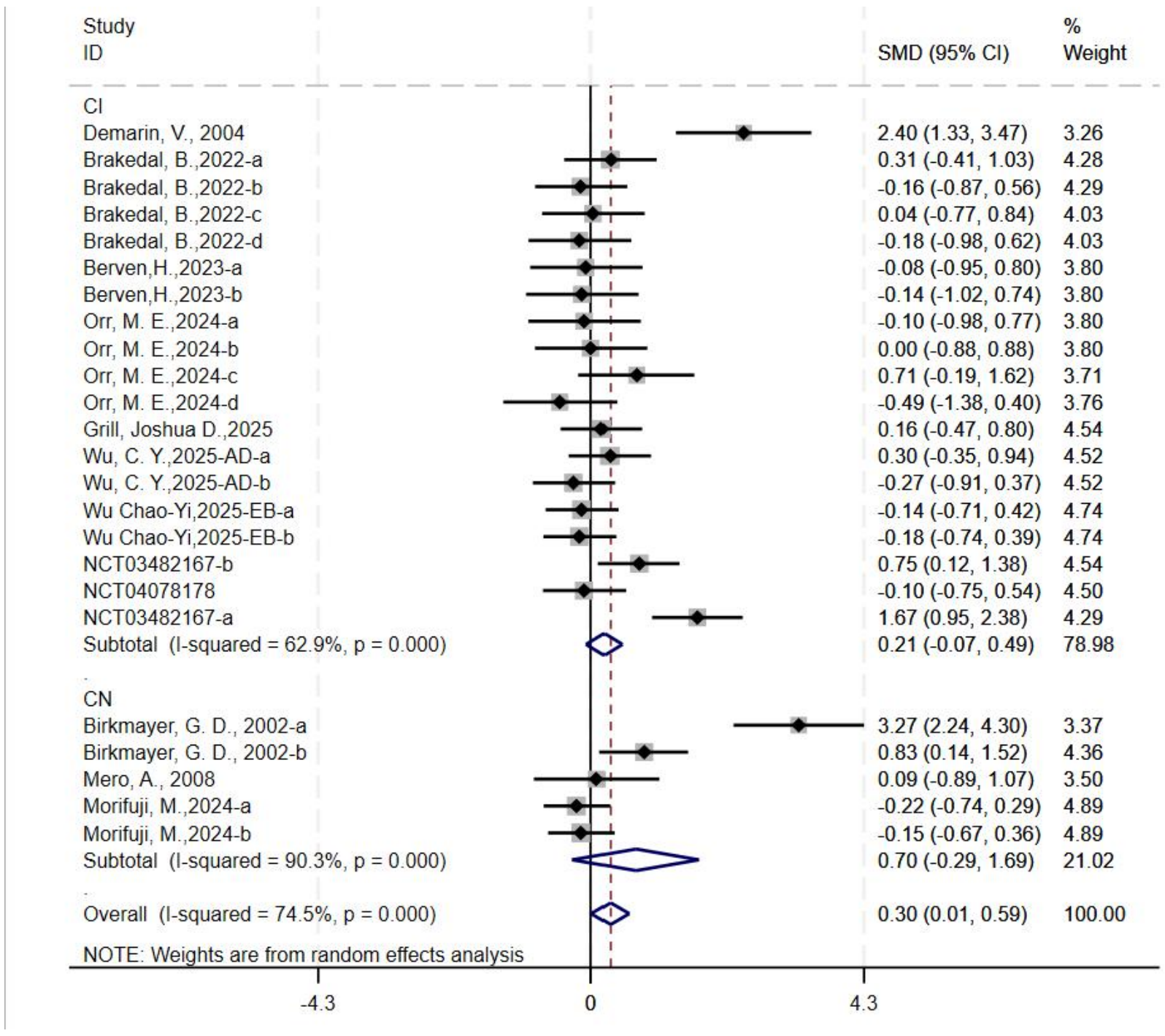

Figure S24 Forest plot of pairwise meta-analysis for cognitive performance fatigue in cognitively impaired & cognitively normal group.

4.4.2 Network analysis for cognitive performance fatigue

Global inconsistency test

χ^2 = 0.06

***p* = 0.8053**

No evidence for the existence of significant global inconsistency

The comparisons for different types of NAD^+ precursors and placebo control group

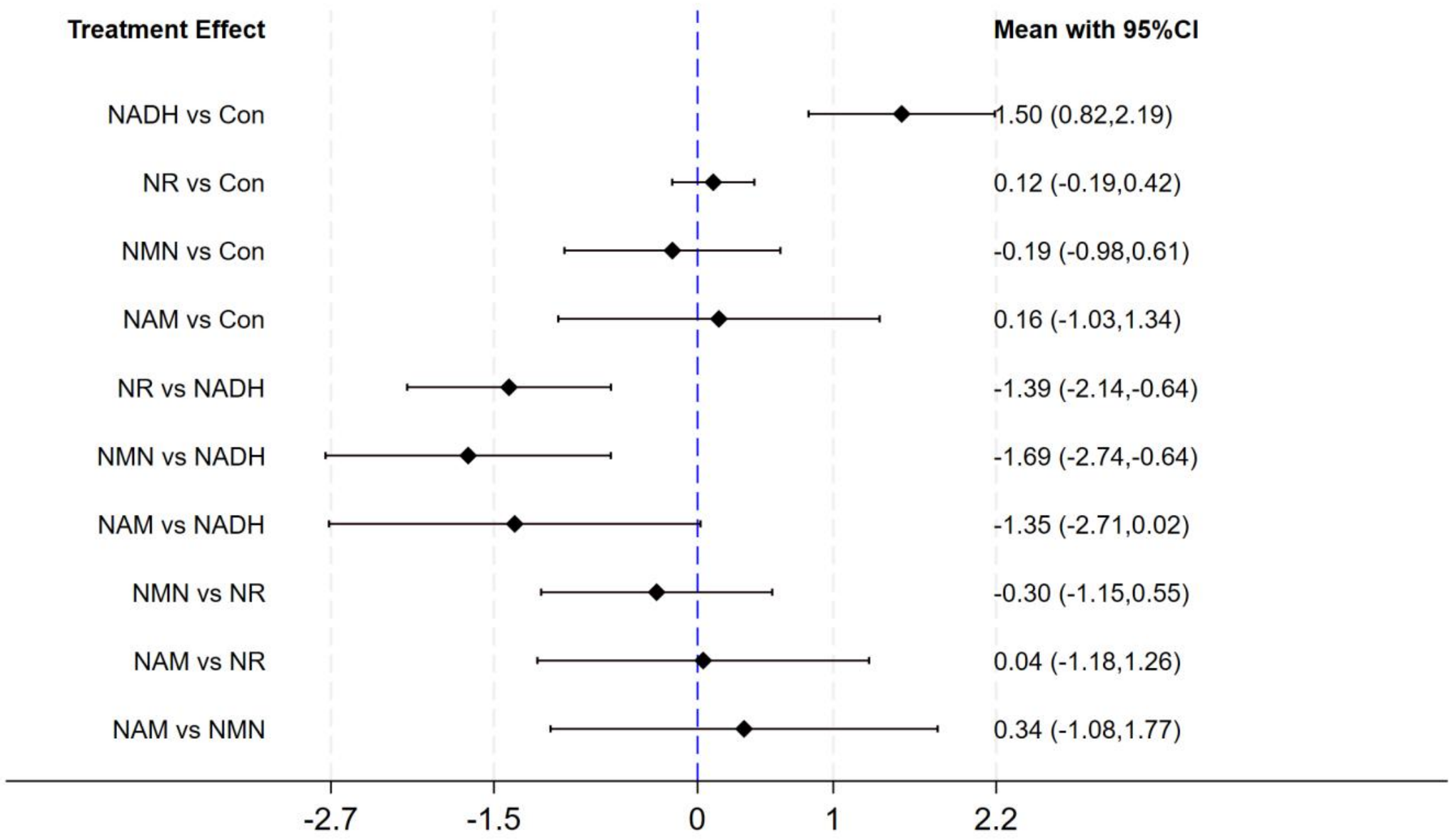

Figure S25 Interval plot of network meta-analysis for cognitive performance fatigue

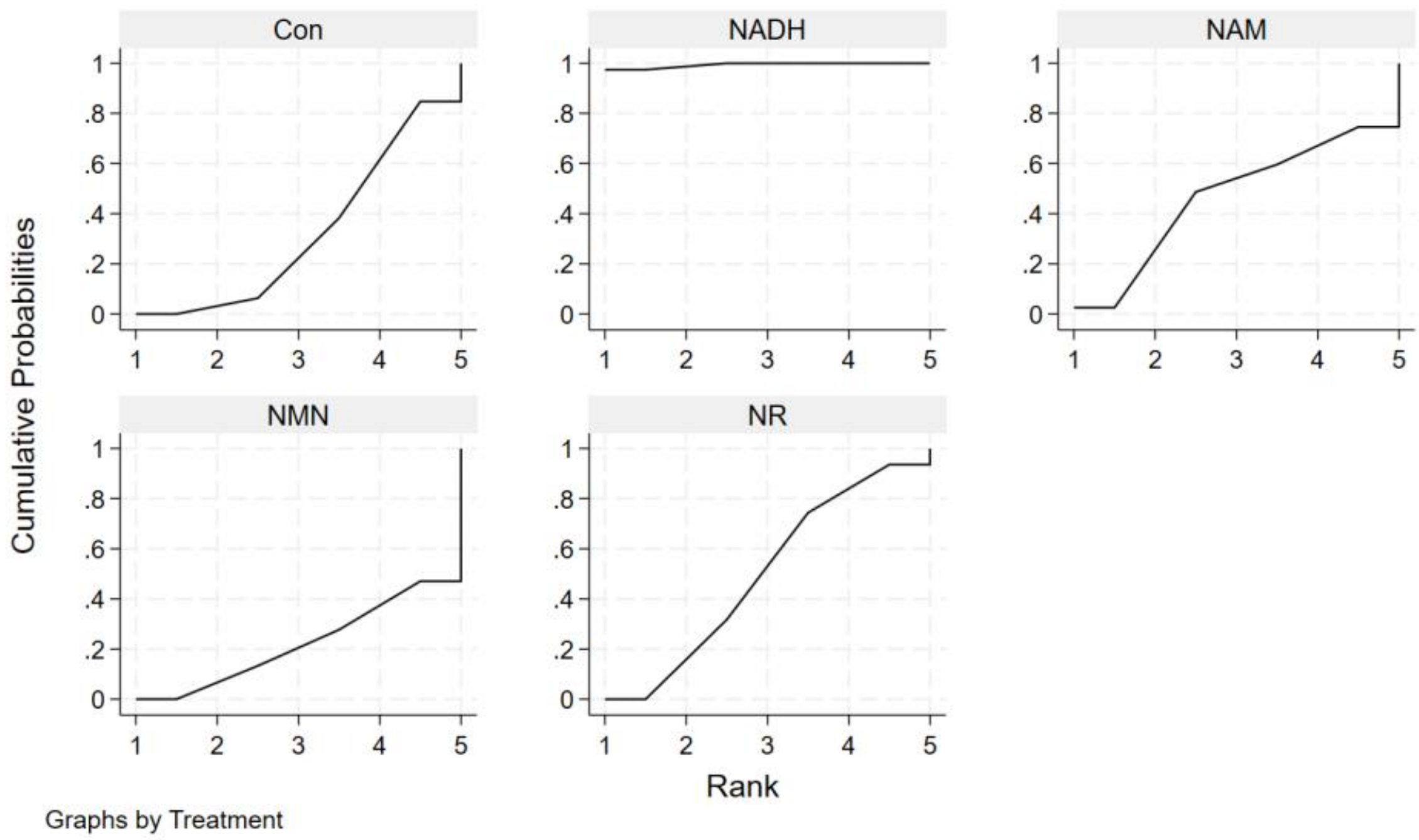

Figure S26 Cumulative ranking plot for cognitive performance fatigue

Table S9 The ranking of effects of different types of NAD+ precursors on cognitive performance fatigue

Treatment	Cognitive performance Fatigue		
	**SUCRA**	**Mean Rank**	**Probability of Best (%)**
Control	32.3	3.7	0.0
NADH	99.4	1.0	97.4
NR	49.9	3.0	0.0
NMN	22.0	4.1	0.0
NAM	46.3	3.1	2.5

4.4.3 Subgroup analysis for Cognitive Performance Fatigue in Cognitive impaired group

Global inconsistency test

$$\chi^2 = 0.12$$

$$p = 0.7364$$

No evidence for the existence of significant global inconsistency

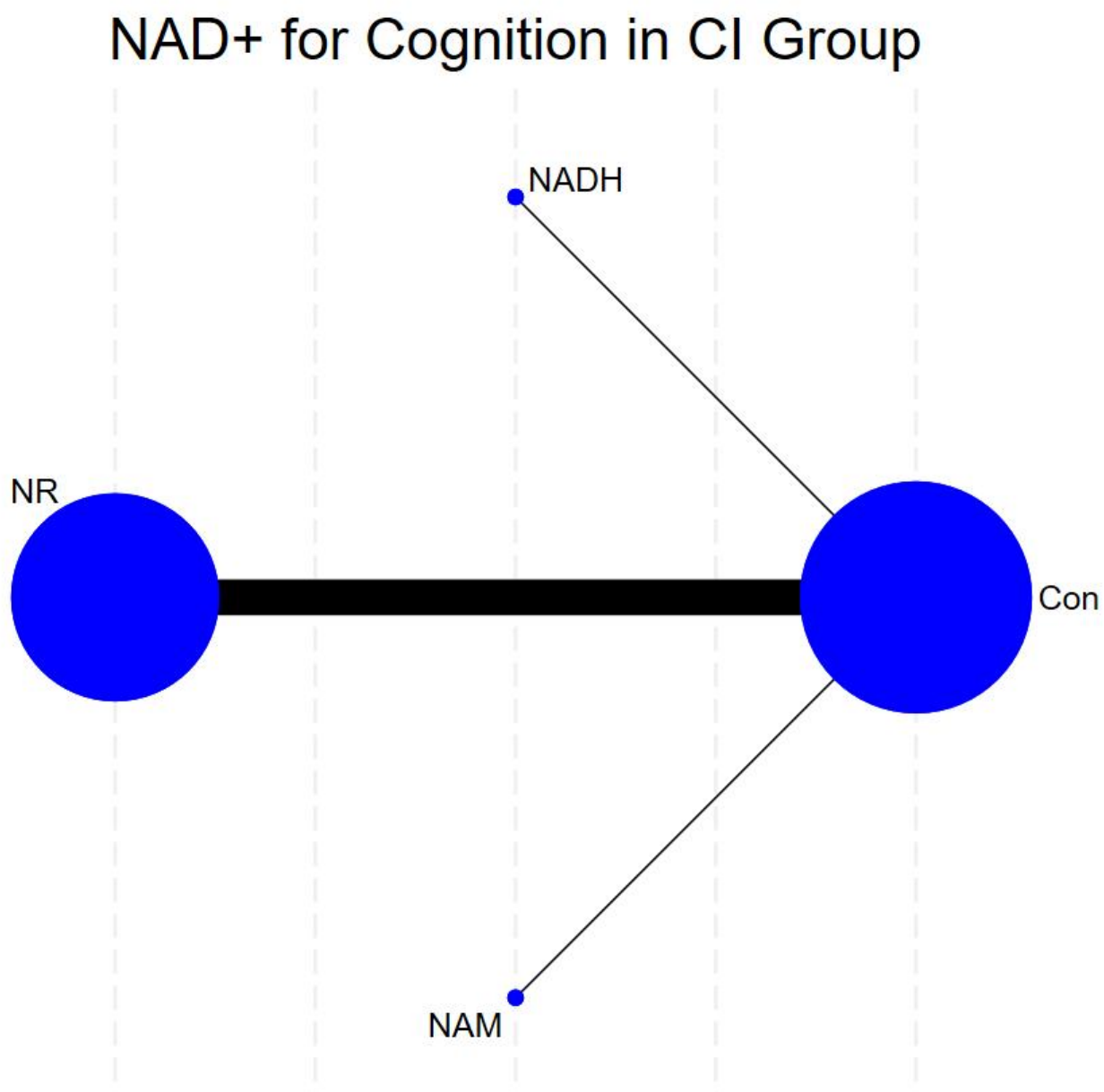

Figure S27 Network of eligible comparisons for cognitive performance fatigue in cognitively impaired group

The comparisons for different types of NAD^+ precursors and placebo control

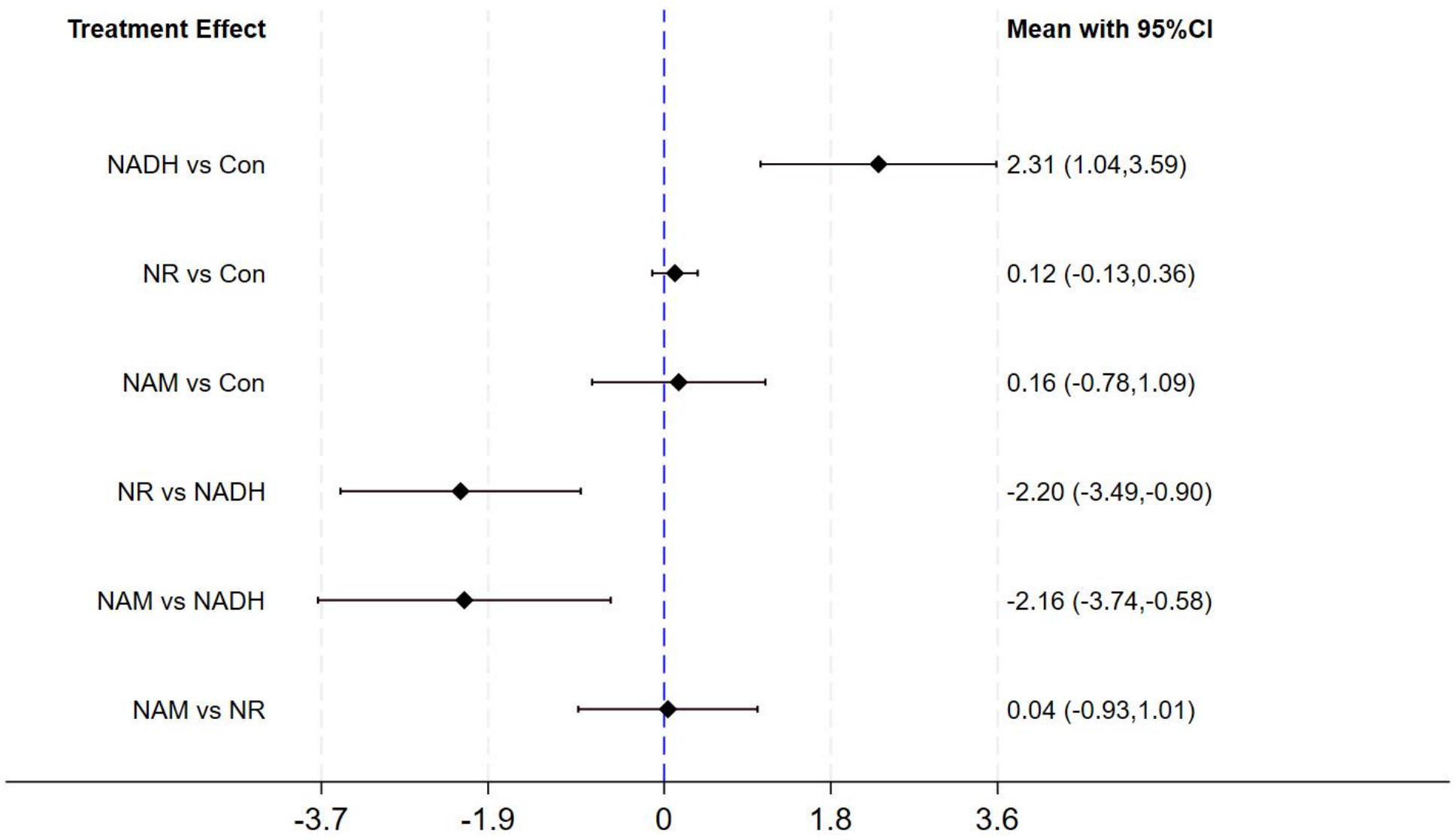

Figure S28 Interval plot of network meta-analysis for cognitive performance fatigue in cognitively impaired group

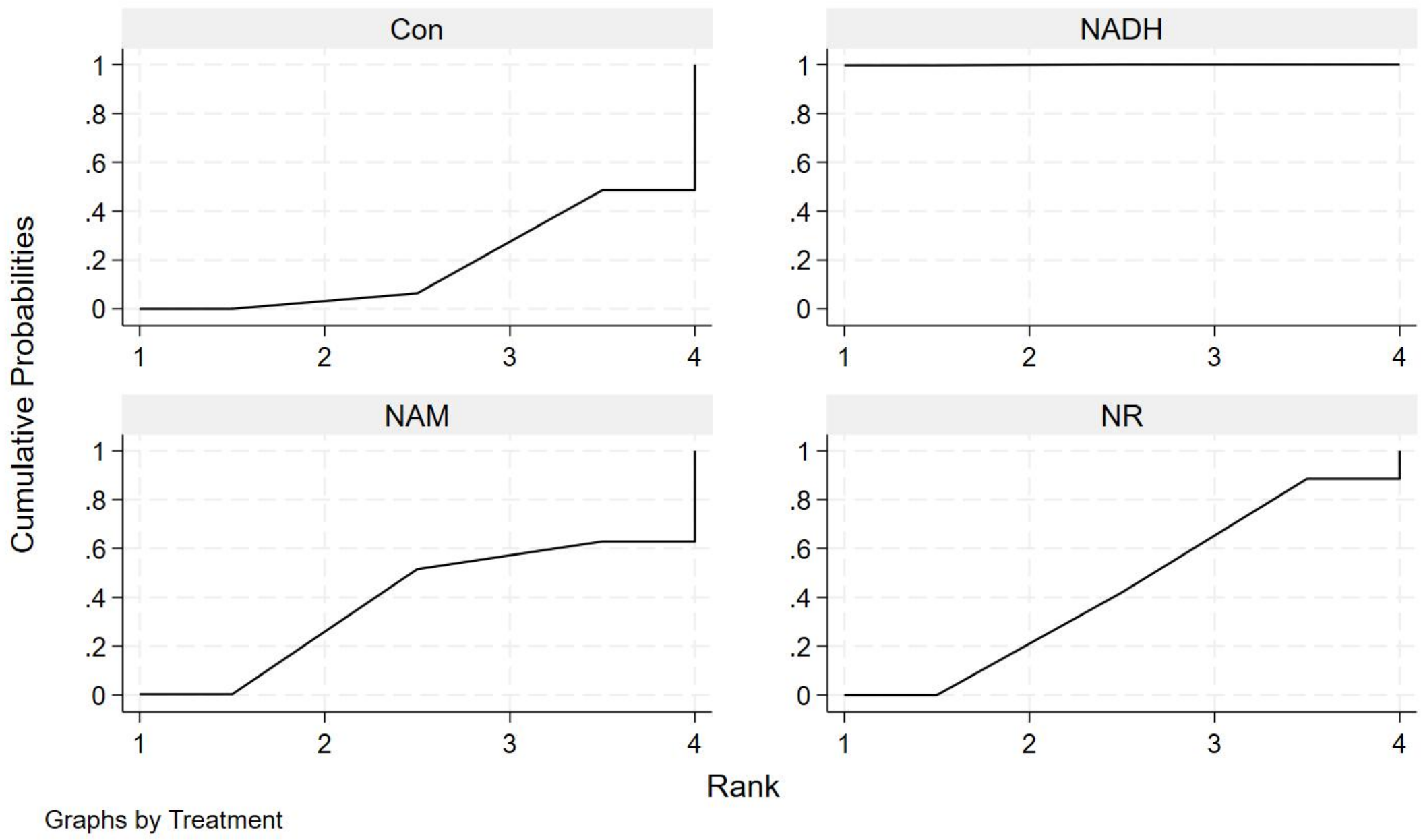

Figure S29 Cumulative ranking plot for cognitive performance fatigue in cognitively impaired group

Table S10 The ranking of effects of different types of NAD^+ precursors on cognitive performance fatigue in cognitively impaired group

Treatment	Perceived Fatigue		
	**SUCRA**	**Mean Rank**	**Probability of Best (%)**
Control	18.3	3.5	0.0
NADH	99.9	1.0	99.7
NR	43.5	2.7	0.0
NAM	38.2	2.9	0.3

4.4.4 Subgroup analysis for Cognitive Performance Fatigue in Cognitive Normal (CN) group

Global inconsistency test

$$\chi^2 = 0.05$$

$$p = 0.8292$$

No evidence for the existence of significant global inconsistency

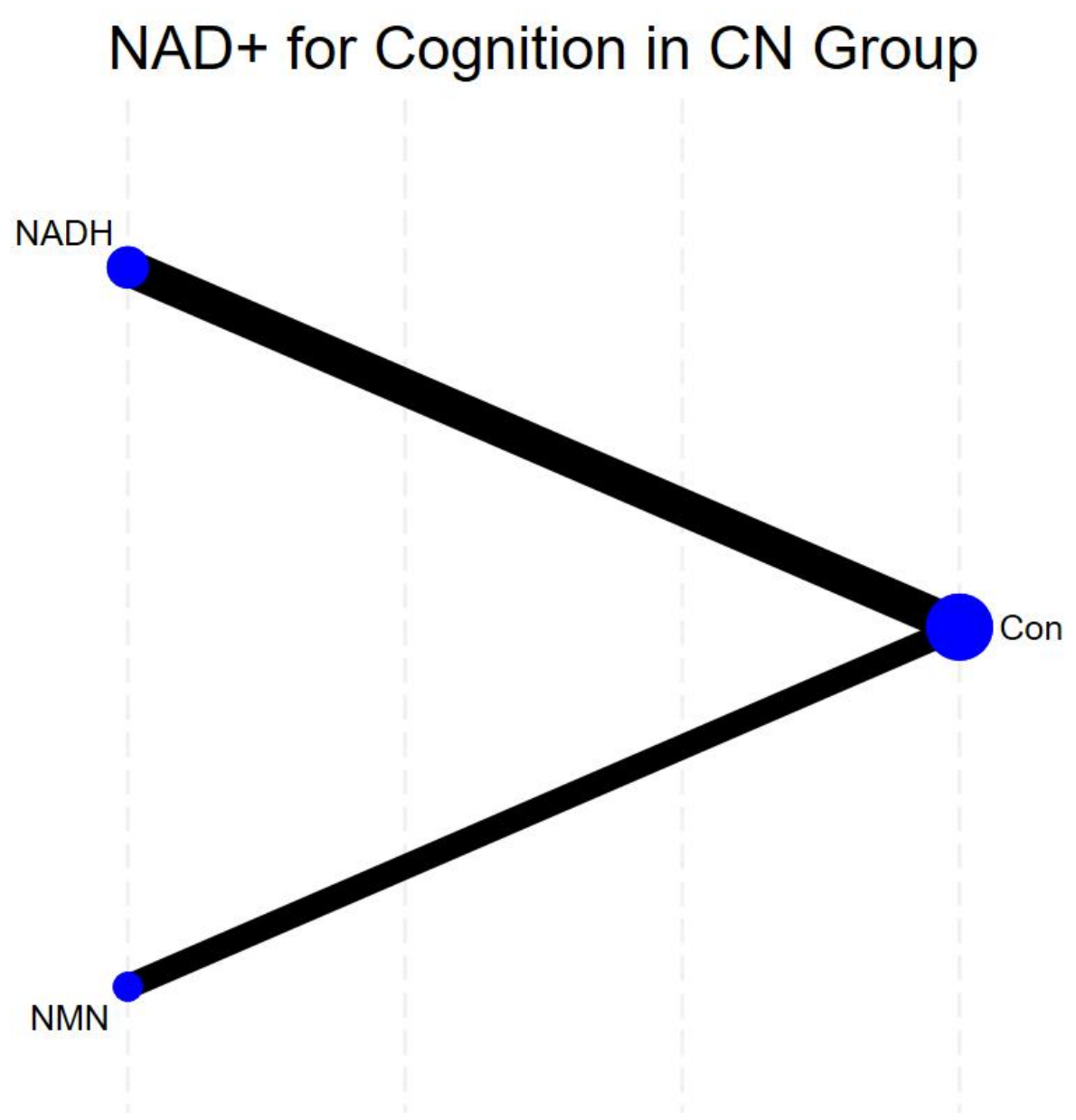

Figure S30 Network of eligible comparisons for cognitive performance fatigue in CN group

The comparisons for different types of NAD^+ precursors and placebo control

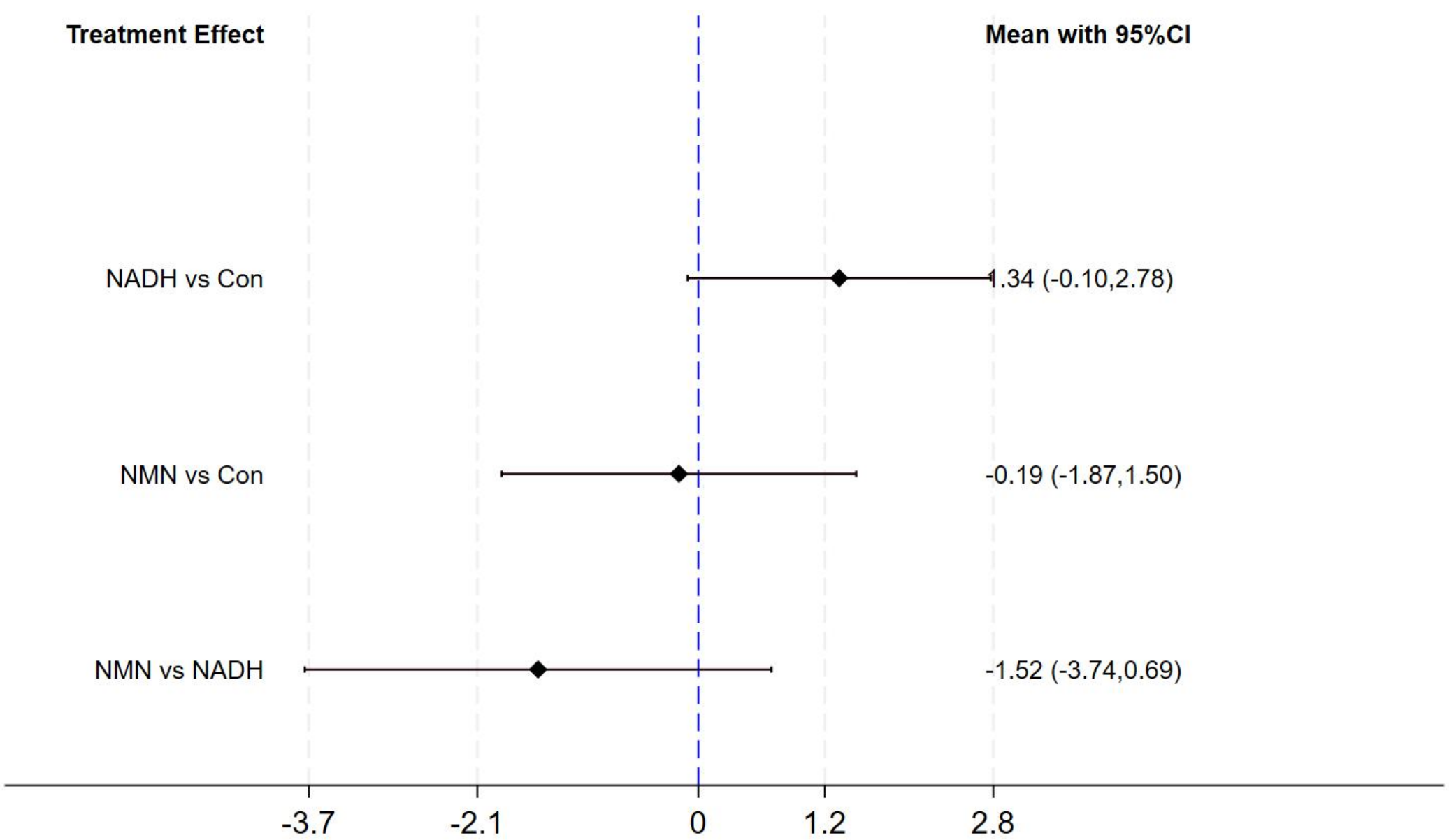

Figure S31 Interval plot of network meta-analysis for cognitive performance fatigue in CN group

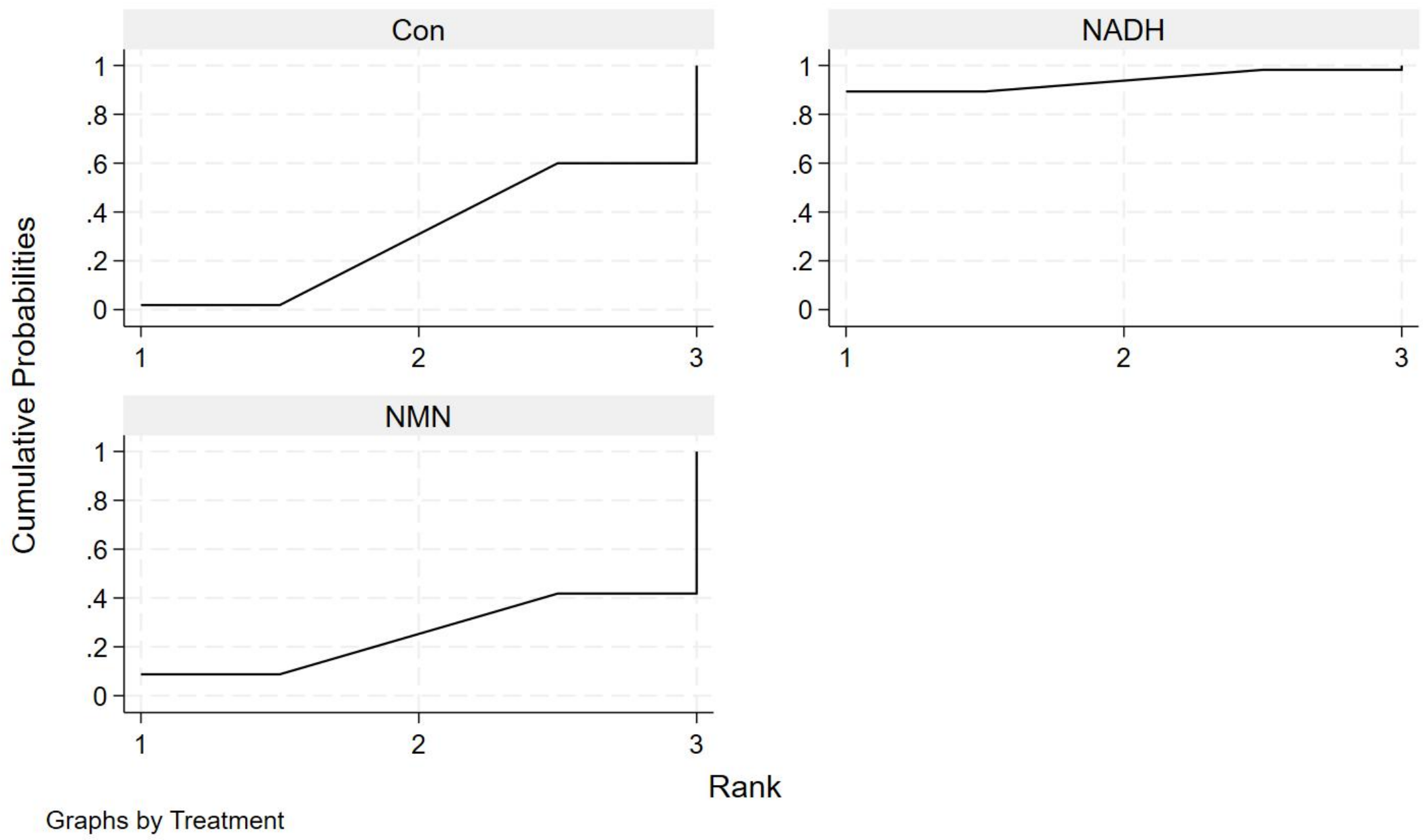

Figure S32 Cumulative ranking plot for cognitive performance fatigue in CN group

Table S11 The ranking of effects of different types of NAD^+ precursors on cognitive performance fatigue in CN group

**Treatment**	**Perceived Fatigue**		
	**SUCRA**	**Mean Rank**	**Probability of Best (%)**
Control	30.9	2.4	1.9
NADH	93.8	1.1	89.3
NMN	25.3	2.5	8.8

4.4.5 Sensitivity analysis for Cognitive Performance Fatigue

(1) Influence analysis

Table S12. Sensitivity Analysis for Cognitive Performance Fatigue in the Network Meta-Analysis

Sensitivity Scenario	NADH vs. Control (SMD [95% CI])	SUCRA Ranking Order (Best to Worst)	Network I^2 (%)	τ^2	Pairwise I^2 (%)
Baseline (Full Network)	1.69 [0.64, 2.74]	NADH (99.4%) > NR (49.9%) > NAM (46.3%) > Con > NMN	63.10%	0.260	74.5%
Excluding Birkmayer (2002)	1.48 [0.38,2.57]	NADH (98.9%) > NR (49.8%) > NAM (46.2%) > Con > NMN	64.76%	0.284	60.4%
Excluding Demarin (2004)	1.28 [0.51,2.04]	NADH (99.7%) > NR (50.5%) > NAM (46.4%) > Con > NMN	62.45%	0.245	70.2%
Excluding both studies above	2.01 [0.89, 3.13]	NADH (99.8%) > NR (49.9%) > NAM (46.1%) > Con > NMN	69.80%	0.385	45.1%

(2) Sensitivity Analysis after excluding the two quasi-experimental studies

To assess whether inclusion of the two quasi-experimental studies (NCT03482167 and NCT04078178) influenced the robustness of the network estimates, an additional sensitivity analysis was conducted after restricting the evidence to RCTs. Compared with the primary analysis, the treatment hierarchy remained largely unchanged despite minor variations in the ranking of lower-ranked interventions. NADH continued to rank first for improving cognitive performance fatigue, with a pooled effect estimate of SMD = 1.44 (95% CI: 0.89 to 1.99) versus control. Although the estimated treatment effect was slightly attenuated after excluding the quasi-experimental studies, the confidence interval became narrower and the treatment effect remained statistically significant. Similarly, the SUCRA rankings showed minimal variation, supporting the robustness of the primary findings after exclusion of the quasi-experimental studies.

Table S1A. Sensitivity Analysis Excluding the Two Quasi-experimental Studies

Sensitivity Scenario	Baseline Full Network	Excluding Two Quasi-experimental Studies	Interpretation
**Pairwise pooled SMD (Full Network)**	0.30[0.01, 0.59]	0.22[-0.07, 0.52]	Slight attenuation with unchanged direction of effect
**NADH vs. Control (SMD [95% CI])**	1.69 [0.64, 2.74]	1.44 [0.89, 1.99]	Effect remained significant; precision improved
**SUCRA Ranking Order (Best to Worst)**	NADH (99.4%) > NR (49.9%) > NAM (46.3%) > Con (32.3%) > NMN (22.0%)	NADH (99.9%) > NAM (51.9%) > Con (42.8%)> NR (35.7%) > NMN(19.8%)	NADH remained the highest-ranked intervention
**Network I^2**	63.10%	44.12%	Network heterogeneity reduced
**τ^2**	0.260	0.080	Between-study variance markedly reduced
**Pairwise I^2**	74.5%	71.2%	Residual heterogeneity remained

To evaluate the influence of study design on the robustness of the network estimates, an additional sensitivity analysis was conducted after excluding the two

quasi-experimental studies. Compared with the primary analysis, the pooled effect estimate from the pairwise meta-analysis was slightly attenuated, whereas the network estimate for NADH versus control remained statistically significant (SMD = 1.44, 95% CI: 0.89 to 1.99). Importantly, NADH remained the highest-ranked intervention (SUCRA: 99.9%), indicating that the overall treatment hierarchy was robust to exclusion of non-randomized evidence. Furthermore, network heterogeneity decreased substantially (I^2: 63.1% to 44.1%; τ^2: 0.260 to 0.080), whereas pairwise heterogeneity showed only a modest reduction (74.5% to 71.2%). Overall, these findings indicate that the primary conclusions were robust to exclusion of the quasi-experimental studies, although the magnitude of the estimated treatment effect was modestly reduced.

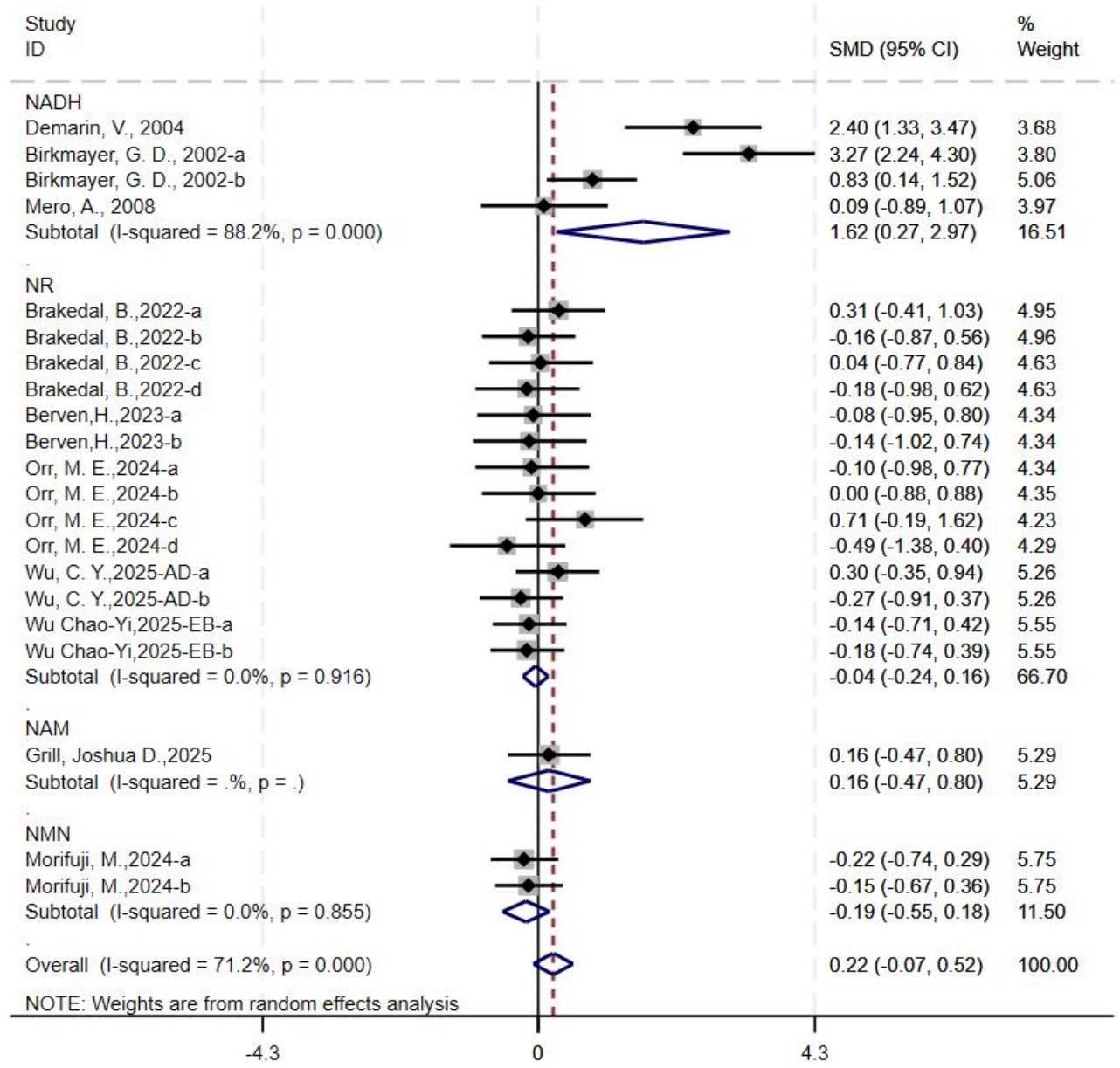

Figure S1A. Forest plot of pairwise meta-analysis for cognitive performance fatigue after excluding the two quasi-experimental studies

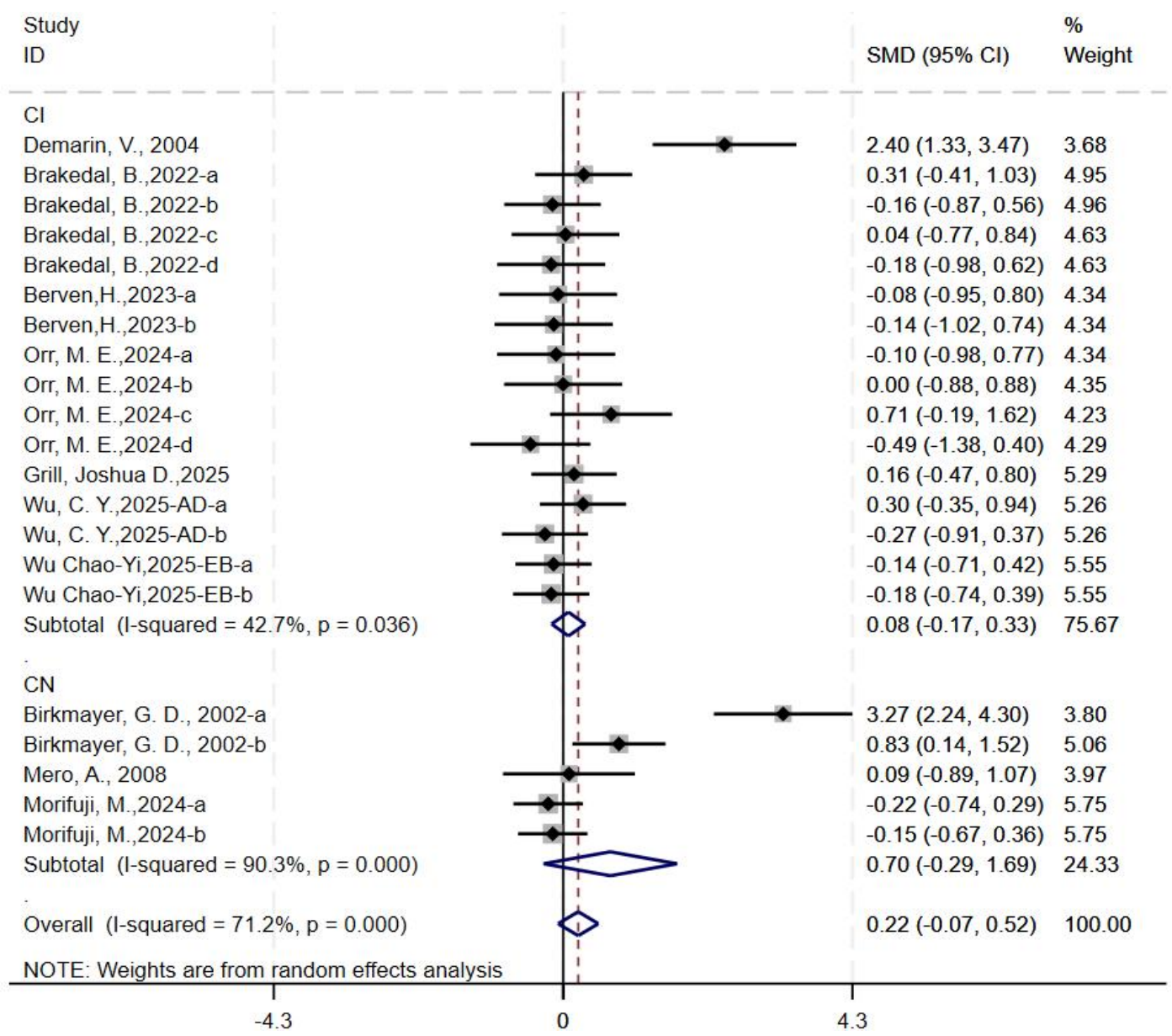

Figure S2A. Forest plot of pairwise meta-analysis for cognitive performance fatigue in cognitively impaired & cognitively normal group after excluding the two quasi-experimental studies.

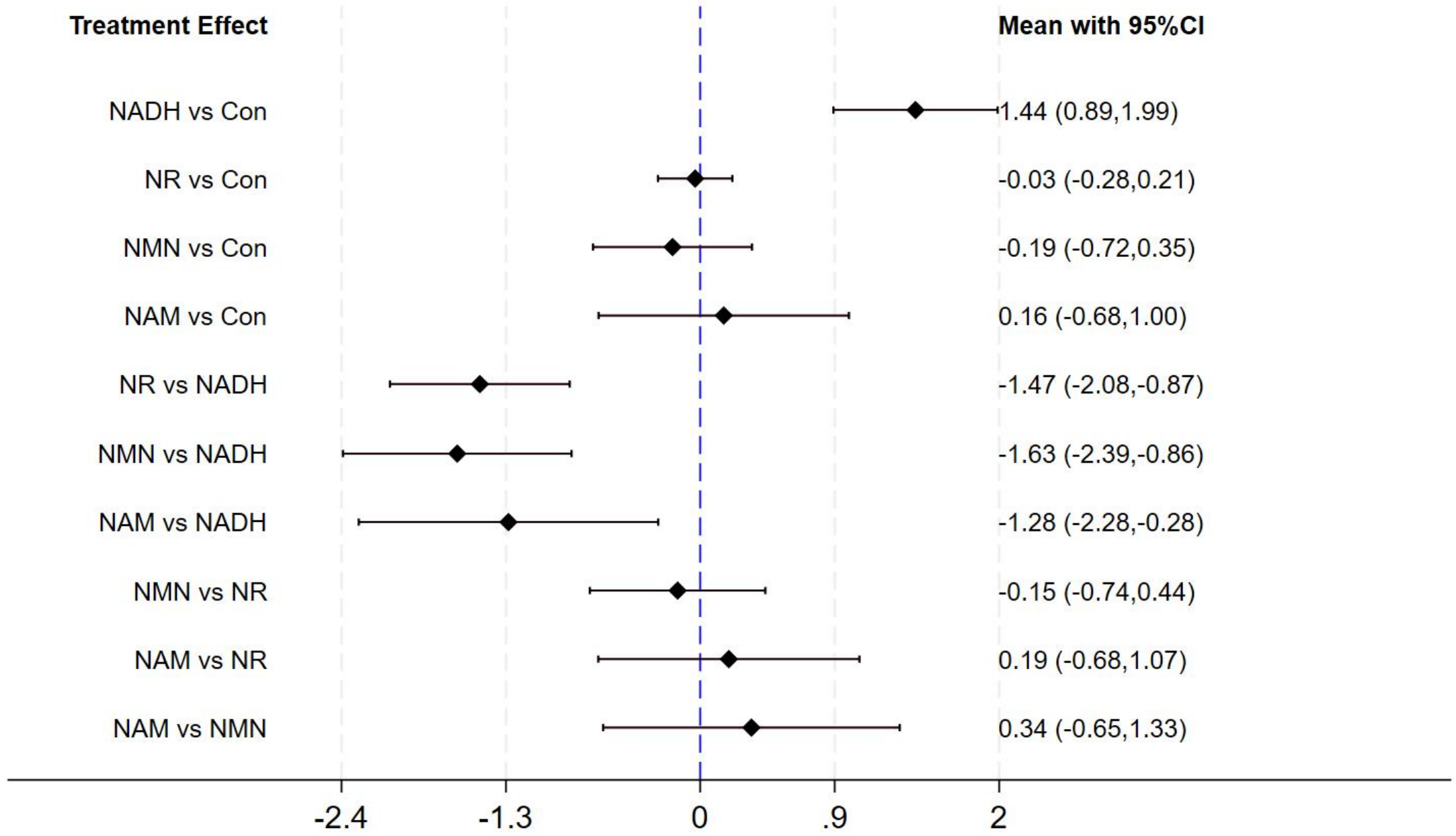

Figure S3A. Interval Plot of the Network Meta-analysis after Excluding the Two Quasi-experimental Studies.

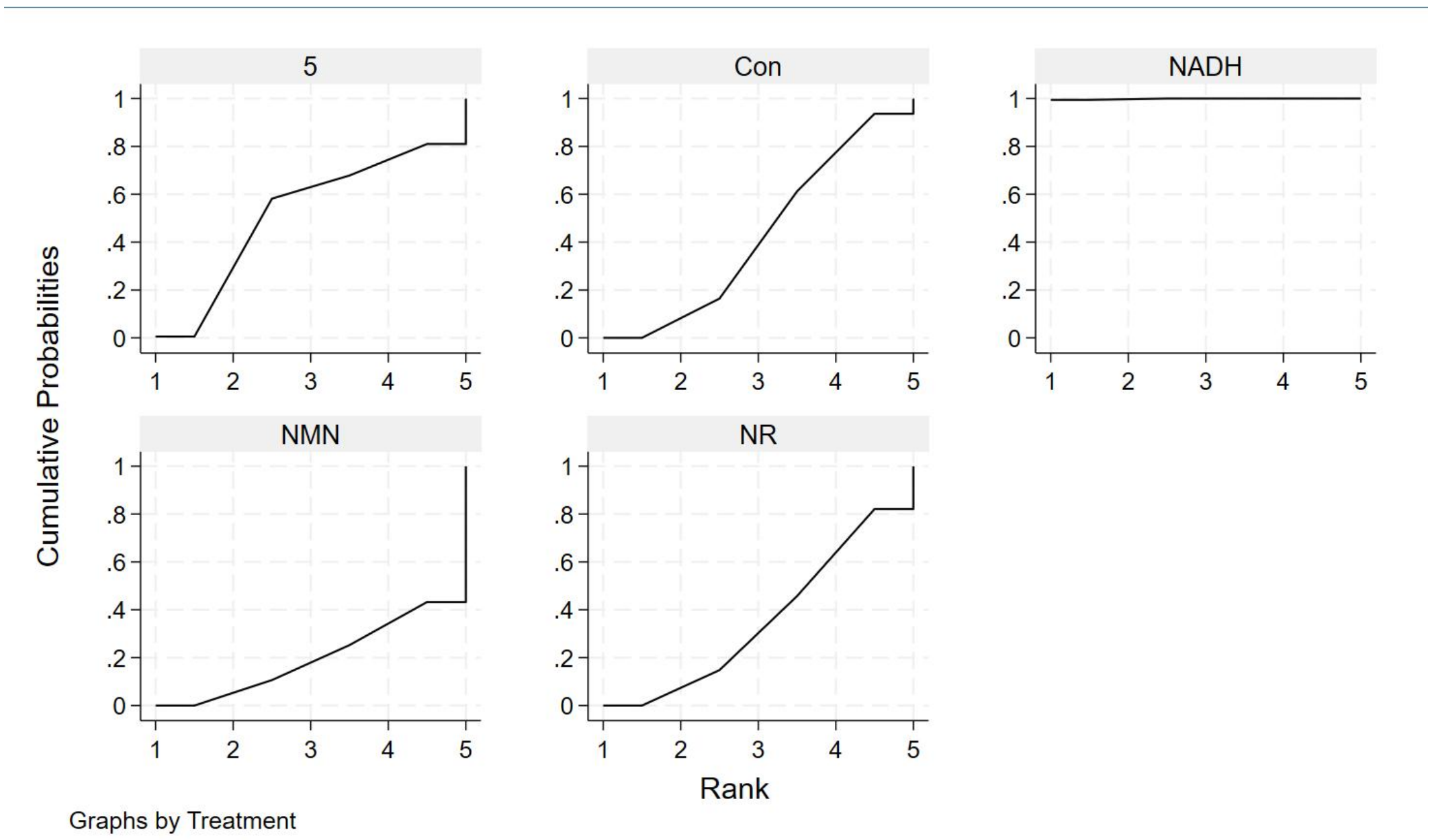

Figure S4A. Cumulative Ranking Plot after Excluding the Two Quasi-experimental Studies

4.4.6 Publication Bias for Cognitive Performance Fatigue

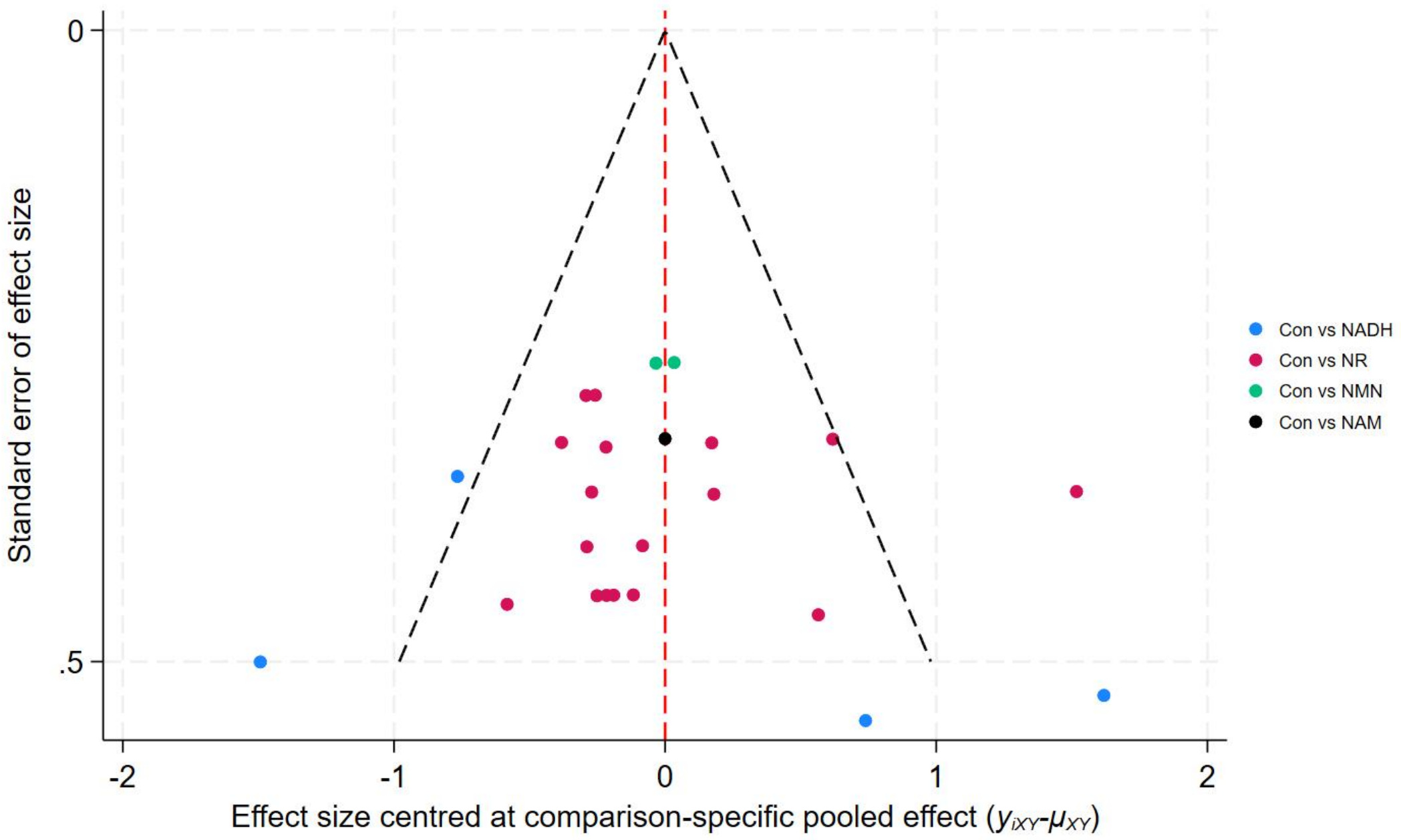

Figure S33 Funnel Plot for cognitive performance fatigue

4.4.7 The results of meta regression analysis

Table S13 Univariate Meta-Regression Analysis for Cognitive Performance

Covariates	Coef.	Std. Error	t	*P*	95% CI
Treatment	-0.8148531	0.2752361	-2.96	0.007	-1.386, -0.244
Dosage (mg/day)	-0.0004	0.00019	-1.86	0.076	-0.000743, 0.00004
Duration (weeks)	-0.0094	0.0238	-0.39	0.697	-0.0583, 0.0396
Participant(CI/CN)	0.4788	0.3962946	1.21	0.887	-0.3431, 1.300617
Disease Type	-0.003882	0.137215	-0.03	0.978	-0.2884486, 0.2806845

4.5 Effects on Quality of life

4.5.1 Pairwise analysis for Quality of life

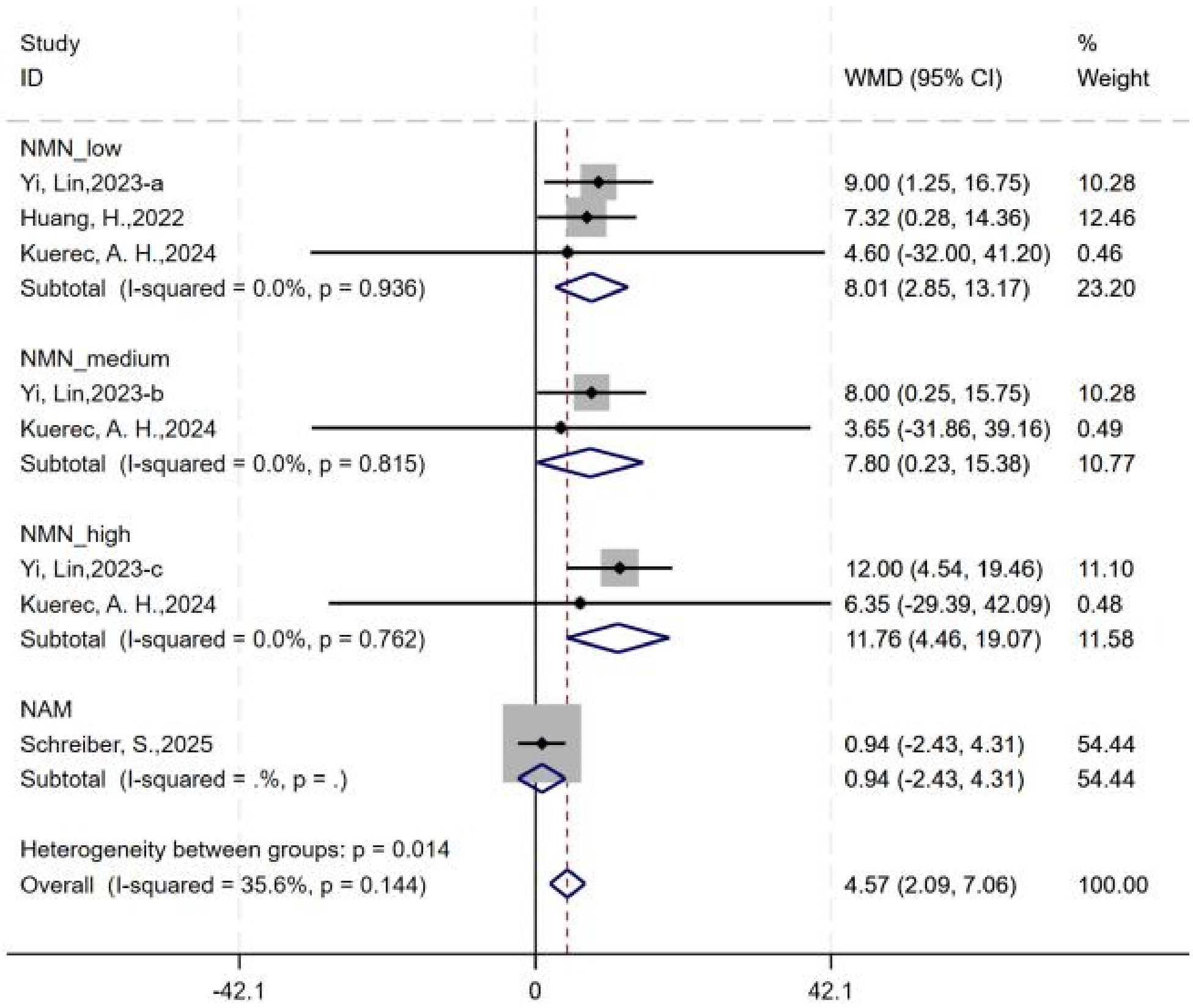

Figure S34 Forest plot of pairwise meta-analysis for quality of life

4.5.2 Network analysis for Quality of life

Global inconsistency test

$$\chi^2 = 0.13$$

$$p = 0.7313$$

No evidence for the existence of significant global inconsistency

Local inconsistency test

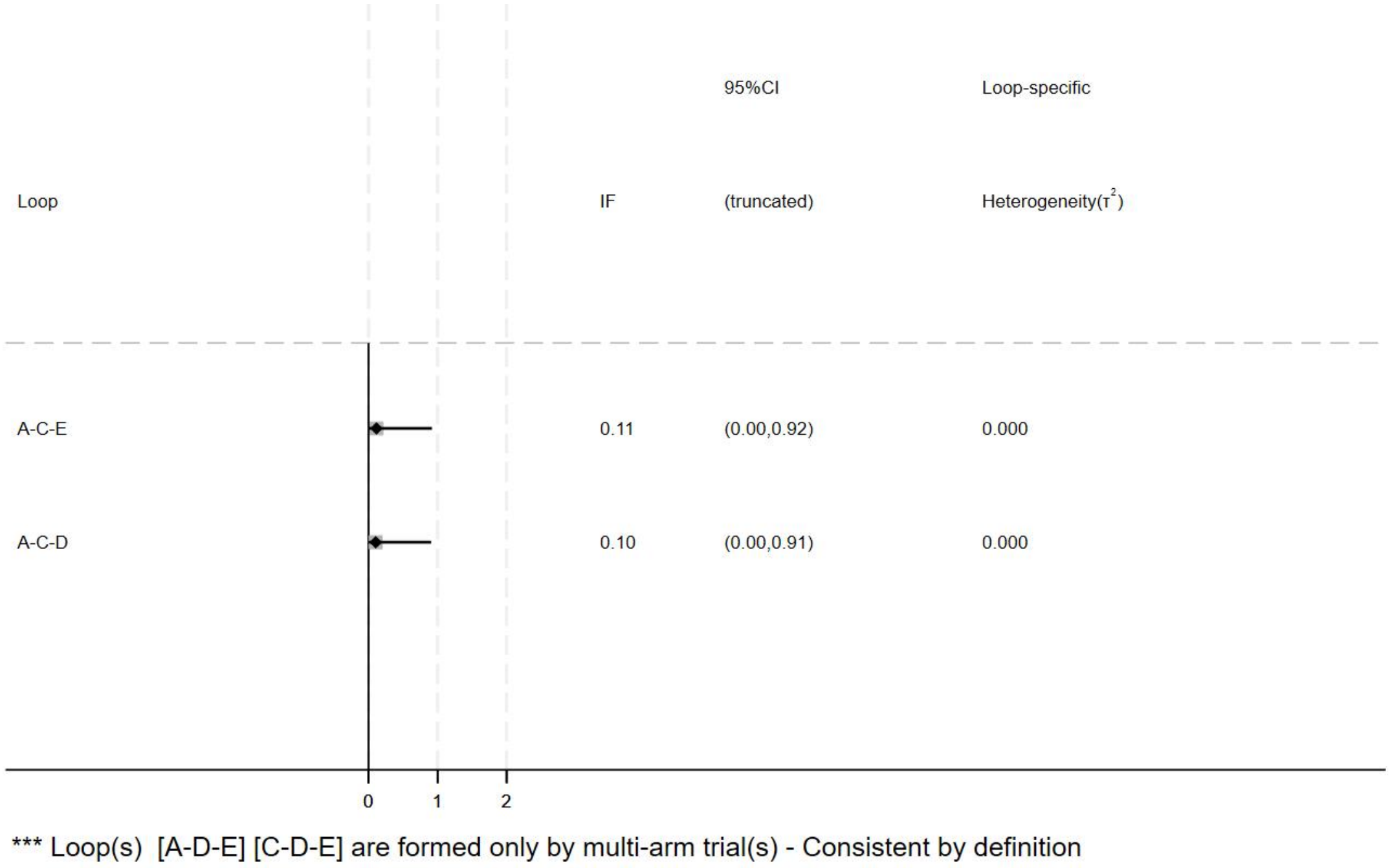

Figure S35 Loop-specific heterogeneity for quality of life

Note. A means placebo control; B means NAM; C means NMN_low; D means NMN_medium; E means NMN_high

The comparisons for different types of NAD^+ precursors and placebo control group

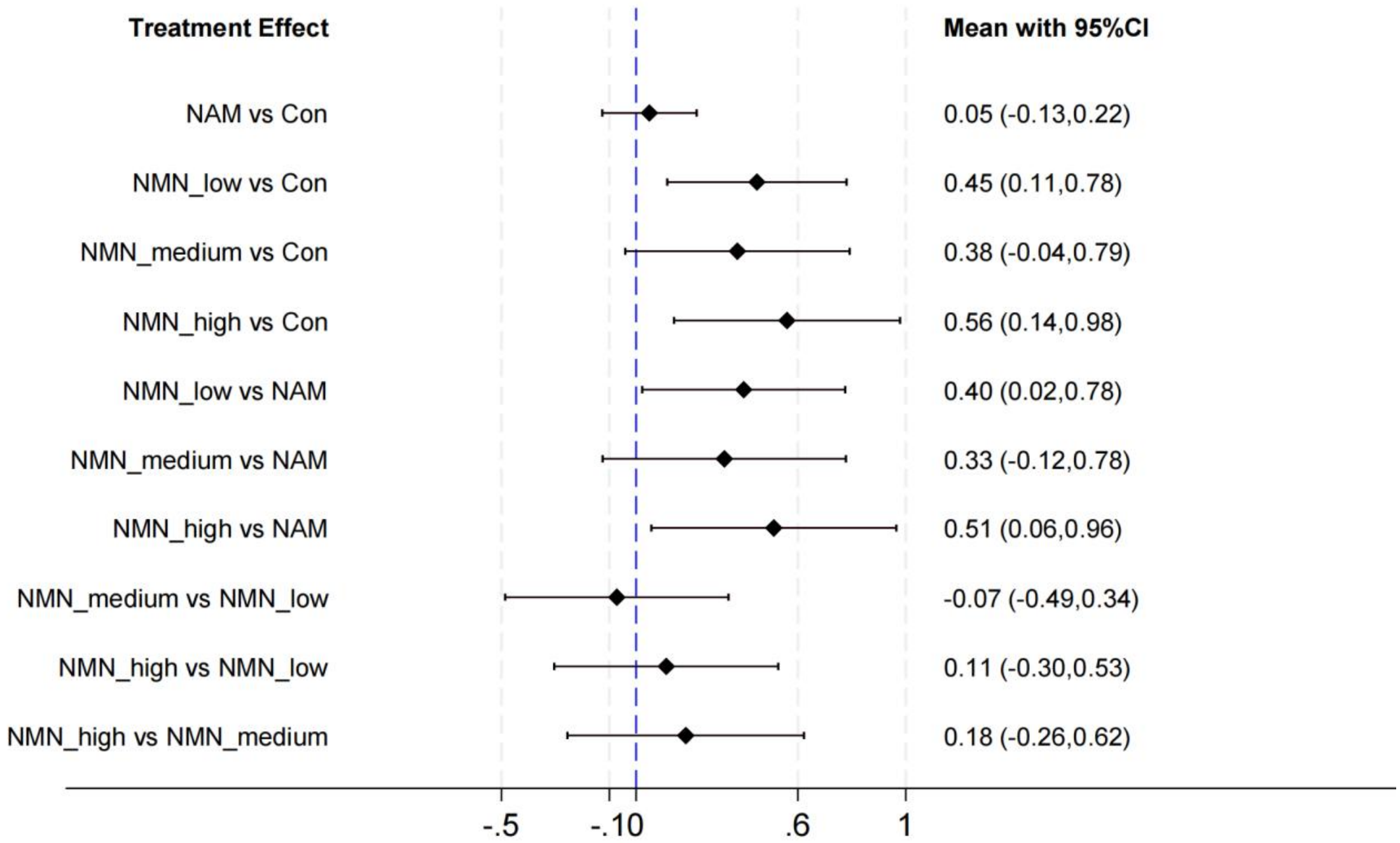

Figure S36 Interval plot of network meta-analysis for quality of life

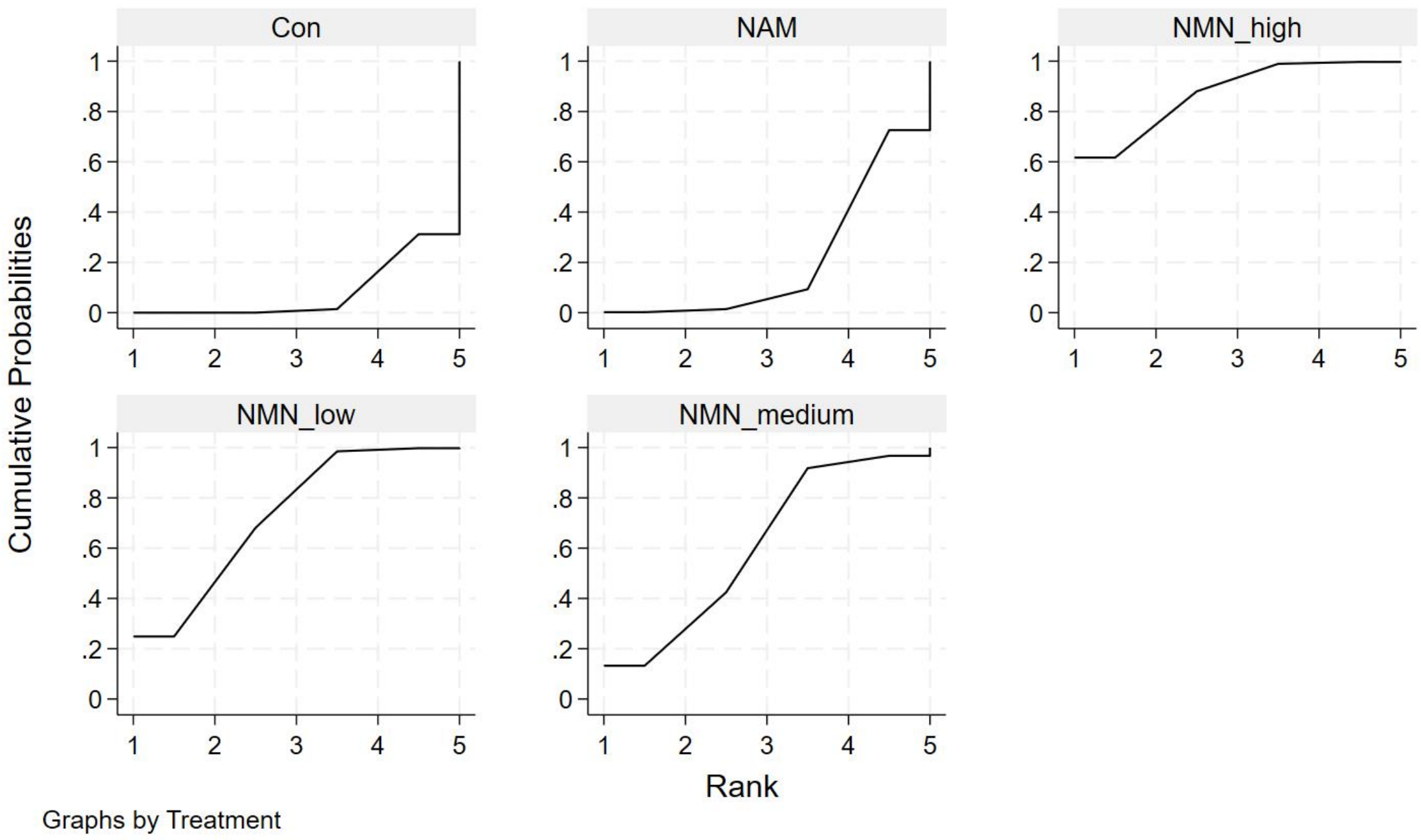

Figure S37 Cumulative ranking plot for quality of life

Table S14 The ranking of effects of different types of NAD+ precursors on quality of life

Treatment	Quality of life		
	SUCRA	Mean Rank	Probability of Best (%)
Control	8.2	0.0	4.7
NAM	20.9	0.2	4.2
NMN_low	72.8	24.9	2.1
NMN_medium	61.1	13.2	2.6
NMN_high	87.1	61.7	1.5

4.5.3 Publication Bias for Quality of life

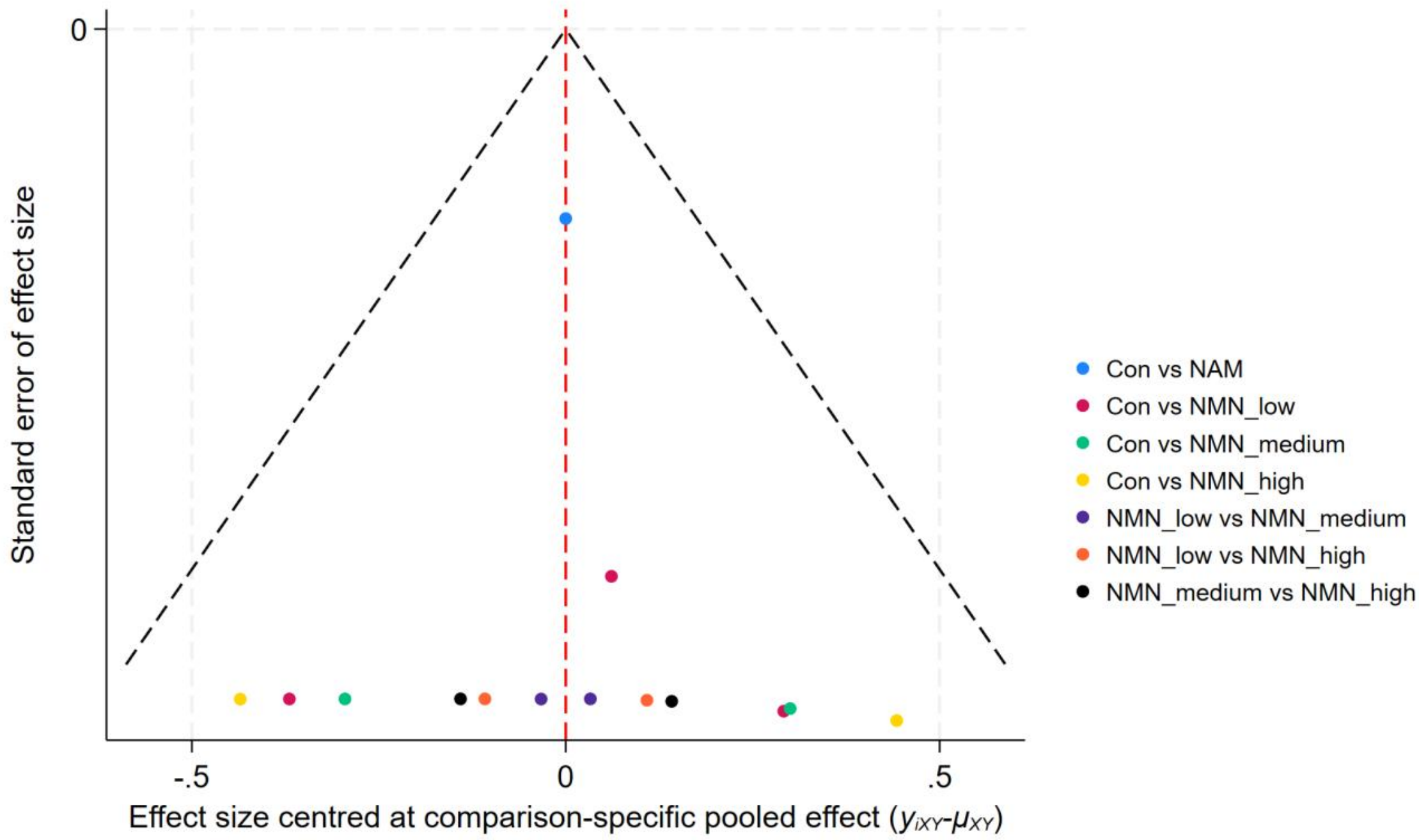

Figure S38 Funnel Plot for quality of life

4.6 Effects on Sleep Quality

4.6.1 Pairwise analysis for PSQI

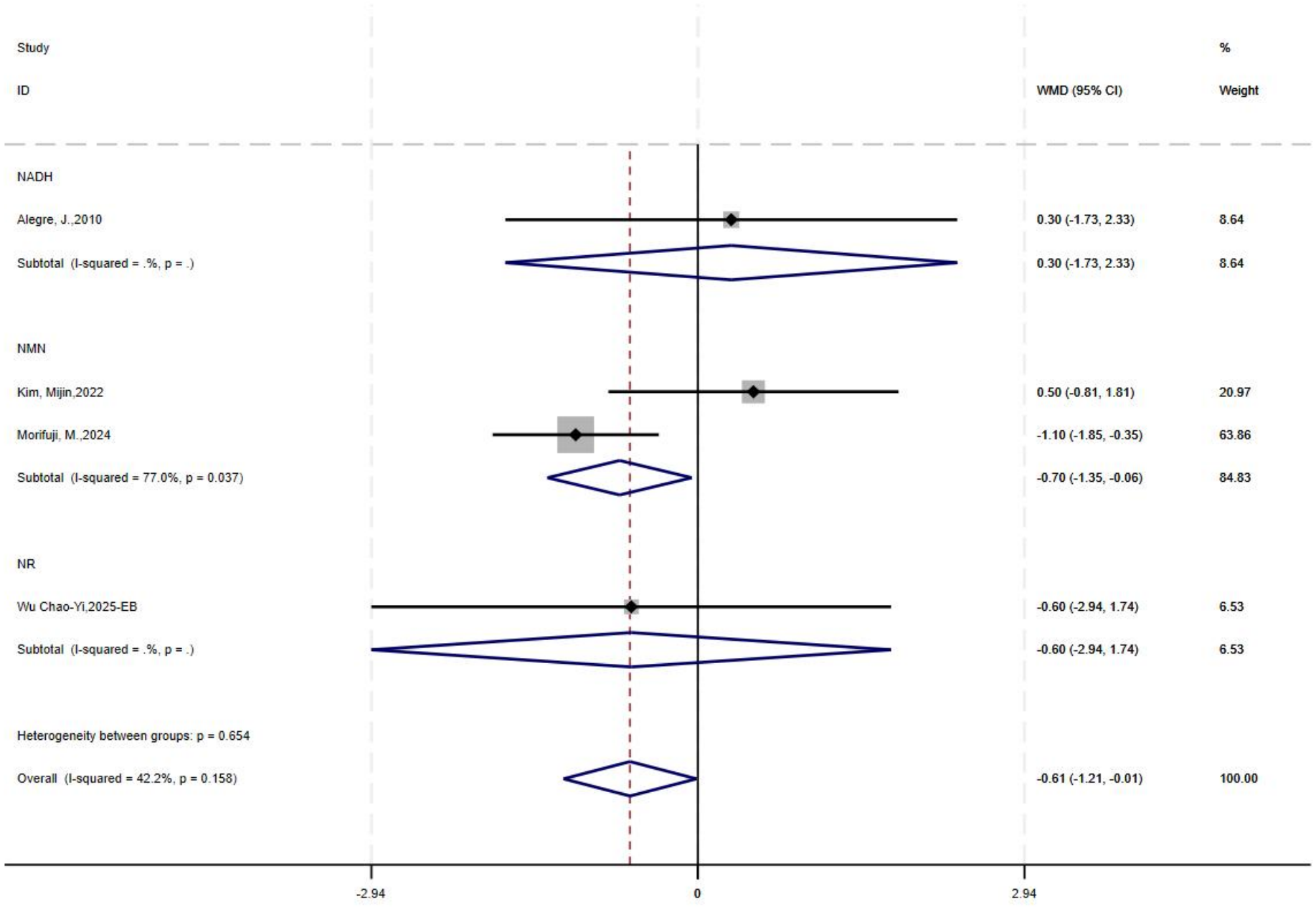

Figure S39 Forest plot of pairwise meta-analysis for PSQI

4.6.2 Publication Bias for PSQI

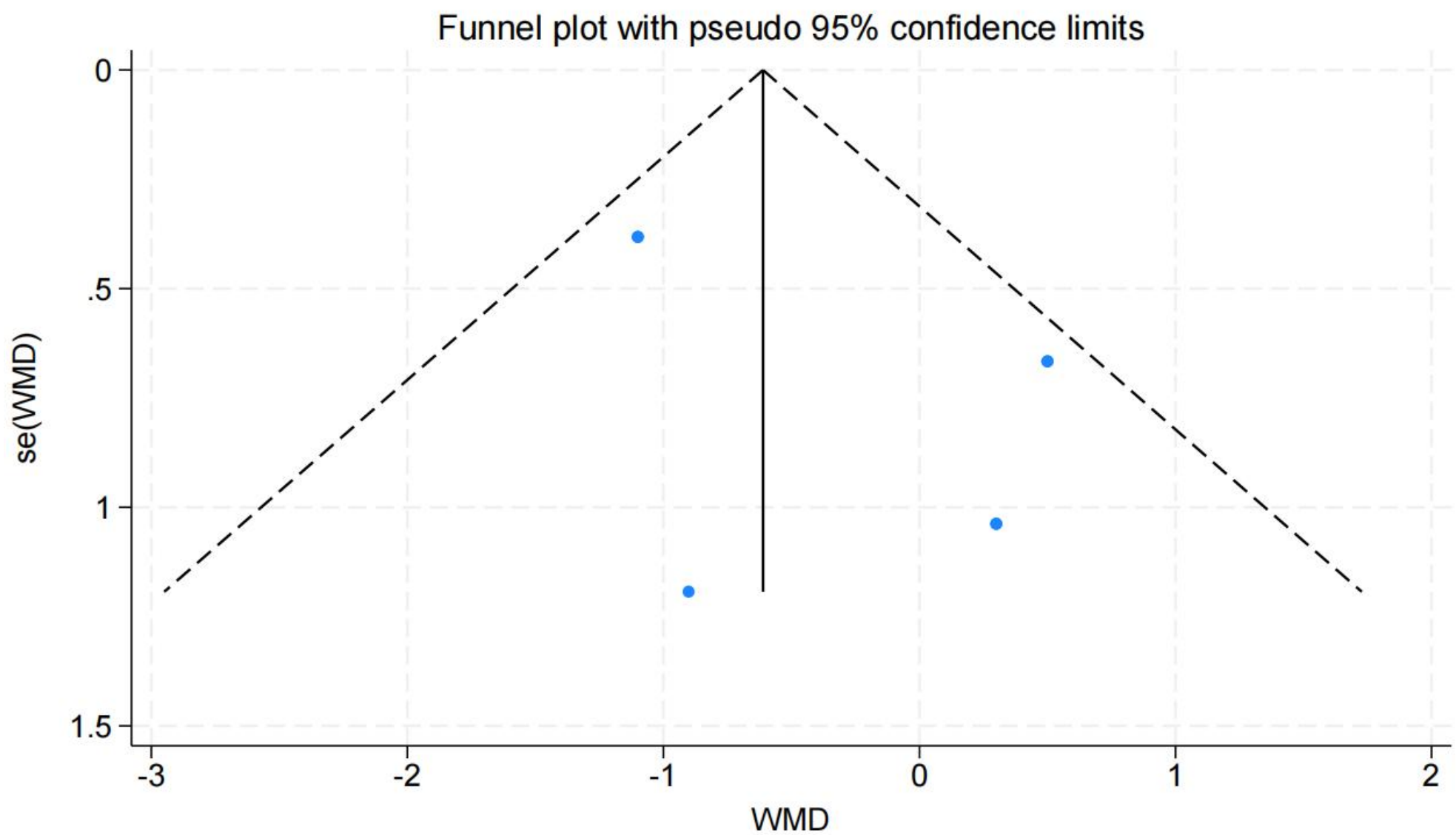

Figure S40 Funnel Plot for PSQI

5 Appendix 5 GRADE Evidence Quality Rating for NMA

Table S15 GRADE Evidence Quality Rating for NAD^+ Precursors Across Different Fatigue Domains

Outcome	Comparison	No. of Studies (Participants)	Risk of Bias	Inconsistency	Indirectness	Imprecision	Certainty
**Perceived Fatigue**	NAD^+ Precursors vs. Control	16 (1,126)	Some concerns [a]	Serious [b]	Not serious	**Serious** [c]	⊕⊕◯◯ **Low**
**Aerobic Capacity & Endurance**	NMN (High/Med) vs. Control	14 (540)	Low [d]	Not serious [e]	Not serious	Not serious	⊕⊕⊕⊕ **High**
**Cognitive Performance**	NADH vs. Control	24 (788)	Some concerns	Moderate [f]	Some concerns [g]	**Serious** [h]	⊕⊕◯◯ **Low**
**Neuromuscular Strength**	All Precursors vs. Control	20 (619)	Some concerns	Not serious [i]	Not serious	**Serious** [j]	⊕⊕◯◯ **Low**
**Quality of Life (QoL)**	NMN (High/Low) vs. Control	6 (722)	**Serious** [k]	Not serious [l]	Not serious	Not serious	⊕⊕⊕◯ **Moderate**
**Sleep Quality**	NAD^+ Precursors vs. Control	4 (231)	Some concerns	Not serious	Not serious	**Serious** [m]	⊕⊕◯◯ **Low**

Notes / Explanations (Statistical Rationale):

a **Downgraded by one level:** Approximately 33.3% of the included studies in this outcome were classified as having a moderate or high risk of bias, primarily due to incomplete outcome data.

d **No downgrading:** 66.7% of the studies across the entire review were classified as low risk; the core studies for this outcome demonstrated high methodological rigor.

k **Downgraded by one level:** Evidence of potential performance and selection bias due to a general lack of trial registration and incomplete reporting of pre-specified outcomes in NMN trials.

Inconsistency (I^2 & Heterogeneity)

b **Downgraded by one level:** High unexplained statistical heterogeneity (I^2 = 71.94%) and widely varying point estimates among different precursors.

e **No downgrading:** Statistical heterogeneity was negligible (I^2 = 0%, tau^2 = 0), and inconsistency tests showed no evidence of global or local discordance (*P* = 0.1457).

f **Downgraded by one level:** Moderate heterogeneity (I^2 = 63.1%) observed in the cognitive network, indicating variability in responses across different study populations.

i **No downgrading:** Heterogeneity was considered low to moderate (I^2 = 26.84%) and did not warrant further downgrading of certainty.

l **No downgrading:** Network analysis demonstrated no significant global inconsistency (*P* = 0.7313).

Indirectness

g **Downgraded by one level:** The observed benefits were primarily limited to clinical populations with baseline cognitive impairment (CI), limiting generalizability to healthy adults.

Imprecision (Confidence Intervals & Robustness)

c **Downgraded by one level:** The 95% CI (-0.80 to 0.05) crosses the null threshold; sensitivity analysis indicated that the primary network results lack robustness after excluding influential NADH trials.

h **Downgraded by one level:** The large effect estimate (SMD = 1.50) was highly unstable; exclusion of specific trials (Birkmayer et al., 2002) significantly reduced the pooled effect.

j **Downgraded by one level:** Imprecise estimates where all 95% CIs crossed the null (e.g., NMN: -0.06 to 0.43), and effect estimates were considered clinically negligible.

m **Downgraded by one level:** Analyzed via pairwise meta-analysis only due to sparse data; the sample size does not meet the optimal information size, and the CI crosses the null

6 Appendix 6 PRISMA NMA Checklist of Items to Include When Reporting A Systematic Review Involving a Network Meta-analysis

Section/Topic	Item #	Checklist Item	Reported on Page #
**TITLE**			
Title	1	Identify the report as a systematic review *incorporating a network meta-analysis (or related form of meta-analysis).*	Title page
**ABSTRACT**			
Structured summary	2	Provide a structured summary including, as applicable:<br>**Background:** main objectives<br>**Methods:** data sources; study eligibility criteria, participants, and interventions; study appraisal; and *synthesis methods, such as network meta-analysis.*<br>**Results:** number of studies and participants identified; summary estimates with corresponding confidence/credible intervals; *treatment rankings may also be discussed. Authors may choose to summarize pairwise comparisons against a chosen treatment included in their analyses for brevity.*<br>**Discussion/Conclusions:** limitations; conclusions and implications of findings.<br>**Other:** primary source of funding; systematic review registration number with registry name.	Abstract
**INTRODUCTIO N**			
Rationale	3	Describe the rationale for the review in the context of what is already known*, including mention of why a network meta-analysis has been conducted.*	1.Introduction (para. 1-6)
Objectives	4	Provide an explicit statement of questions being addressed, with reference to participants, interventions, comparisons, outcomes, and study design (PICOS).	1.Introduction (para. 7)
**METHODS**			
Protocol and registration	5	Indicate whether a review protocol exists and if and where it can be accessed (e.g., Web address); and, if available, provide registration information, including registration number.	2.1 Protocol and Registration
Eligibility criteria	6	Specify study characteristics (e.g., PICOS, length of follow-up) and report characteristics (e.g., years considered, language, publication status) used as criteria for eligibility, giving rationale. *Clearly describe eligible treatments included in the treatment network, and note whether any have been clustered or merged into the same node (with justification).*	2.3 Eligibility Criteria

Information sources	7	Describe all information sources (e.g., databases with dates of coverage, contact with study authors to identify additional studies) in the search and date last searched.	2.2 Search Strategy
Search	8	Present full electronic search strategy for at least one database, including any limits used, such that it could be repeated.	2.2 Search Strategy; Supplementary Appendix 1
Study selection	9	State the process for selecting studies (i.e., screening, eligibility, included in systematic review, and, if applicable, included in the meta-analysis).	2.3 Eligibility Criteria
Data collection process	10	Describe method of data extraction from reports (e.g., piloted forms, independently, in duplicate) and any processes for obtaining and confirming data from investigators.	2.4 Data Extraction and Quality Assessment
Data items	11	List and define all variables for which data were sought (e.g., PICOS, funding sources) and any assumptions and simplifications made.	2.3 Eligibility Criteria
**Geometry of the network**	**S1**	Describe methods used to explore the geometry of the treatment network under study and potential biases related to it. This should include how the evidence base has been graphically summarized for presentation, and what characteristics were compiled and used to describe the evidence base to readers.	2.5 Statistical Analysis 1. Network construction and synthesis
Risk of bias within individual studies	12	Describe methods used for assessing risk of bias of individual studies (including specification of whether this was done at the study or outcome level), and how this information is to be used in any data synthesis.	2.4 Data Extraction and Quality Assessment (para. 2)
Summary measures	13	State the principal summary measures (e.g., risk ratio, difference in means). *Also describe the use of additional summary measures assessed, such as treatment rankings and surface under the cumulative ranking curve (SUCRA) values, as well as modified approaches used to present summary findings from meta-analyses.*	2.5 Statistical Analysis
Planned methods of analysis	14	Describe the methods of handling data and combining results of studies for each network meta-analysis. This should include, but not be limited to:<br>• *Handling of multi-arm trials;*<br>• *Selection of variance structure;*<br>• *Selection of prior distributions in Bayesian analyses; and*<br>• *Assessment of model fit.*	
**Assessment of Inconsistency**	**S2**	Describe the statistical methods used to evaluate the agreement of direct and indirect evidence in the treatment network(s) studied. Describe efforts taken to address its presence when found.	
Risk of bias across studies	15	Specify any assessment of risk of bias that may affect the cumulative evidence (e.g., publication bias, selective reporting within studies).	
Additional analyses	16	Describe methods of additional analyses if done, indicating which were pre-specified. This may include, but not be limited to, the following:<br>• Sensitivity or subgroup analyses;	

- Meta-regression analyses;
- *Alternative formulations of the treatment network; and*
- *Use of alternative prior distributions for Bayesian analyses (if applicable).*

RESULTS†

Study selection	17	Give numbers of studies screened, assessed for eligibility, and included in the review, with reasons for exclusions at each stage, ideally with a flow diagram.	3.1 Study Selection
**Presentation of network structure**	**S3**	Provide a network graph of the included studies to enable visualization of the geometry of the treatment network.	3.4 Network Geometry Figure 2.
**Summary of network geometry**	**S4**	Provide a brief overview of characteristics of the treatment network. This may include commentary on the abundance of trials and randomized patients for the different interventions and pairwise comparisons in the network, gaps of evidence in the treatment network, and potential biases reflected by the network structure.	3.4 Network Geometry
Study characteristics	18	For each study, present characteristics for which data were extracted (e.g., study size, PICOS, follow-up period) and provide the citations.	3.2 Study Characteristics; Supplementary Appendix 2
Risk of bias within studies	19	Present data on risk of bias of each study and, if available, any outcome level assessment.	3.3 Risk of bias; Supplementary Appendix 3
Results of individual studies	20	For all outcomes considered (benefits or harms), present, for each study: 1) simple summary data for each intervention group, and 2) effect estimates and confidence intervals. *Modified approaches may be needed to deal with information from larger networks.*	3.5 -3.7; Supplementary Appendix 4
Synthesis of results	21	Present results of each meta-analysis done, including confidence/credible intervals. *In larger networks, authors may focus on comparisons versus a particular comparator (e.g. placebo or standard care), with full findings presented in an appendix. League tables and forest plots may be considered to summarize pairwise comparisons.* If additional summary measures were explored (such as treatment rankings), these should also be presented.	3.5 -3.7
**Exploration for inconsistency**	**S5**	Describe results from investigations of inconsistency. This may include such information as measures of model fit to compare consistency and inconsistency models, *P* values from statistical tests, or summary of inconsistency estimates from different parts of the treatment network.	Supplementary Appendix 4
Risk of bias across studies	22	Present results of any assessment of risk of bias across studies for the evidence base being studied.	3.3 Risk of bias; Supplementary Appendix 2

Results of additional analyses	23	Give results of additional analyses, if done (e.g., sensitivity or subgroup analyses, meta-regression analyses*, alternative network geometries studied, alternative choice of prior distributions for Bayesian analyses,* and so forth).	3.5 -3.7; Supplementary Appendix 4
**DISCUSSION**			
Summary of evidence	24	Summarize the main findings, including the strength of evidence for each main outcome; consider their relevance to key groups (e.g., healthcare providers, users, and policy-makers).	4.1 Principal Findings
Limitations	25	Discuss limitations at study and outcome level (e.g., risk of bias), and at review level (e.g., incomplete retrieval of identified research, reporting bias). *Comment on the validity of the assumptions, such as transitivity and consistency. Comment on any concerns regarding network geometry (e.g., avoidance of certain comparisons).*	4.5 Strengths, limitations, and future research directions
Conclusions	26	Provide a general interpretation of the results in the context of other evidence, and implications for future research.	5.Conclusions
**FUNDING**			
Funding	27	Describe sources of funding for the systematic review and other support (e.g., supply of data); role of funders for the systematic review. This should also include information regarding whether funding has been received from manufacturers of treatments in the network and/or whether some of the authors are content experts with professional conflicts of interest that could affect use of treatments in the network.	6.Declarations

PICOS = population, intervention, comparators, outcomes, study design.

* Text in italics indicateS wording specific to reporting of network meta-analyses that has been added to guidance from the PRISMA statement.

† Authors may wish to plan for use of appendices to present all relevant information in full detail for items in this section.
